# Supplementary material for: Global literature review and survey of implementation constraints on natural climate solutions
Source: Nat Commun. 2026 Mar 14;17:4580. doi: 10.1038/s41467-026-70482-4 (PMC13195100; doi:10.1038/s41467-026-70482-4)
Supplement: Supplementary file 1 — Supplementary Information [file 41467_2026_70482_MOESM1_ESM.pdf]

Supplementary Information for

Global literature review and survey of implementation constraints on natural climate solutions

Timm Kroeger* *et al.*

*Corresponding author. Email: tkroeger@tnc.org

This PDF file includes:

Supplementary Text
Figs. S1 to S21
Tables S1 to S3
Survey questionnaire
Supplementary text references

Supplementary Text

Constraint terminology and categorization

The literature on NCS constraints is inconsistent in both terminology and categorization. For example, what we term 'constraint categories' is variously referred to as 'feasibility dimensions'[1], 'barriers and opportunities'[2], 'categories of enabling factors'[3] or 'barriers'[4], to name but a few. The classification of individual constraints into these categories and the thematic scope and terminology used for the categories themselves also show a considerable degree of diversity (Table S2). For example, Ref. 4 classifies 'lack of credit access' as a 'technological' barrier, while Ref. 3 classifies the somewhat broader 'financial services' as a 'financial' enabling factor, and Ref. 2 classifies 'scale and accessibility of financing' as a 'socio-economic' barrier or opportunity.

We classified the unique constraint observations obtained through our re-coding of Brumberg et al.'s (2025) systematic literature review dataset and through our NCS project survey into 46 constraints (Table 1). We developed these constraints iteratively through emergent coding, and the wording of each was chosen such that it encompasses the range of specific permutations of each constraint found in the reviewed papers (Table S1). We then grouped these constraints into eight categories (Table S2). Market constraints arise from a lack of, or a lack of access to markets (due to absence of markets or high transaction costs), or a lack of rentability of NCS (due to insufficient prices of NCS outputs, carbon, ecosystem services, or biodiversity). Finance constraints take the form of a lack of land manager or project access to financial resources or services (credit or insurance), or demanding reporting requirements associated with obtaining funding or financing for NCS. Knowledge constraints are caused by a lack of information (about the design, establishment, management, biophysical or economic performance, or climate mitigation, or on-site benefits of NCS, either in the scientific community or among land managers; or about market access), lack of technical advice, or lack of land managers' ability to effectively implement the NCS (due to land manager limitations in terms of literacy, numeracy, or technological capacity). Social-behavioral constraints are caused by social norms, behaviors, preferences or attitudes (e.g., disinterest in or skepticism of NCS or of NCS promoters), lack of social learning or exchange networks, discrimination against certain groups, disagreement among actors or groups, equity concerns, or challenges in engaging or coordinating with relevant stakeholders. Rules and Laws comprise legal or other formal principles that negatively affect NCS implementation, such as lack of secure tenure or resource management or use rights, regulatory barriers, lack of legal or policy clarity, insecure or uncertain benefit sharing; or incentives for competing land uses. Government and Organizations can constrain NCS implementation through the behaviors (favoring non-NCS production, lack of enforcement of laws, policies, regulations, or contracts) or lack of coordination or implementation capacity of state or non-state entities, or through governments' inability to prevent violent conflict or the threat of violence. Material Inputs constraints arise from lack of needed NCS production inputs (e.g., seedlings, fertilizer, specialized tools), labor, water, or suitable land. Finally, Negative Side Effects such as reductions in the yield of other crops (despite increased profitability of NCS), increased physical difficulty of land management, negative health impacts, property damage, or conflicts with wildlife or neighbors can constrain NCS implementation (Table S1). Despite some differences in the categorization and naming of select constraints, our classification is well-aligned with existing ones (Table S2).

Specific differences between our and others' constraint terminology and categorization

Throughout this paper, the term 'constraint' refers to any of the 46 unique constraints that we developed through our combined a priori and emergent coding. Each of these constraints was developed iteratively to capture the often-diverse observations across journal articles that used different language to describe the same constraint. Due to differences in the description and interpretation of constraints observed in the literature, the classifications of all observed constraints were reviewed by at least three authors for consistency.

Below, we discuss how our constraint classification differs from those used in four other recent NCS studies[1–4]. Table S2 identifies congruence between individual constraints in our and the other classifications. The table shows broad alignment in terminology between our constraint categories and those of the other four studies. One notable difference is that we refer to 'knowledge' rather than 'technological' or 'technical' constraints because knowledge better captures the informational nature of some of the included constraints (e.g., lack of knowledge about NCS design, management, or performance; either in general or on the part of land managers). Furthermore, like Ref. 3 but unlike Ref. 2, Ref. 4 and Ref. 1, we distinguish between the formal or informal institutional frameworks of policies, laws, rules, and regulations that affect NCS implementation (our 'rules and laws' category), and the attitudes, behaviors or capacities of governmental or non-governmental entities (Government and Organizations)– including attitudes, behaviors or capacities related to the implementation, or lack thereof, of policies, laws, rules or regulations – that constrain NCS implementation. We believe this distinction is useful and important as the actions needed to overcome these two categories of constraints are fundamentally different (effecting change in policies, laws, rules, regulations vs effecting change in the behavior or capacities of individuals or entities).

We do not discuss in detail the agreement or differences between each of our constraints and specific constraints in the other classifications. Rather, when one of our constraints is identical with, or substantively similar to, specific constraints in the other classifications, this is indicated in Table S2 by the same Arabic numerals (shown in parentheses). Where there is limited congruence, this is indicated by italicized Arabic numerals.

Ref. 3 categorizes NCS 'enabling factors' based on Ref. 5. While most of the 'enabling factors' identified in Ref. 3 are reflected in our constraints, some are not. The latter is due primarily to two factors: a substantive difference between Ref. 3's 'enabling factors' and our constraints, and differences in the parsing of constraints into their constituent components or sub-constraints.

Enabling factors as defined in Ref. 3 are conditions that are positively correlated with NCS implementation in the literature. In contrast, our constraints are factors that in the literature are reported as impeding NCS implementation. While in many cases Ref. 3's 'enabling factors' represent the inverse of constraints, this is not always true. For example, our classification does not include three of Ref. 3's 'biophysical' enabling factors (favorable climatic conditions, species diversity, and carbon stock potential). Since enabling factors as defined in Ref. 3 are conditions that are reported as being positively correlated with NCS implementation, it is not surprising that, for example, high potential carbon stocks or high biodiversity are positively correlated with NCS because of the potential income they might generate (carbon) or the

financing they might attract (biodiversity). However, lower potential carbon stocks are not necessarily a constraint on NCS implementation, because an NCS may deliver other desired outputs for potential implementers. Where low carbon stock potential is indeed identified in a paper as a constraint on NCS implementation, its negative effect on NCS implementation is captured by our constraints related to carbon market access or carbon prices. The same applies to biodiversity, lower levels of which may not necessarily prevent the attractiveness of an NCS to potential adopters – say, farmers or ranchers who consider agroforestry adoption for productivity gains, increased climate resilience, or carbon income. Where low biodiversity prevents the feasibility of an NCS project, it will do so via lack of access to biodiversity markets or low prices on those markets, two constraints we capture separately.

An example of a difference in parsing of constraints is Ref. 3's 'delivery of benefits' as an enabling factor, where delivery of benefits is defined as the sufficient size of overall benefits, or net welfare (i.e., not just financial) gains[3]. We do not have a corresponding inverse constraint 'lack of benefits' or 'insufficient benefits.' Rather, we have separate constraints for factors that individually or in combination may result in benefits that are insufficient to lead to NCS adoption, such as inadequate markets or prices for NCS products, carbon, biodiversity, or ecosystem services, or negative side effects (e.g., human-wildlife conflict or health or property damage as a result of NCS).

We also do not have direct equivalents for Ref. 3's social NCS enabling factors of IPLC engagement, stakeholder consultation, recognition of traditional values, and FPIC. Presence of these enabling factors during project design and implementation ensures appropriate local consultation and increases the likelihood that project designs meet local desires and preferences. While we do not explicitly account for the lack of these factors as constraints, their absence is likely to result in several of our 'social and behavioral' constraints, specifically, local preferences for non-NCS land uses, skepticism or disinterest in NCS or lack of trust in NCS promoters; lack of opportunity to participate in or influence the implementation of NCS; and concerns over negative equity impacts.

Finally, there are some differences in categorizations between Ref. 3 and our analysis. For example, Ref. 3 classify lack of availability of qualified government personnel as an economic constraint (as explained in their paper, this is included as part of their "labor availability" constraint). We classify lack of government capacity under our Government and Organizations category, specifically, the constraint 'lack of policy coordination or implementation capacity.'

Ref. 2's 'socio-economic barriers and opportunities' category includes finance mechanisms, funding, and incentives, which we capture in our Markets and Finance categories; risk and uncertainty, which we capture in our Markets, Knowledge and Social-behavioral categories; impacts on poverty or food security, the latter of which we capture in our Negative Side Effects category; and cultural values and social acceptance, which we bin into our Social-behavioral category. IPCC's institutional barriers and opportunities include transparent and accountable governance (accounted for in our Government and Organizations category), clear land tenure and land-use rights (our Rules and Laws category), and institutional capacity (our Government and Organizations category). IPCC's ecological barriers and opportunities category includes land and water availability (in our Material Inputs category); uncertainties and differences in outcomes related to specific soil conditions, water availability, GHG reduction potential as well as natural

variability and resilience (captured to various degrees in our Knowledge category), and adaptation and biodiversity benefits (captured in our Knowledge category). Their technological barriers and opportunities category consists of monitoring, reporting, and verification (MRV) needs, which we capture in our Finance category ('burdensome reporting requirements') and our Knowledge category ('availability of technical advice for land managers'). In general, our substantially larger number of constraints is largely due to our finer parsing of constraints.

Ref. 4 identifies socio-cultural 'barriers' that comprise norms and values, knowledge and perception, and behavior 'sub-barriers,' with 'descriptions' of specific finer barriers within each sub-barrier. These barriers generally are captured in our Knowledge and Social-behavioral categories. Ref. 4's technological barriers include complexity (difficult to adopt activity; requiring high management skills), resources (access to specialized machinery, lack of transport infrastructure, lack of inputs, limited/no access to credit, limited extension facilities); and development (lack of MRV; larger uncertainties about benefits, limited understanding of land suitability; technological readiness). These are captured in our Markets, Finance, Knowledge, and Material Inputs categories. Ref. 4's economic barriers include costs (unable to afford specialized machinery; large initial investment, expensive to deploy at a scale where there is large potential), income (potential for income decline due to trade-offs; transitional period with higher production costs and lower income; lack of incentives), and value (difficulty of monetizing non-market benefits/ES), most of which are captured in our Markets category. Their institutional barriers include policy barriers (lack of policy support mechanism to set explicit incentives; lack of policy implementation; disinterest of policymakers) that we capture in our Government and Organizations and Rules and Laws categories; governance barriers (lack of cross-sectoral responsibility sharing; top-down approach; coordination between stakeholders, lack of proper monitoring) some of which we capture in our Government and Organizations category; and regulation-related barriers (counter-productive public policies and legislation; lack of standards and protocols to measure C sequestration) that we capture in our Rules and Laws category. Ref. 4's ethical barriers comprise challenges related to conflict (risks of land grabbing; equitable benefit sharing; social conflicts) and fairness (limited access of women and minority groups to resources and land; no consideration of the rights of Indigenous People and local communities) that we capture in our Social-behavioral and Rules and Laws categories, and trade-offs (land availability and competition with other land uses; possible increase in food prices and compromise food security; negative effect on the environment), which we capture in our Negative Side Effects category.

Ref. 1 identified macro-level 'feasibility indicators' to assess the country-level feasibility of terrestrial NCS for the world. Unavoidably, this resulted in the selection of indicators characterized by geographically broad data availability. Some of Ref. 1's indicators are fairly similar to ours, while others represent country-wide characteristics that arguably are positively related to NCS feasibility but whose absence doesn't directly translate into specific constraints (e.g., GDP per capita, agricultural value added, personal rights, agricultural total factor productivity, or ease of doing business).

Recoding of Brumberg et al.'s[6] constraints

The literature portion of our dataset utilizes Ref. 6's systematic review. However, we use 46 constraints in our analysis while Ref. 6 used 39. In most cases, the constraints used in Ref. 6

match ours verbatim or in meaning. Where this was not the case, we full-text reviewed the respective studies to ensure correct recoding to our constraints. Specifically, observations for the following constraints in Ref. 6 were recoded as described below:

Ineffective laws, policies and regulations: In most cases, these challenges represented instances of lack of policy coordination or implementation capacity or of unclear laws and policies related to NCS outputs/markets and were recoded to our respective constraints.

Lack of laws, policies and regulations: In almost all cases, this constraint was recoded to unclear laws and policies related to NCS outputs/markets.

Trade-offs with agriculture: this recoded to either Negative side effects of NCS or greater profitability of alternative land uses, depending on whether the concern was that NCS reduced yield of the traditional (baseline) crop (even while it might yield new products) or overall farm-level net revenue, respectively.

Trade-offs with other land uses: All instances of this constraint described negative impacts on human health, property damage, increased complexity or difficulty of land management (that did not affect profitability), negative biodiversity impacts, conflict with neighbors, or conflict with wildlife attracted by the NCS and were recoded to our constraint category Negative side effects of NCS; or they referred to concern about potentially undesirable negative equity impacts, in which case they were recoded to our constraint Concerns about negative equity impacts.

Corruption or lack of transparency: in the relatively few cases where corruption was the challenge, we recoded to NCS-related corruption; in most cases, the issue was lack of transparency, specifically, lack of transparency that led to, or was feared to, result in inequitable outcomes. We coded those cases as concerns over negative equity impacts of NCS.

Lack of political will for NCS: this was recoded to politically influential interests favoring non-NCS.

Insecure land tenure: we distinguish between land ownership and the right to management of land assets, because the two do not always align (e.g., in several countries, land ownership does not convey the right to harvest planted trees), and recoded observations to the applicable tenure constraint.

Human-wildlife conflict: recoded to Negative side effects of NCS if the concern was that wildlife attracted by the NCS posed a risk to human health or property, or Skepticism or disinterest in NCS or lack of trust in NCS promoters if the concern was that wildlife was damaging and making questionable the feasibility of the NCS itself.

Interpersonal conflict: recoded to Lack of dispute resolution if the issue was disagreements over the NCS in question itself, or difficulty identifying, engaging, or coordinating with relevant actors if the inherent challenge was logistical in nature.

Time lag in NCS benefits: in all cases, the issue was that delayed benefits from NCS negatively affected the expected profitability of NCS, so we recoded this to greater profitability of alternative land uses.

Brumberg et al. [6] combined several NCS pathways that had low observation counts in their analysis: AWC and CWR; PeR and APC; and AGC and GrR, respectively. Because of the higher observation count in the combined literature and survey data, we considered each of these pathways individually and recoded the papers featuring these pathways to the appropriate pathway.

Questionnaire development and distribution
The survey flier with links to the English, Spanish, French and Portuguese survey versions was distributed through their organizational networks by the authors and colleagues at The Nature Conservancy, Wildlife Conservation Society, World Resources Institute, Center for International Forestry Research (CIFOR) and World Agroforestry (ICRAF), Conservation International, Eden: People+Planet, Catholic Relief Services, EcoDecision, International Centre for Integrated Mountain Development, One Acre Fund, Heifer International, Foundation for Ecological Security, African Forest Forum, Ecoagriculture Partners, University of Florida at Gainesville, and University of Sao Paulo. The survey also was promoted and the survey flier included in several editions of the Restor (www.restor.eco) and Nature4Climate (https://nature4climate.org) online newsletters. It included a free and prior informed consent (FPIC) statement, did not collect private or sensitive information, and was approved under The Nature Conservancy's Human Subjects Research policy. Respondents had the option to remain anonymous.
Upon reviewing the FPIC script and agreeing to participate, respondents were asked to provide optional demographic information, organizational affiliation and position title, with space to record multiple respondents; describe the project for which they were completing the questionnaire (name; country; additional location information, with the option of using a map widget; size, start and end year, if any); identify the land ownership and land management types, respectively (government, private individual, private company, non-governmental organization, indigenous community, other local community, other); identify the NCS pathways and sub-pathways implemented (multiple selection); and indicate whether the project was pursuing, or had interest in pursuing in the future, carbon crediting and, if so, on which registry or market (multiple selection). Next, respondents were asked to select from the provided full list of constraints (Table 1) all that made it challenging to achieve the full project objectives ("Applies to my project"; "Does not apply..."; "Unsure"), with the option of writing in additional constraints. They were then presented with a list of the constraints they had selected or written in, and asked to rank the five most important ones, with "1" being the most important, "2" being the second-most important and so on. This was followed by drill-down questions for the identified top-five constraints that asked respondents to identify (1) the specific nature of the constraints as either lack of, or uncertainty about, "availability" or "quality"; uncertain or high "cost" of inputs; or uncertain or low "price" for marketed NCS outputs (Yes, No, Unsure); and (2) whether the constraint primarily affects land managers, project staff, the local community or government(s), district/county or state/province government(s), or national ministries or departments (as relevant to a given constraint) (multiple selection). Next, respondents were asked to identify key actions that they thought could address each of the top-five constraints and by whom, and whether any of these actions were currently being implemented or efforts were underway to do so. Respondents were then asked what they believed the ten-year scaling potential of their project to be if key constraints were addressed (single selection: unsure, none, double, triple, quadruple, more than quintuple in size). Finally, they were again presented with the full list of 46 constraints and asked to identify up to ten that currently are most important in preventing the scaling of their project.

Geocoding and data analysis
All data analysis was conducted in R[7] using the tidyverse, dplyr, and ggplot2 packages.

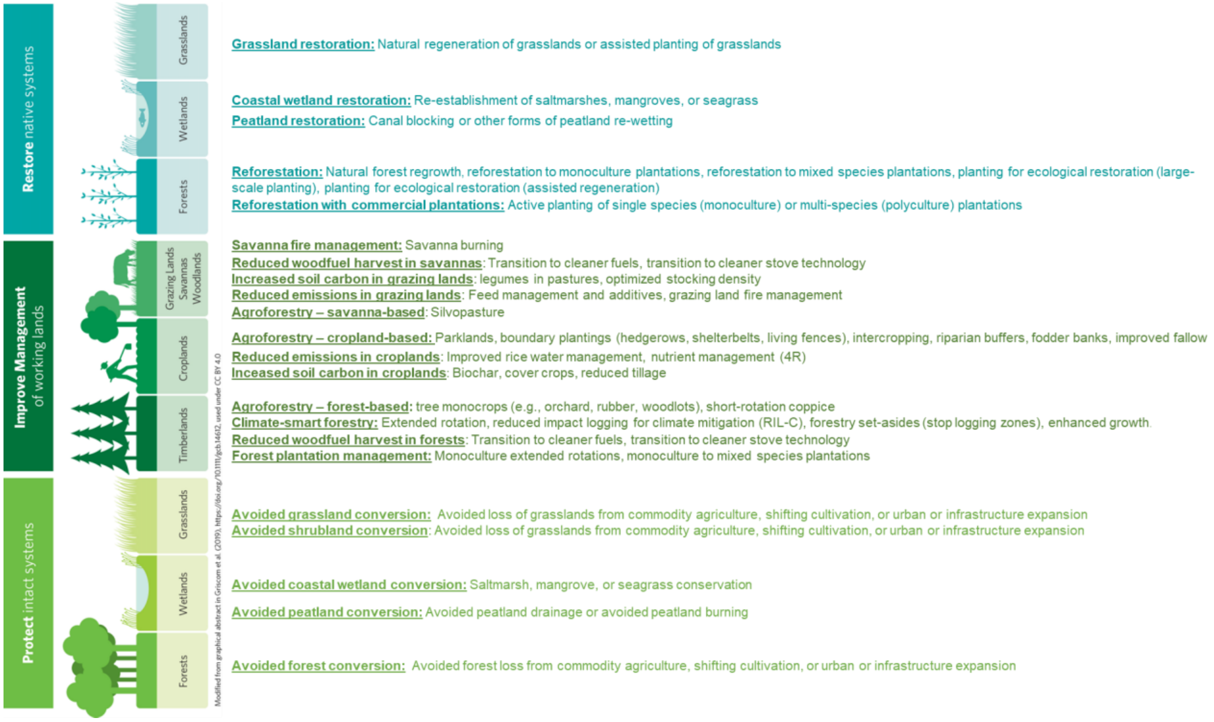

Fig. S1. List of Natural Climate Solutions pathways (shown in boldface and underlined) and sub-pathways included in the survey. Graphic on the left cropped and text modified from graphical abstract in Griscom et al. (2019), We need both natural and energy solutions to stabilize our climate, Global Change Biol. 25: 1889–1890, https://doi.org/10.1111/gcb.14612, published under a CC BY 4.0 Attribution 4.0 International license (https://creativecommons.org/licenses/by/4.0/). Pathways are grouped by NCS strategy (protect, restore, improve management) and biome (forests, wetlands, grasslands).

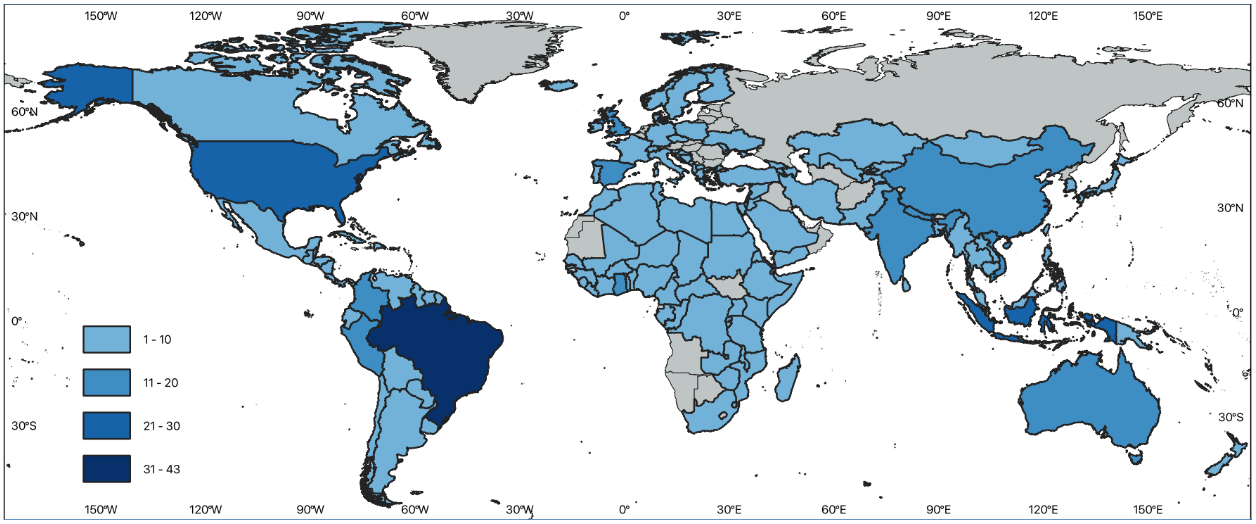

Fig. S2. Number of papers included in the analysis, by country. Colors indicate the number of published articles for each country that were included in the analysis. Basemaps are from Runfola, D. et al. (2020), geoBoundaries: A global database of political administrative boundaries, PLoS ONE 15(4): e0231866, https://doi.org/10.1371/journal.pone.0231866, published under a CC BY 4.0 Attribution 4.0 International license (https://creativecommons.org/licenses/by/4.0/).

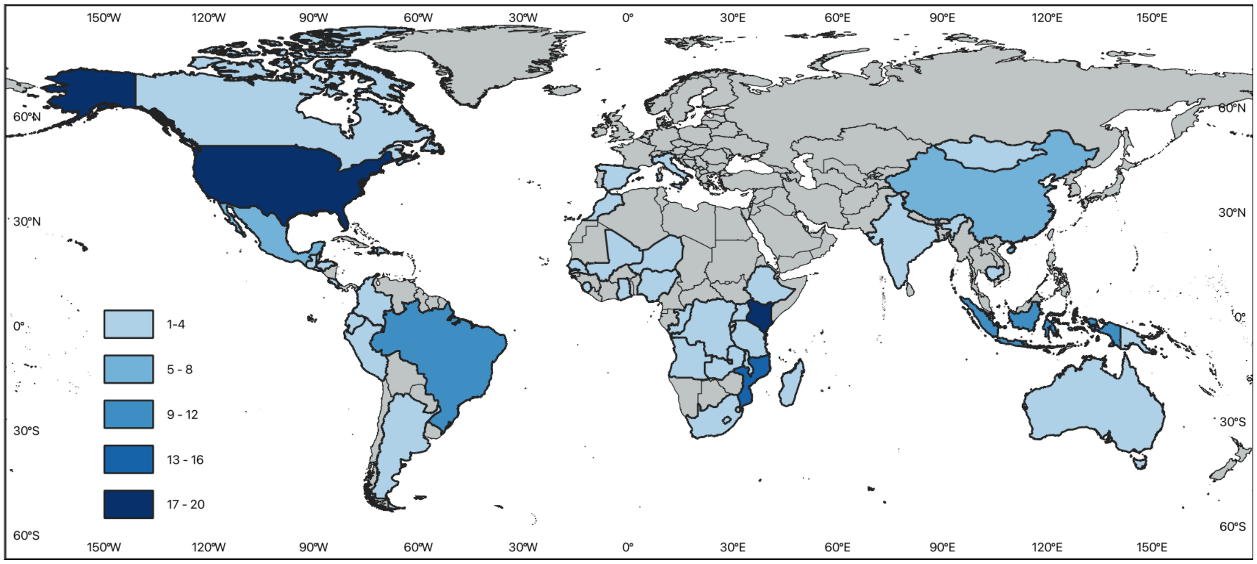

Fig. S3. Number of survey projects by country. Colors indicate the number of survey projects in each country that were included in the analysis. Basemaps are from Runfola, D. et al. (2020), geoBoundaries: A global database of political administrative boundaries, PLoS ONE 15(4): e0231866, https://doi.org/10.1371/journal.pone.0231866, published under a CC BY 4.0 Attribution 4.0 International license (https://creativecommons.org/licenses/by/4.0/).

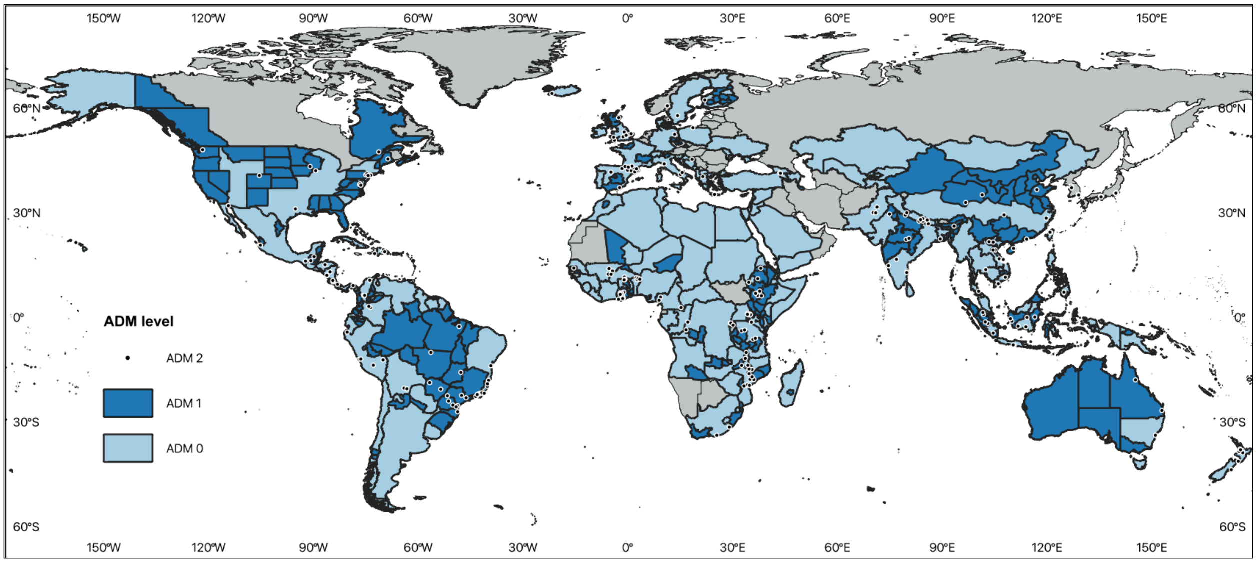

Fig. S4. Administrative (ADM) units covered by papers and survey projects, by administrative level. Colors indicate the administrative level 0 (national; ADM0) and administrative level 1 (largest sub-national; ADM1) units, and black dots the administrative level 2 (second-largest subnational level; ADM2) units for which we obtained information on natural climate solution implementation constraints through the literature review and global project survey. Map does not show number of papers or projects in an administrative unit. Basemaps are from Runfola, D. et al. (2020), geoBoundaries: A global database of political administrative boundaries, PLoS ONE 15(4): e0231866, https://doi.org/10.1371/journal.pone.0231866, published under a CC BY 4.0 Attribution 4.0 International license (https://creativecommons.org/licenses/by/4.0/).

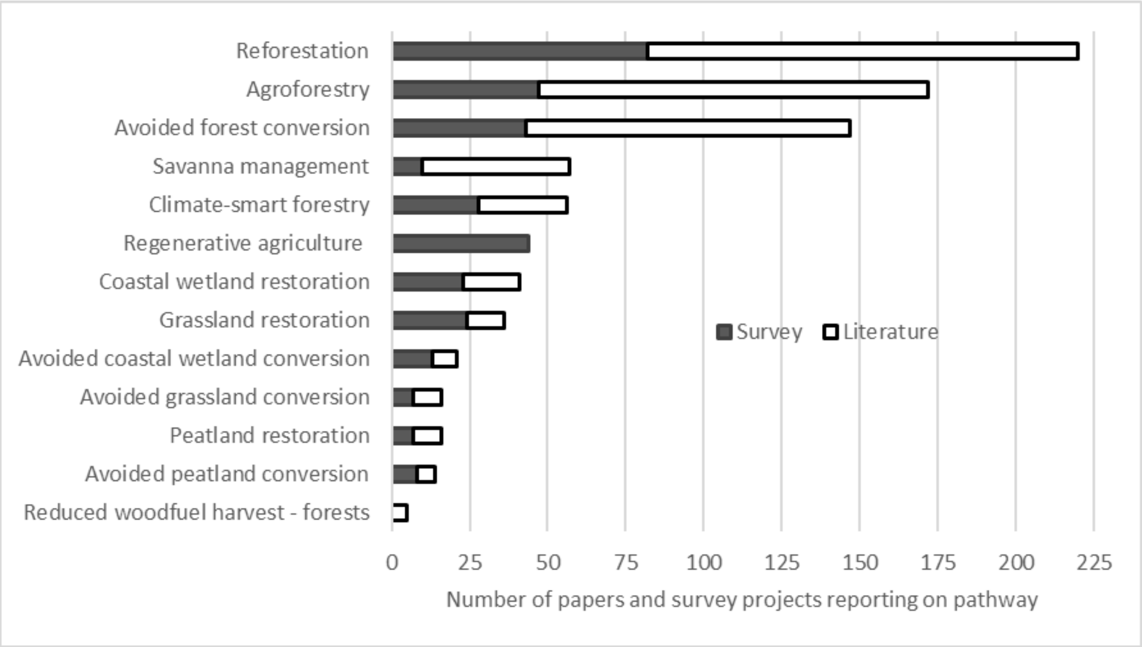

Fig. S5. Number of papers and survey projects reporting natural climate solutions implementation constraints for included pathways. Both the literature and survey samples show an uneven distribution across pathways. Forest pathways are most heavily represented followed by agricultural and grassland pathways.

Sub-region \ NCS_Pathway	Savanna management	Regenerative agriculture	Reforestation	Reduced woodfuel harvest-forests	Peatland restoration	Grassland restoration	Coastal wetland restoration	Climate-smart forestry	Avoided peatland conversion	Avoided grassland conversion	Avoided forest conversion	Avoided coastal wetland conversion	Agroforestry	Sub-region Total	Sub-region Share	Regional share
Northern America	6	3	22		3	4	6	11	3	7	7	3	8	83	9%	36%
Central America	6	6	16			1	3	3	1		20	4	13	73	8%	
South America	15	3	45	1	3	9	2	2	1	2	41	2	24	150	17%	
Caribbean	1	1	3			1	2	1			1	1	5	16	2%	
Western Asia	2		2				1					1	2	8	1%	21%
Central Asia			1										1	2	0%	
Eastern Asia	2	1	20			1	5	2		1	6	2	3	43	5%	
South-Eastern Asia	2		16		6		6	1	7		22	5	21	86	10%	
Southern Asia		2	12	1		1	2	5			8	2	14	47	5%	
Melanesia			1				3				1	2	1	8	1%	4%
Australia and New Zealand	1	1	7			1	4	2		2	4	2	3	27	3%	
Northern Africa	3	1	3				1	1			2	1	3	15	2%	30%
Western Africa	5	6	19	1		1	1	6	1	1	10	1	20	72	8%	
Eastern Africa	11	15	33	1		9	11	8	1	1	23	4	38	155	17%	
Central Africa	1		4					2	2		4	1	4	18	2%	
Southern Africa	1		2			2	1						1	7	1%	
Northern Europe	2		17		4	2	1	5		1			3	35	4%	10%
Western Europe	2		6			2		1		1	1		6	19	2%	
Eastern Europe	1		3					1			2		1	8	1%	
Southern Europe	6	2	7	1		2		2		1	3		6	30	3%	

Fig. S6. Number of papers and survey projects by subregion and pathway. A paper or survey project that covers more than one pathway, subregion, or location within a subregion results in multiple counts in this table. Grey shading indicates no data. A survey or paper that reports on more than one sub-pathway belonging to the same pathway is counted as a single observation (e.g., mangrove restoration and saltmarsh restoration are two sub-pathways of the pathway ‘coastal wetland restoration‘). Shading intensity is proportional to a subregion-pathway combination’s share in all papers or survey projects (red shading) or the subregional or regional shares in all papers or projects (green shading).

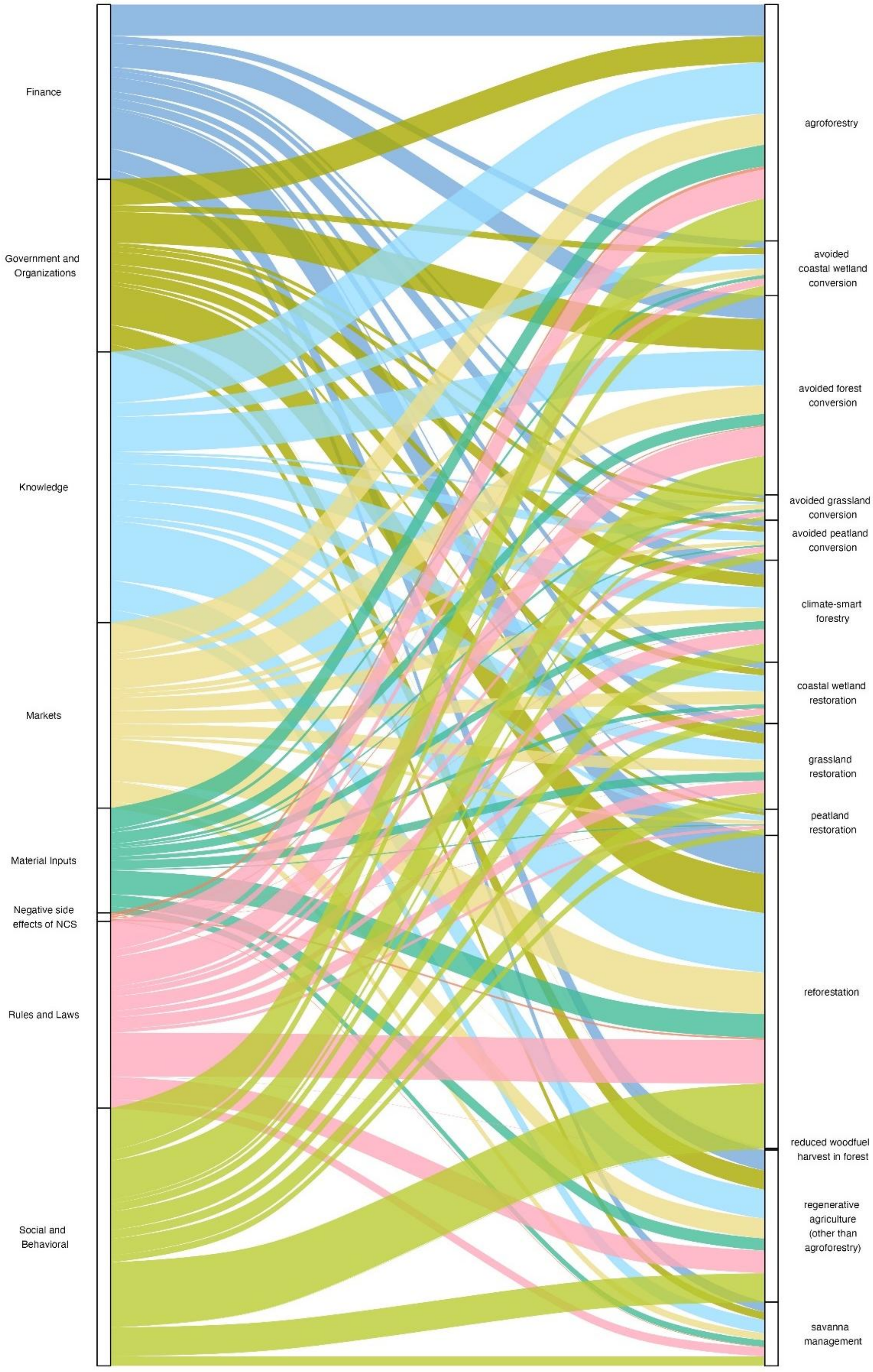

Fig. S7. Alluvial diagram showing the distribution of observations for each constraint category across pathways and the composition of constraint category observations for each pathway. Thickness of lines is proportional to number of constraint observations.

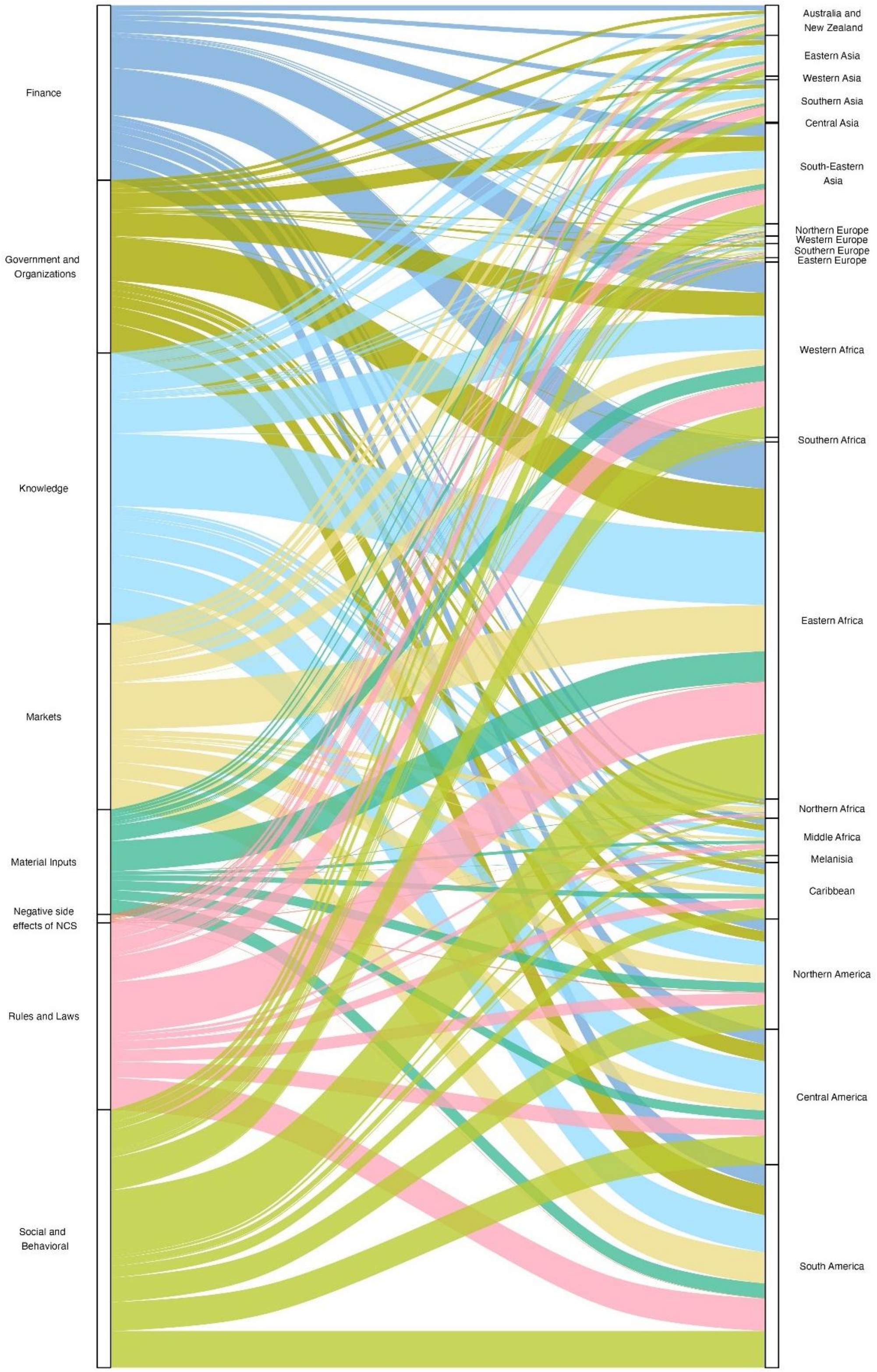

Fig. S8. Alluvial diagram showing the distribution of observations for each constraint category across subregions and the composition of constraint category observations for each subregion. Thickness of lines is proportional to number of constraint observations.

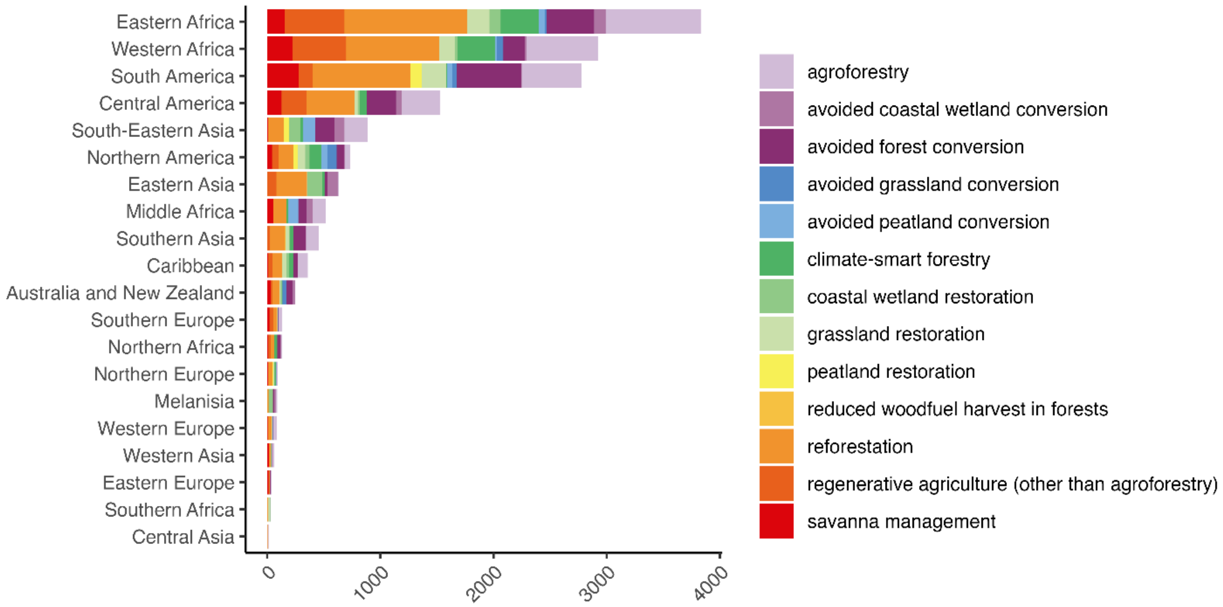

Fig. S9. Subregional distribution of constraint observations by NCS pathway. Nearly one-half (48%) of the 15,572 constraint observations in our dataset are from Africa, with the vast majority of these from Eastern (25%) and Western (19%) Africa (Fig. S16). South (18%) and Central (10%) America account for the next-largest shares of observations, followed by South-Eastern Asia (6%), Northern America (5%) and Eastern Asia (4%). Europe (all subregions combined), Australia-New Zealand, and Melanesia each account for only 2% of all observations. Reforestation is the pathway with the highest number of constraint observations in 15 of the 20 subregions. In all subregions except Australia-New Zealand and Melanesia, agroforestry contributes the second-highest number of constraint observations. Avoided forest conversion represents the third-largest number of observations in the Americas and Asia, but not in Africa, where regenerative agriculture and climate-smart forestry contribute the third and fourth-largest sets of observations, respectively. Notably, there are information gaps in the evidence base for some pathways in several subregions (Fig. S10).

Subregion	Savanna management	Regenerative agriculture (excl. agroforestry)	Reforestation	Reduced woodfuel harvest in forests	Peatland restoration	Grassland restoration	Coastal wetland restoration	Climate-smart forestry	Avoided peatland conversion	Avoided grassland conversion	Avoided forest conversion	Avoided coastal wetland conversion	Agroforestry	Subregion Total	Subregion Share	Regional share
Northern America	43	55	135		38	64	40	105	53	80	64	11	42	730	5%	35%
Central America	127	218	428			26	22	57	1		259	49	340	1527	10%	
South America	278	122	862	3	100	214	5	9	44	39	568	5	527	2776	18%	
Caribbean	6	39	83			39	23	39			39	2	88	358	2%	
Western Asia	15		14				8					8	15	60	0%	13%
Central Asia			4										8	12	0%	
Eastern Asia	2	80	277			2	130	22		1	28	88	7	637	4%	
Southern Asia		21	137	2		34	6	32			105	6	110	453	3%	
South-Eastern Asia	7		139		48		100	22	107		170	90	205	888	6%	
Australia and New Zealand	33	14	59			14	10	5		36	50	23	4	248	2%	2%
Melanesia			12				39				12	13	12	88	1%	
Northern Africa	10	24	26				4	24			30	4	10	132	1%	48%
Western Africa	220	478	822	1		136	25	331	18	56	186	18	630	2921	19%	
Eastern Africa	152	530	1084	2		195	101	337	54	14	419	103	839	3830	25%	
Central Africa	54		116					16	88		74	54	117	519	3%	
Southern Africa	3		3			20	1						2	29	0%	
Northern Europe	6		40		8	8	4	14		1			11	92	1%	2%
Western Europe	4		37			3		4		2	5		31	86	1%	
Eastern Europe	12		9					1			7		12	41	0%	
Southern Europe	19	34	33	3		4		2		1	5		32	133	1%	

Fig. S10. Heatmap of regional and subregional constraint observation counts by pathway. Each cell shows the number of constraint-pathway-location observations for a pathway in that subregion. Grey shading indicates no data for a pathway in a subregion. Shading intensity is

proportional to a subregion-pathway combination’s share in all constraint observations (red shading) or the subregional or regional shares in all constraint observations (green shading).

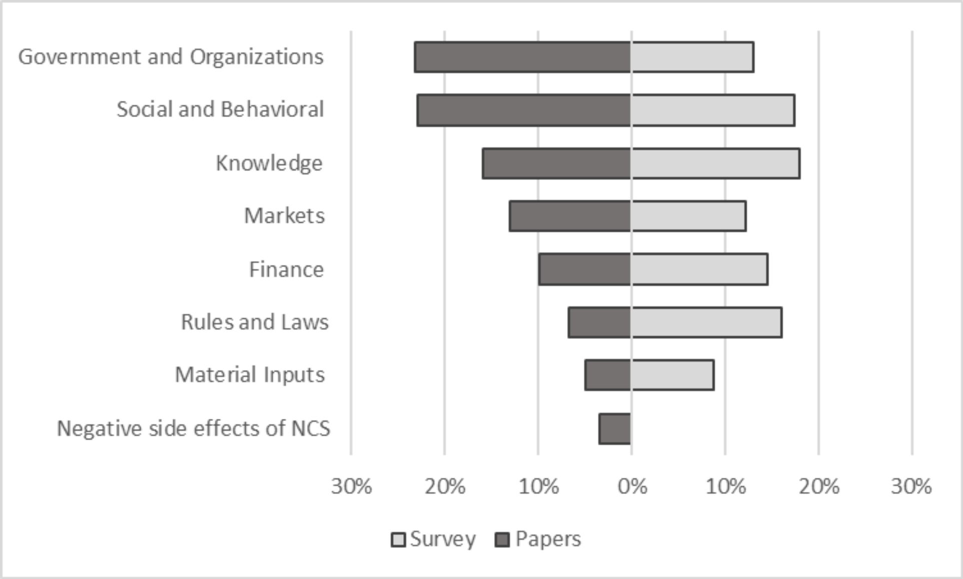

Fig. S11. Percent distribution of constraint category observations reported in the literature and survey data, respectively. Length of bars indicates the share of each constraint category in the total observation count of natural climate solutions implementation constraints reported in the survey and literature sample, respectively.

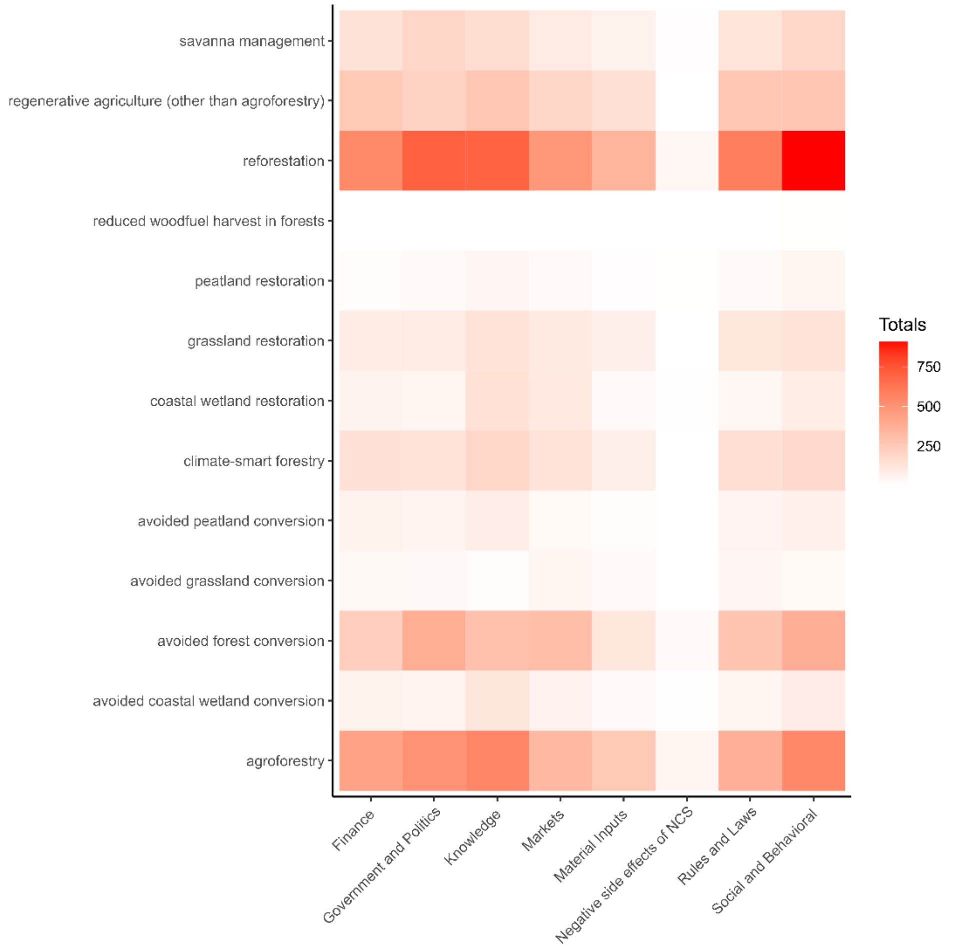

Fig. S12. Frequency counts of constraint-pathway-location observations by pathway and constraint category. Reforestation (27.7%), agroforestry (19.6%), avoided forest conversion (13.0%) and regenerative agriculture (other than agroforestry; 10.4%) are the pathways with the largest numbers of constraint observations. Reforestation also accounts for the most observations in all constraint categories except Negative Side Effects, contributing 25%-31% of the total frequency counts in each category, followed by agroforestry, which accounts for 34% of observations of Negative Side Effects and 17%-21% of observations in the other constraint categories.

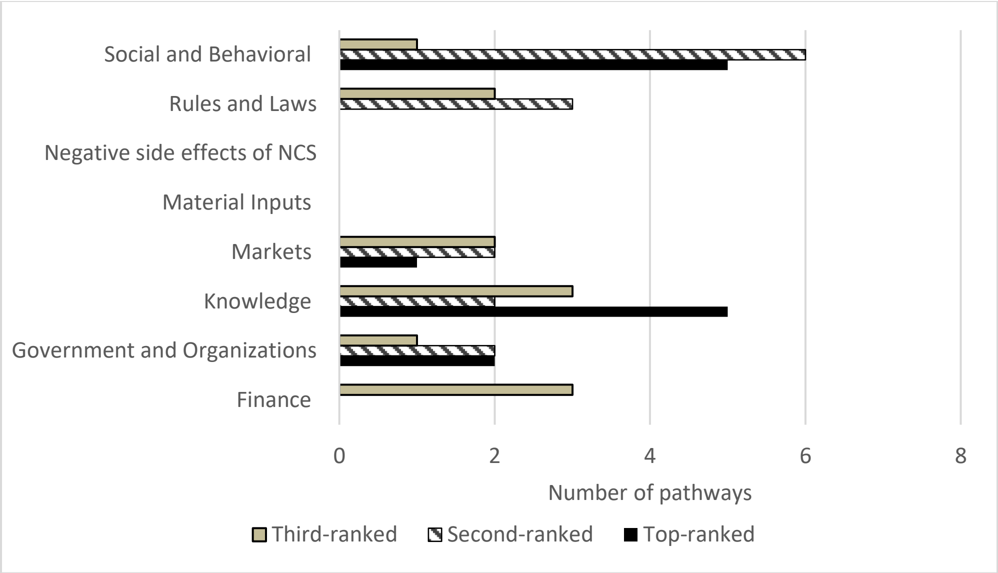

Fig. S13. Number of times each constraint category was the top, second or third-highest-ranking category for an NCS pathway. Social and behavioral and Knowledge constraints most frequently were the most-reported constraint categories for a pathway (five times each) and most frequently also were the either most, second-most, or third-most-reported constraint categories for a pathway (12 and ten times, respectively).

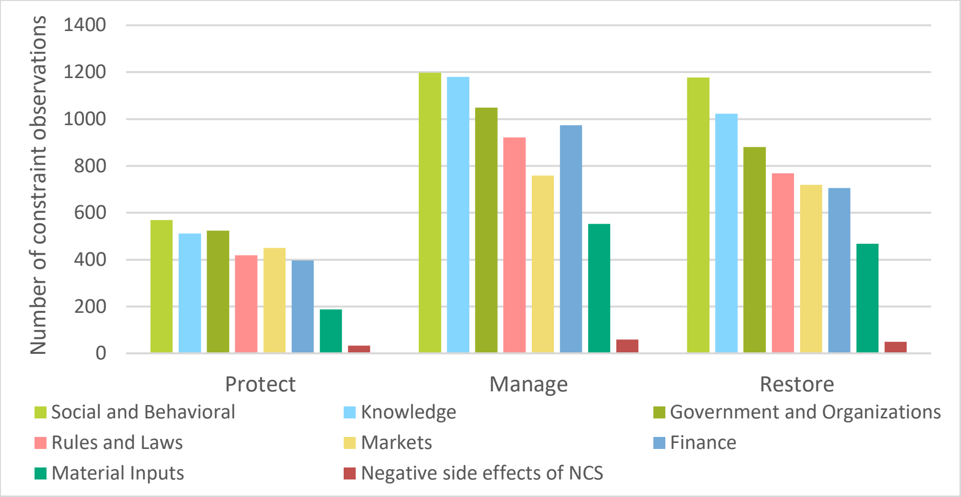

Fig. S14. Ranking of constraint categories by NCS strategy (Protect, Manage, Restore). While Knowledge is the most frequently top-ranking constraint category at the individual pathway level and across NCS strategies and the most often top-ranking category for both improved management and protection strategies, Social-behavioral is the top-ranking constraint category for three out of four restoration pathways. Within the Knowledge category, information about how to design or begin the NCS is the top or tied top individual constraint for half of the restoration pathways and half of the protection pathways, but not for any improved management pathway, although it is the second-ranking constraint for climate-smart forestry. Government and Organizations is the top-ranking constraint category for one improved management and protection pathway each. However, while Government and Organizations is a top constraint category for only two pathways, lack of policy coordination or implementation capacity is the top individual constraint for all improved management pathways if the small-sample (n=5 papers or survey responses) reduced woodfuel harvest in forests pathway is excluded. It also is the top or tied top constraint for three out of four restoration pathways. Uncertain, or lack of, enforcement of environmental laws, also in the Government and Organizations category, is the second-highest ranking individual constraint for two of the four protection pathways, underscoring the important role of lack of enforcement in environmental conservation [8].

Constraint category

Pathway-level z-score aggregation

Sub-region	Finance	Government and organizations	Knowledge	Markets	Material Inputs	Negative side effects of NCS	Rules and Laws	Social and Behavioral
Northern America	-2.82	-3.76	10.39	6.61	-9.03	-5.67	-4.66	8.93
Central America	-4.50	2.41	9.51	-3.81	-7.43		-3.95	7.78
South America	-7.60	7.35	3.38	2.34	-10.07	-3.65	0.15	8.10
Caribbean	-3.75	-6.33	8.43	-1.23	-5.43		0.79	7.52
Western Asia		-2.26	0.47	1.97				-0.18
Central Asia		0.73	-1.10		-1.10	0.73		0.73
Eastern Asia	-0.04	-1.78	6.70	0.18	-4.34	-1.12	-3.25	3.65
Southern Asia	-5.87	-1.60	5.03	3.73	-3.76	-1.00	2.07	1.40
South-Eastern Asia	1.03	0.95	3.44	2.04	-3.43	-6.48	-3.37	5.82
Australia and New Zealand	3.50	-1.50	-5.33	5.63	-3.17	2.61	-2.06	0.32
Melanesia	-0.58	-0.27	-0.05	0.35	-0.89	-3.19	-0.89	5.52
Northern Africa	1.74	1.24	-2.92	7.31	-6.26		0.73	-1.84
Western Africa	8.00	-0.55	6.15	-7.68	-10.26	-4.04	5.82	2.55
Eastern Africa	3.45	-0.62	9.85	-6.05	-9.35	-5.53	3.16	5.09
Central Africa	2.97	5.14	5.10	-4.72	-6.51	-1.88	-1.99	1.89
Southern Africa	-0.89	0.90	0.71	-0.18	-1.25		-0.72	1.43
Northern Europe	-0.67	-2.67	-1.40	1.18	2.82	-1.37	0.30	1.83
Western Europe	-0.40	0.32	0.63	-0.17	-0.03	-1.90	-1.17	2.72
Eastern Europe	-1.40	2.99	1.73	-0.35	-1.73	1.73	-3.13	0.15
Southern Europe	-2.81	-1.83	2.11	1.66	-0.31		-2.28	3.46

Raw constraint category counts aggregation

Sub-region	Finance	Government and organizations	Knowledge	Markets	Material Inputs	Negative side effects of NCS	Rules and Laws	Social and Behavioral	Observations by sub-region
Northern America	83	74	160	123	57	7	70	156	730
Central America	150	309	286	167	99		159	357	1527
South America	254	653	414	386	162	4	365	538	2776
Caribbean	45	35	89	38	34		57	72	370
Western Asia		6	20	24				10	60
Central Asia		2	1		1	2		6	12
Eastern Asia	75	81	147	96	39	1	37	161	637
Southern Asia	40	54	73	69	25	24	79	89	453
South-Eastern Asia	120	125	158	160	60	14	91	160	888
Australia and New Zealand	42	25	25	45	19	26	25	41	248
Melanesia	3	4	24	6	2	15	2	32	88
Northern Africa	18	17	14	47	4		16	16	132
Western Africa	526	390	447	269	294	8	531	456	2921
Eastern Africa	587	531	683	399	350	20	586	674	3830
Central Africa	95	99	106	27	38	1	65	88	519
Southern Africa	4	5	4	3	1		2	10	29
Northern Europe	13	8	10	22	9	8	6	16	92
Western Europe	11	13	16	9	5	3	1	28	86
Eastern Europe	2	7	8	4	4	8	6	2	41
Southern Europe	8	14	30	34	4		11	32	133

X Highest-ranking constraint category X Second-highest-ranking constraint category X Third-highest-ranking constraint category

Number of top-3 rankings of each constraint category across subregions

	Finance	Government and organizations	Knowledge	Markets	Material Inputs	Negative side effects of NCS	Rules and Laws	Social and Behavioral
**Pathway-level z-score aggregation**								
# of top-1-rankings	1	3	6	3	1	1	0	7
# of top-2 rankings	3	5	13	4	1	2	0	13
# of top-3 rankings	5	8	16	10	1	3	3	14
**Raw constraint category counts aggregation**								
# of top-1-rankings	0	1	5	6	0	1	1	8
# of top-2 rankings	3	5	9	6	0	2	2	13
# of top-3 rankings	7	8	14	8	0	3	3	17

Fig. S15. Heatmaps showing the relative prevalence of observations by constraint category and subregion using two different aggregation approaches. Top left panel shows summed pathway-level z-scores for each constraint category in each subregion. Top right panel shows total raw counts of observations for each constraint category for all pathways in each subregion. Top-three-ranking categories in each subregion are shown in bold. Top and second-ranking categories in each subregion are indicated by thick and thin borders, respectively. Each panel is color-coded at the subregion level, with darker shading indicating more frequently observed constraint categories. Grey shading indicates no data. Bottom panels show the number of times each constraint category was top, top two, or top three-ranking across all subregions, for pathway-level z-score aggregation (left) and summed raw constraint observations (right), and are color-coded by row in each panel, with darker shading indicating higher numbers of top rankings. Six of the 20 subregions have identical rankings of the top three constraint categories under both ranking approaches. A further eight have the same top three constraint categories under both approaches, but with different rankings. In the remaining six subregions, two out of the top three constraint categories are the same under both approaches (with the same or a different ranking) but there is a different third top-three constraint category.

Constraint category / *Sub-region*	Finance	Government and Organiz.	Knowledge	Markets	Material Inputs	Negative side effects of NCS	Rules and Laws	Social and Behavioral	*Subregional share*		*Regional share*
Northern America	83	74	160	123	57	7	70	156	*730*	*5%*	*35%*
Central America	150	309	286	167	99		159	357	*1527*	*10%*	
South America	254	653	414	386	162	4	365	538	*2776*	*18%*	
Caribbean	45	35	89	38	34		57	72	*370*	*2%*	
Western Asia		6	20	24				10	*60*	*0%*	*13%*
Central Asia		2	1		1	2		6	*12*	*0%*	
Eastern Asia	75	81	147	96	39	1	37	161	*637*	*4%*	
Southern Asia	40	54	73	69	25	24	79	89	*453*	*3%*	
South-Eastern Asia	120	125	158	160	60	14	91	160	*888*	*6%*	
Australia and New Zealand	42	25	25	45	19	26	25	41	*248*	*2%*	*2%*
Melanesia	3	4	24	6	2	15	2	32	*88*	*1%*	
Northern Africa	18	17	14	47	4		16	16	*132*	*1%*	*48%*
Western Africa	526	390	447	269	294	8	531	456	*2921*	*19%*	
Eastern Africa	587	531	683	399	350	20	586	674	*3830*	*25%*	
Central Africa	95	99	106	27	38	1	65	88	*519*	*3%*	
Southern Africa	4	5	4	3	1		2	10	*29*	*0%*	
Northern Europe	13	8	10	22	9	8	6	16	*92*	*1%*	*2%*
Western Europe	11	13	16	9	5	3	1	28	*86*	*1%*	
Eastern Europe	2	7	8	4	4	8	6	2	*41*	*0%*	
Southern Europe	8	14	30	34	4		11	32	*133*	*1%*	

Fig. S16. Heatmap showing number of observations in each constraint category by subregion. Each cell shows the number of constraint-pathway-location observations for a constraint category in that subregion. Color-coding identifies subregion-constraint category combinations with the highest counts overall. Grey shading indicates no data.

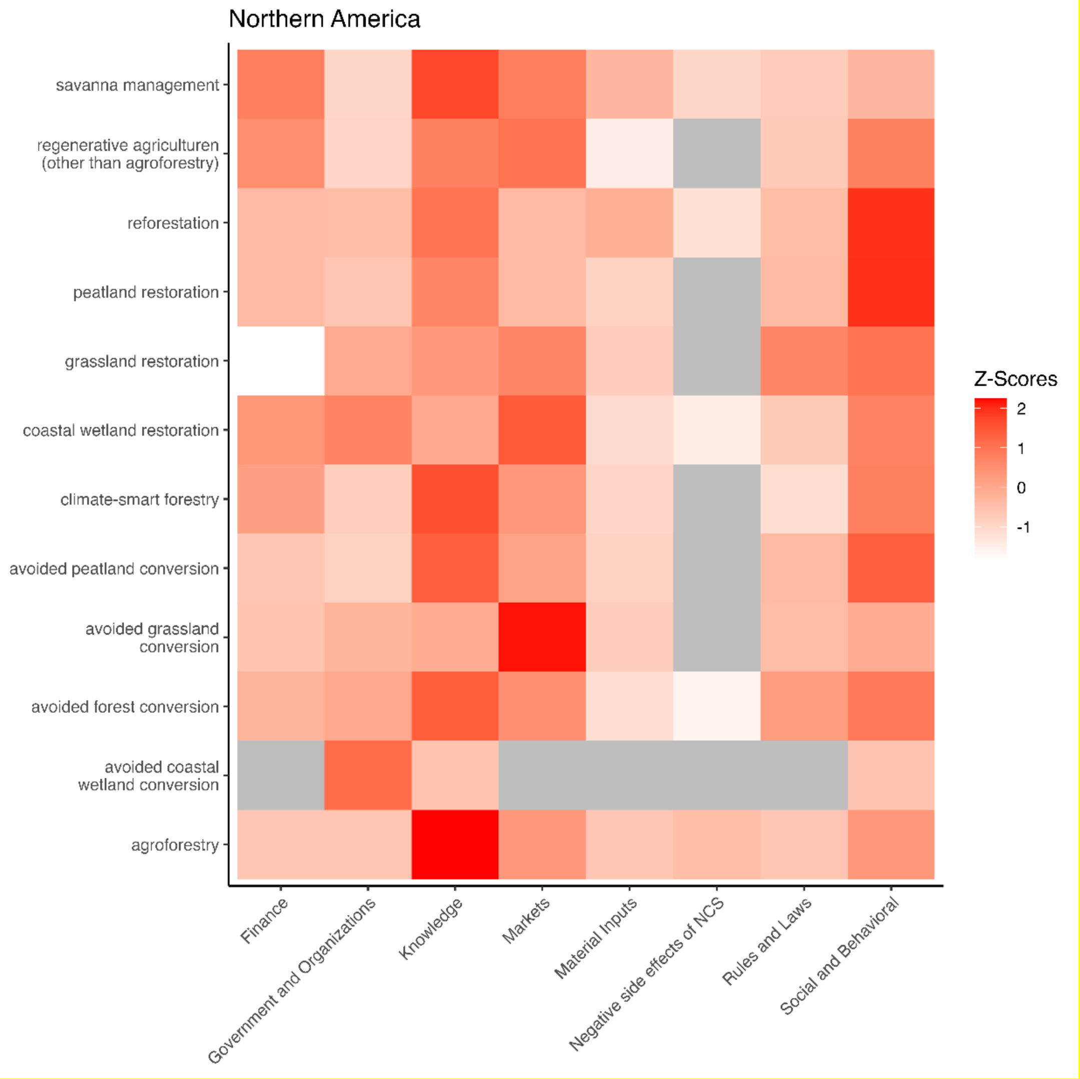

Fig. S17a. Pathway-level subregional z-score heatmaps for all 20 subregions with data. Maps indicate the frequency with which a given constraint category was observed for a given pathway in a subregion compared to the mean frequency for all constraint categories for the pathway in that subregion, expressed as the number of standard deviations from the mean. Maps are color-coded at the pathway level. Grey indicates no data. Hashing indicates undefined z-score (standard deviation = 0).

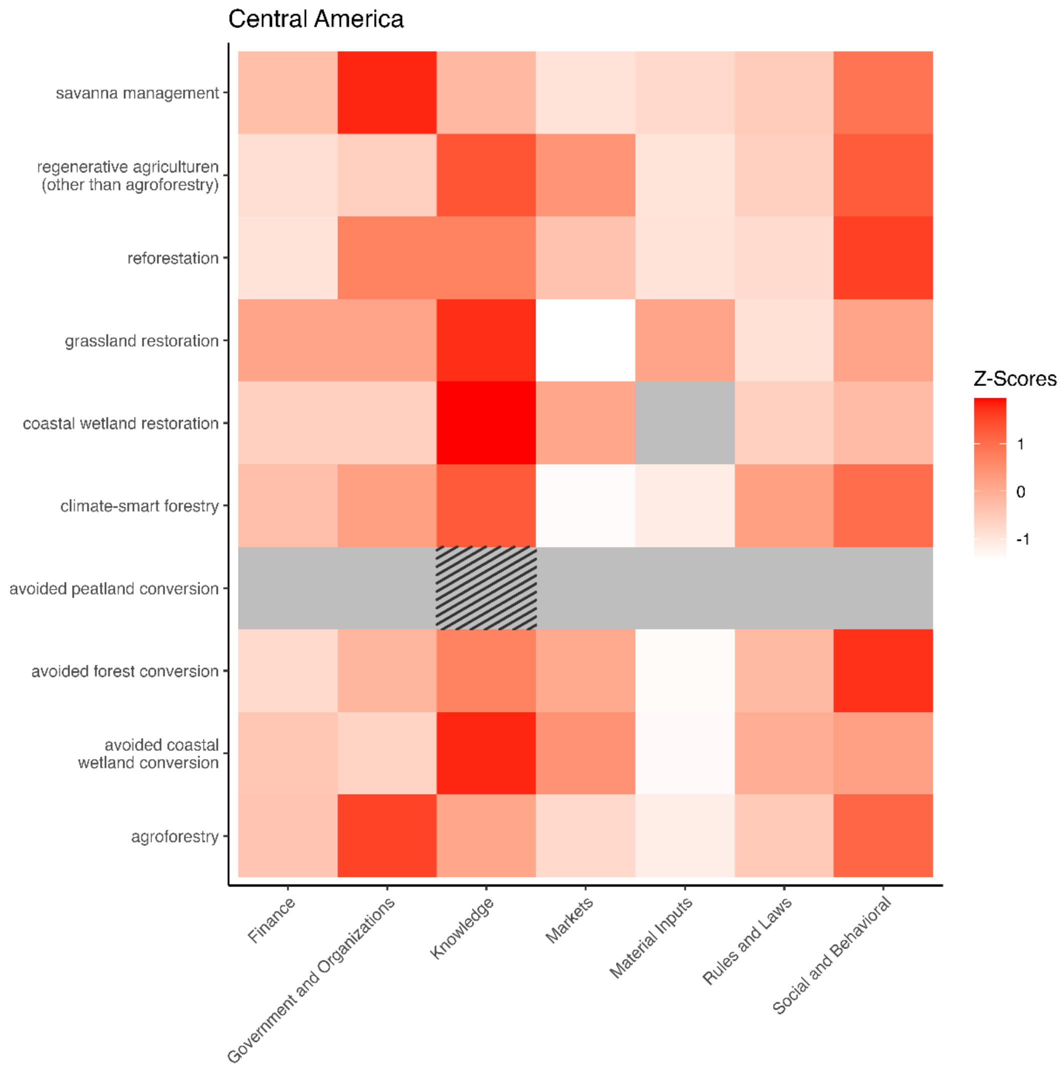

Fig. S17b. Pathway-level subregional z-score heatmaps for all 20 subregions with data. Maps indicate the frequency with which a given constraint category was observed for a given pathway in a subregion compared to the mean frequency for all constraint categories for the pathway in that subregion, expressed as the number of standard deviations from the mean. Maps are color-coded at the pathway level. Grey indicates no data. Hashing indicates undefined z-score (standard deviation = 0).

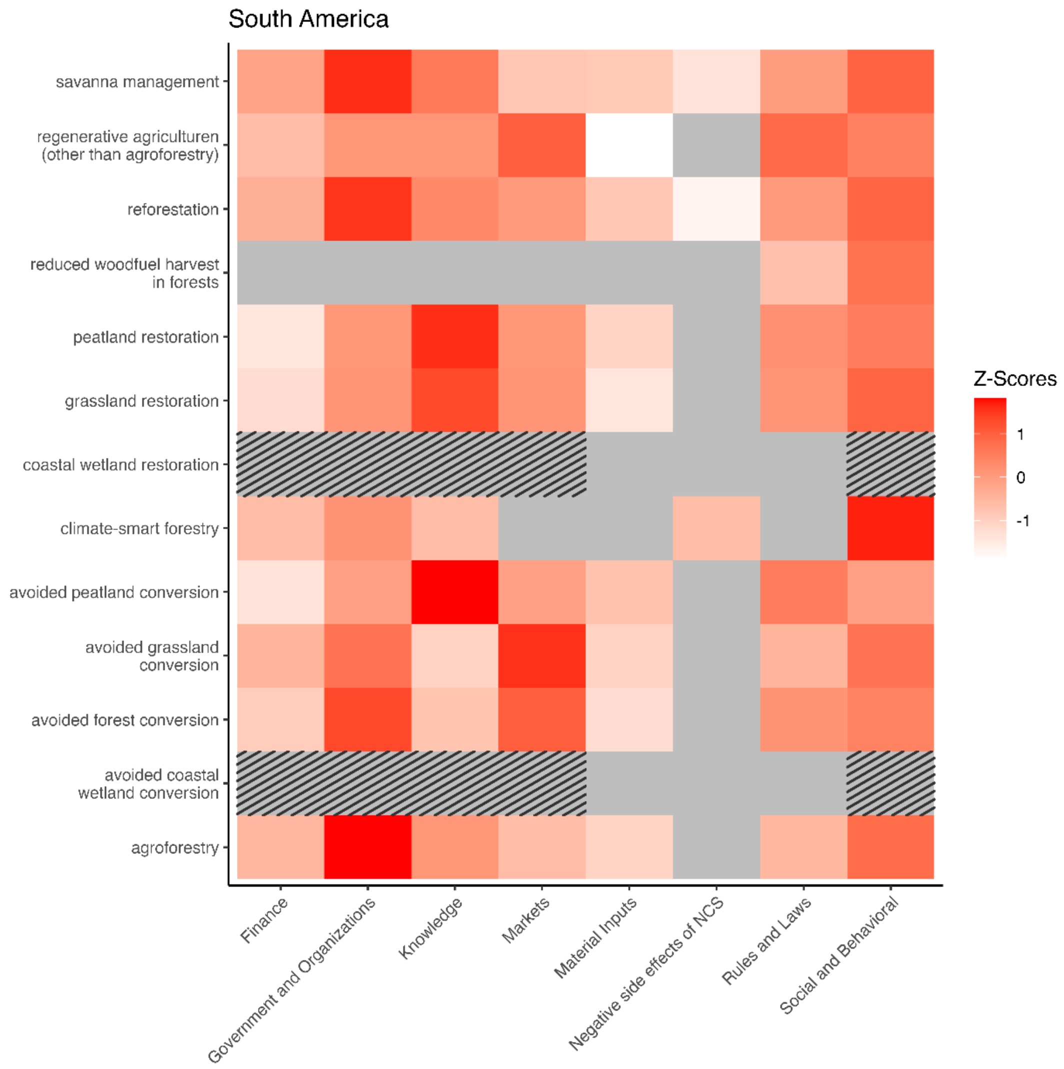

Fig. S17c. Pathway-level subregional z-score heatmaps for all 20 subregions with data. Maps indicate the frequency with which a given constraint category was observed for a given pathway in a subregion compared to the mean frequency for all constraint categories for the pathway in that subregion, expressed as the number of standard deviations from the mean. Maps are color-coded at the pathway level. Grey indicates no data. Hashing indicates undefined z-score (standard deviation = 0).

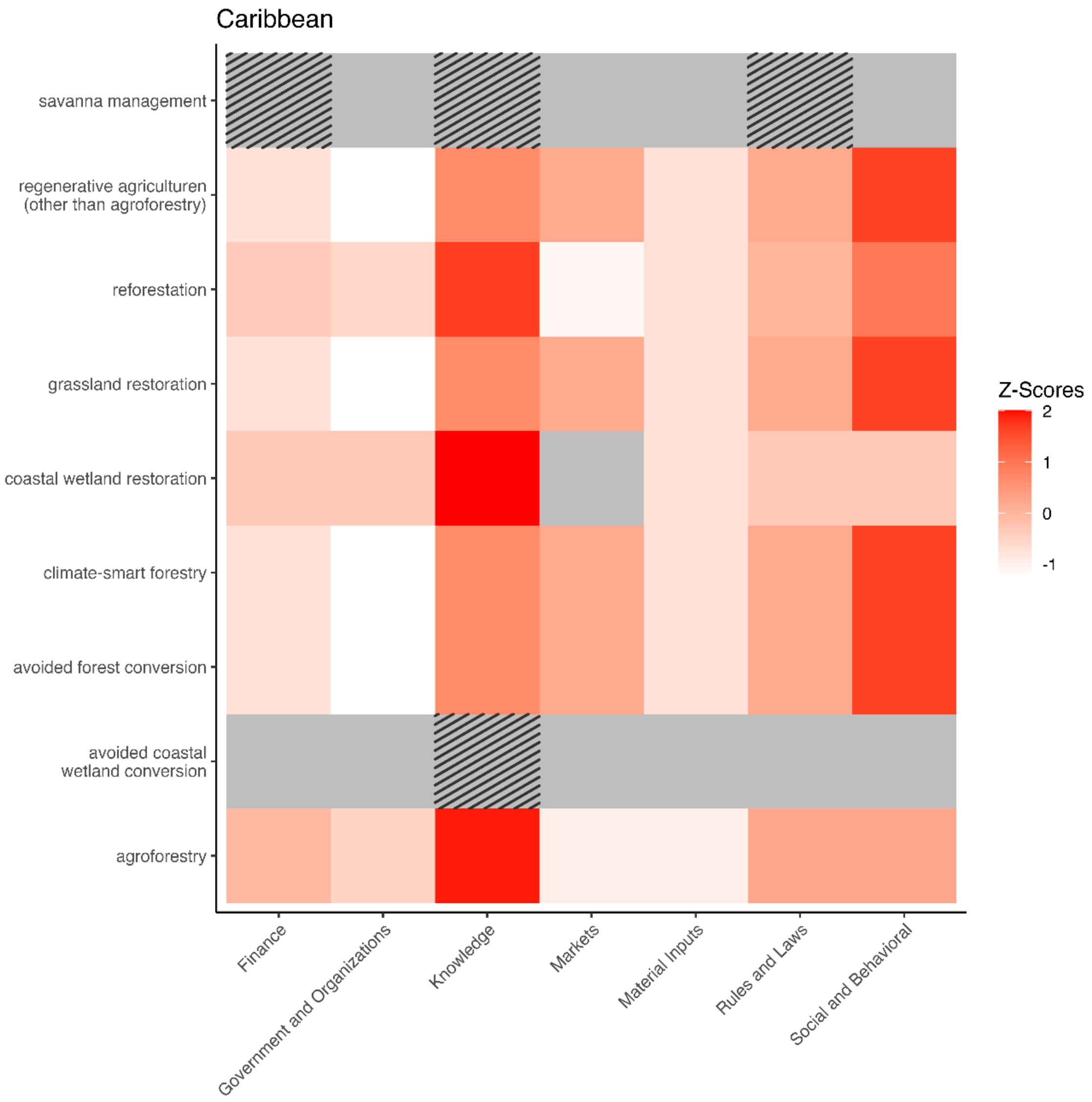

Fig. S17d. Pathway-level subregional z-score heatmaps for all 20 subregions with data. Maps indicate the frequency with which a given constraint category was observed for a given pathway in a subregion compared to the mean frequency for all constraint categories for the pathway in that subregion, expressed as the number of standard deviations from the mean. Maps are color-coded at the pathway level. Grey indicates no data. Hashing indicates undefined z-score (standard deviation = 0).

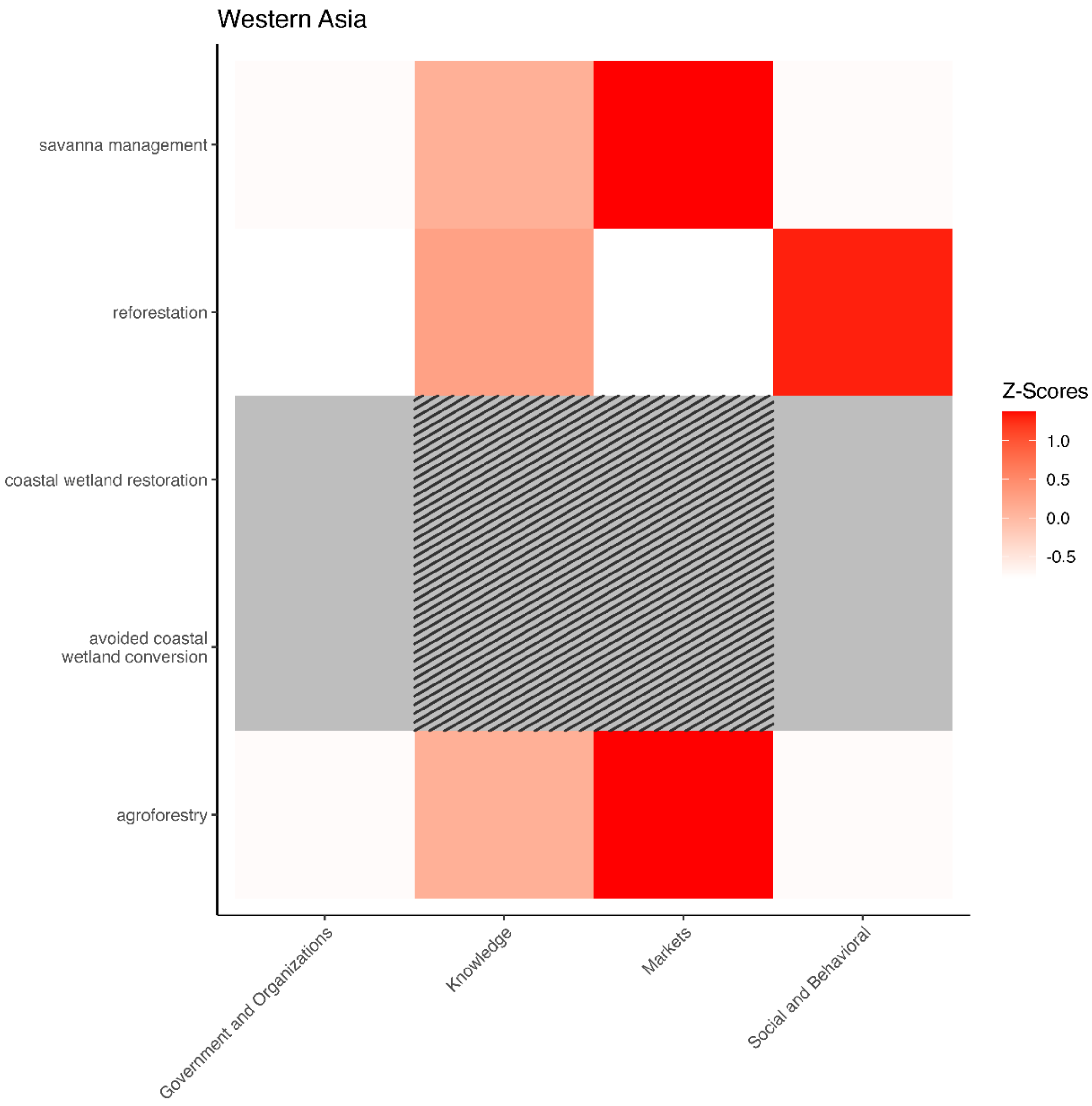

Fig. S17e. Pathway-level subregional z-score heatmaps for all 20 subregions with data. Maps indicate the frequency with which a given constraint category was observed for a given pathway in a subregion compared to the mean frequency for all constraint categories for the pathway in that subregion, expressed as the number of standard deviations from the mean. Maps are color-coded at the pathway level. Grey indicates no data. Hashing indicates undefined z-score (standard deviation = 0).

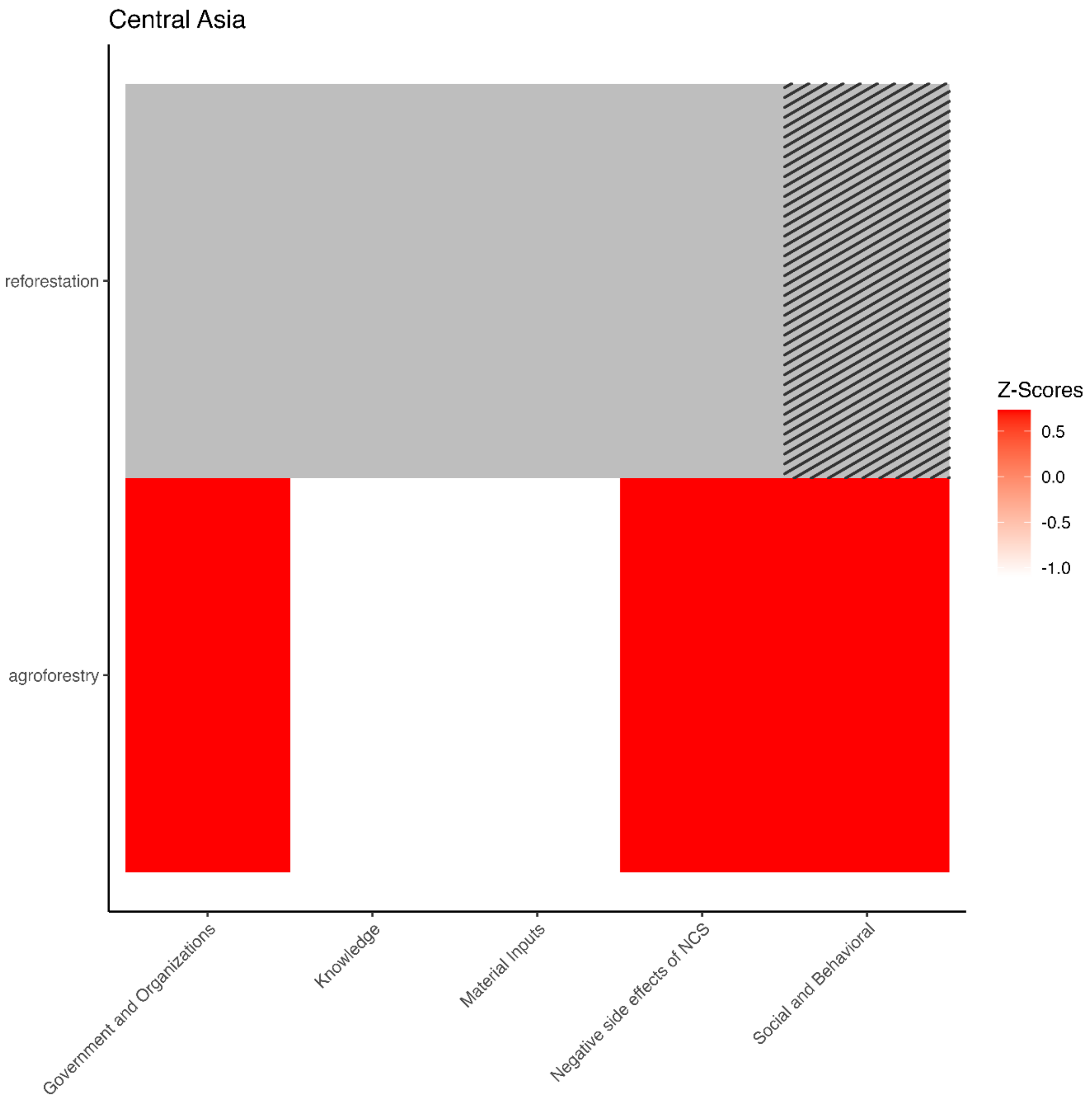

Fig. S17f. Pathway-level subregional z-score heatmaps for all 20 subregions with data. Maps indicate the frequency with which a given constraint category was observed for a given pathway in a subregion compared to the mean frequency for all constraint categories for the pathway in that subregion, expressed as the number of standard deviations from the mean. Maps are color-coded at the pathway level. Grey indicates no data. Hashing indicates undefined z-score (standard deviation = 0).

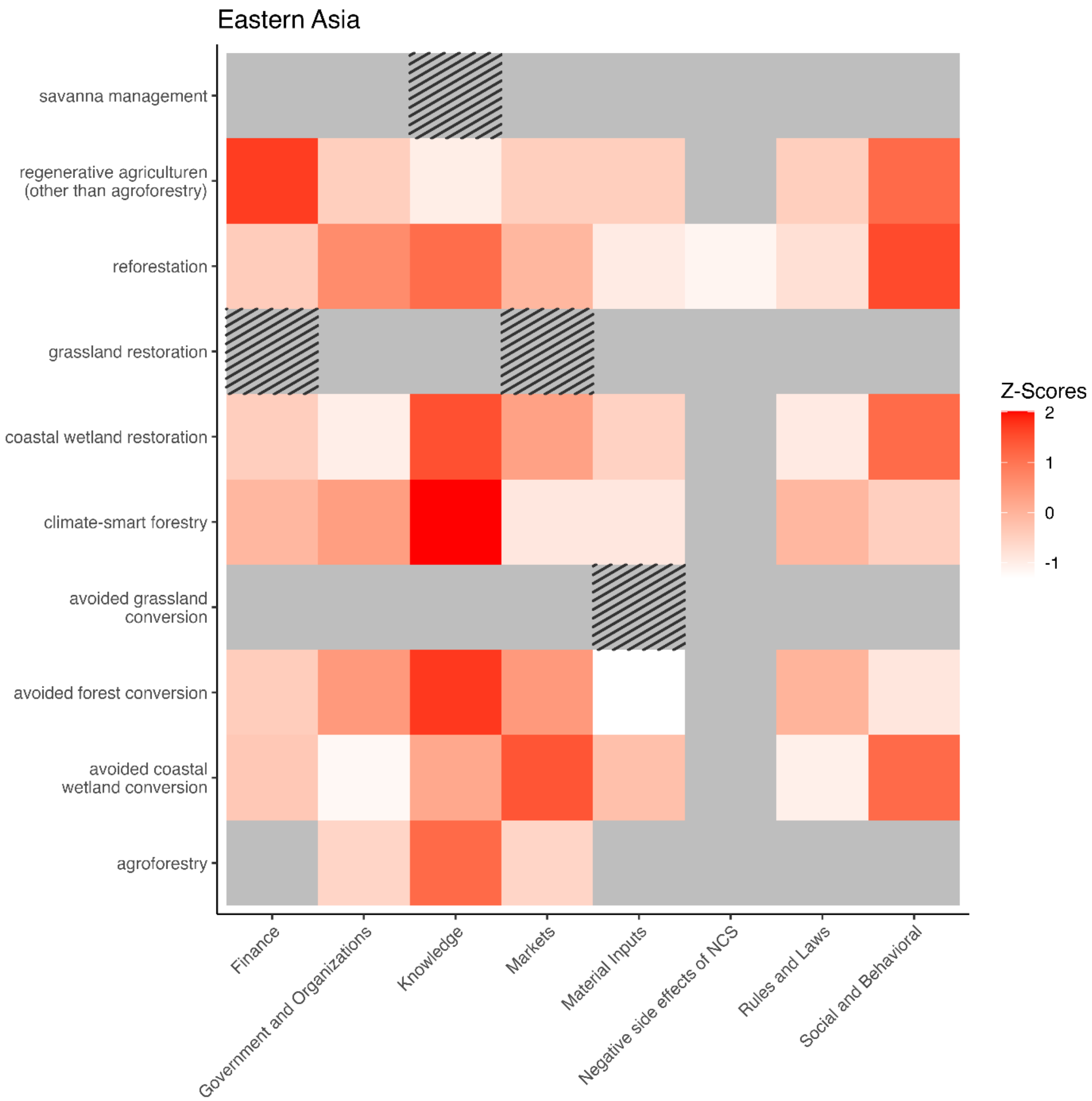

Fig. S17g. Pathway-level subregional z-score heatmaps for all 20 subregions with data. Maps indicate the frequency with which a given constraint category was observed for a given pathway in a subregion compared to the mean frequency for all constraint categories for the pathway in that subregion, expressed as the number of standard deviations from the mean. Maps are color-coded at the pathway level. Grey indicates no data. Hashing indicates undefined z-score (standard deviation = 0).

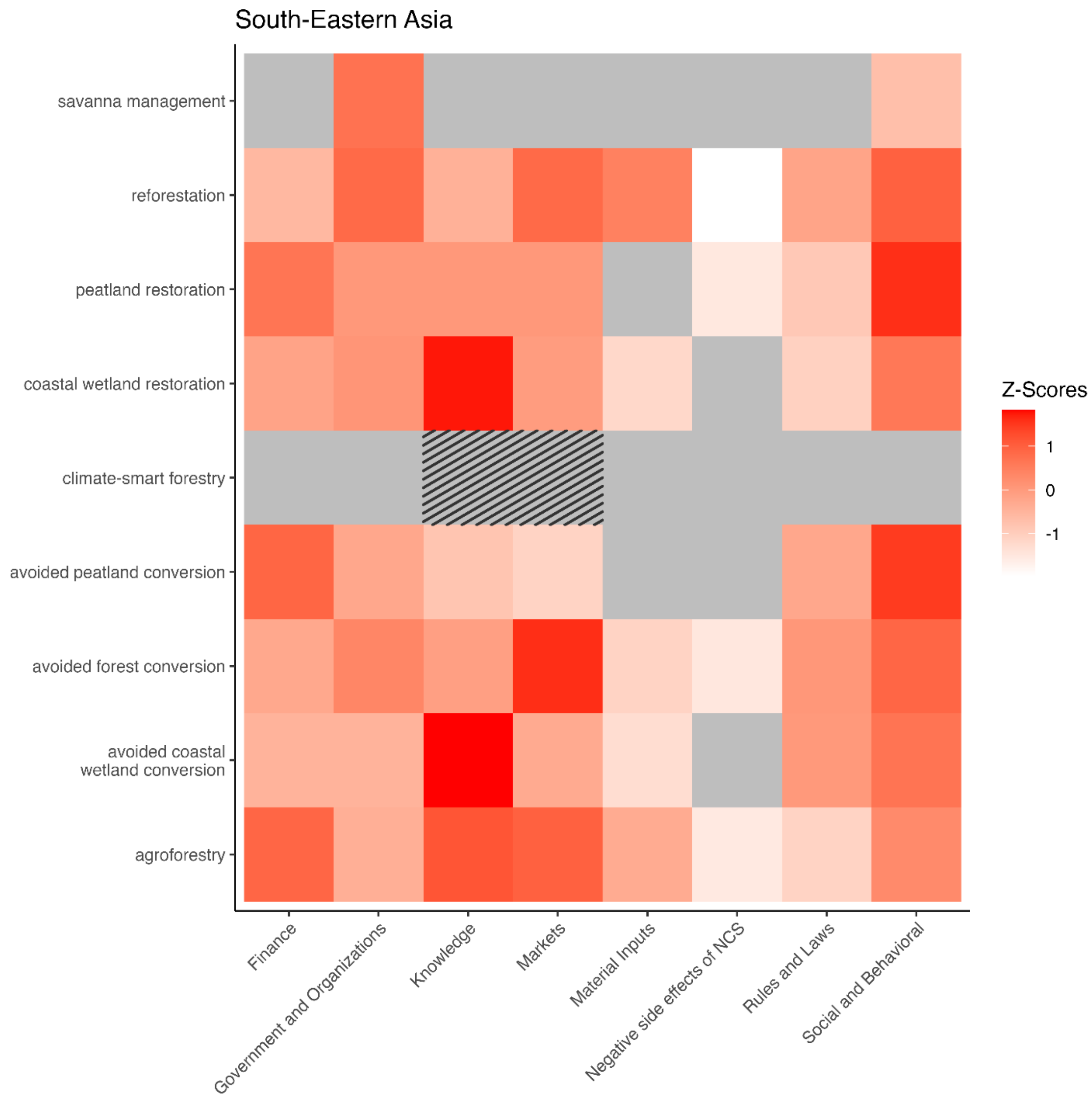

Fig. S17h. Pathway-level subregional z-score heatmaps for all 20 subregions with data. Maps indicate the frequency with which a given constraint category was observed for a given pathway in a subregion compared to the mean frequency for all constraint categories for the pathway in that subregion, expressed as the number of standard deviations from the mean. Maps are color-coded at the pathway level. Grey indicates no data. Hashing indicates undefined z-score (standard deviation = 0).

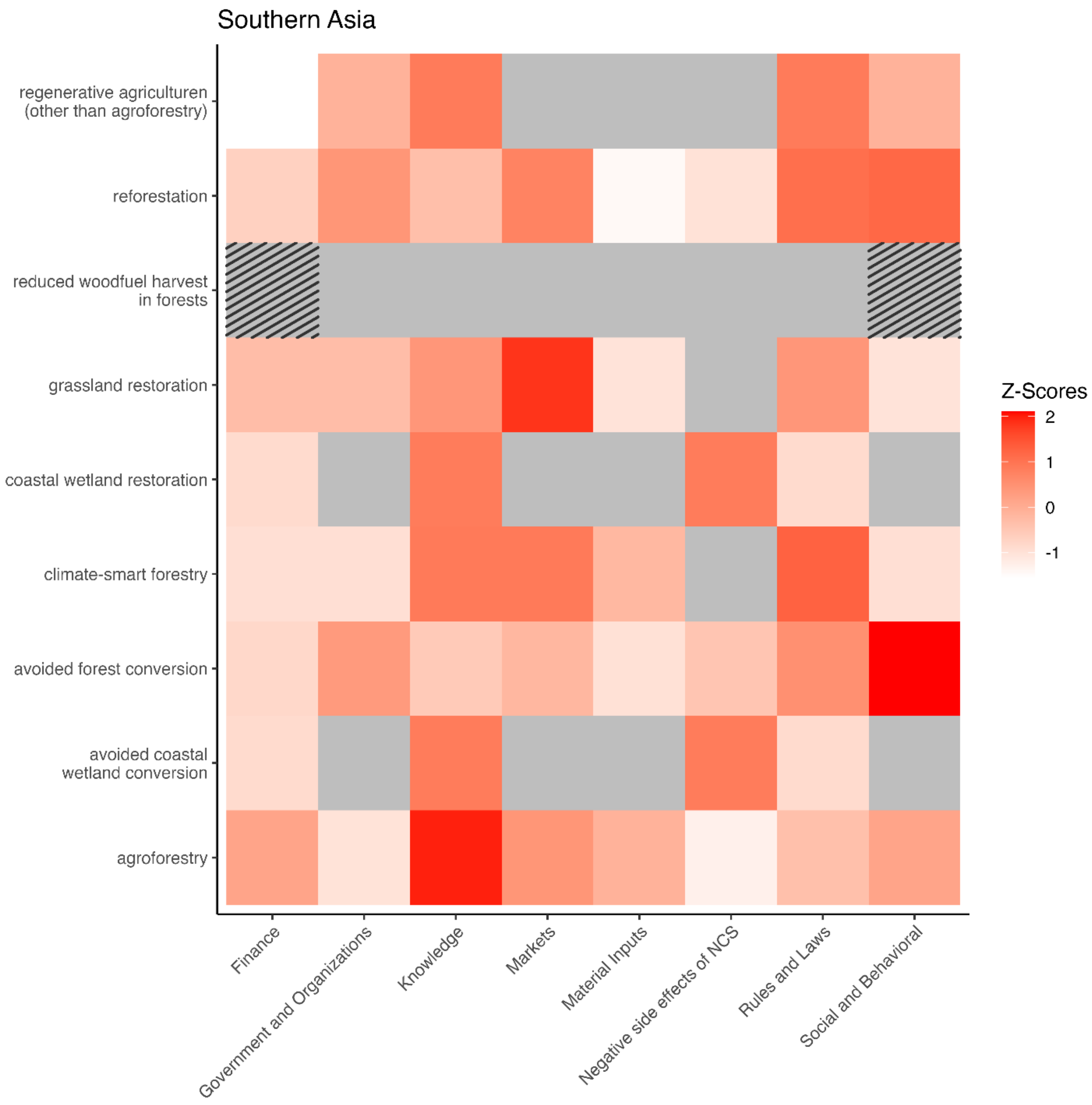

Fig. S17i. Pathway-level subregional z-score heatmaps for all 20 subregions with data. Maps indicate the frequency with which a given constraint category was observed for a given pathway in a subregion compared to the mean frequency for all constraint categories for the pathway in that subregion, expressed as the number of standard deviations from the mean. Maps are color-coded at the pathway level. Grey indicates no data. Hashing indicates undefined z-score (standard deviation = 0).

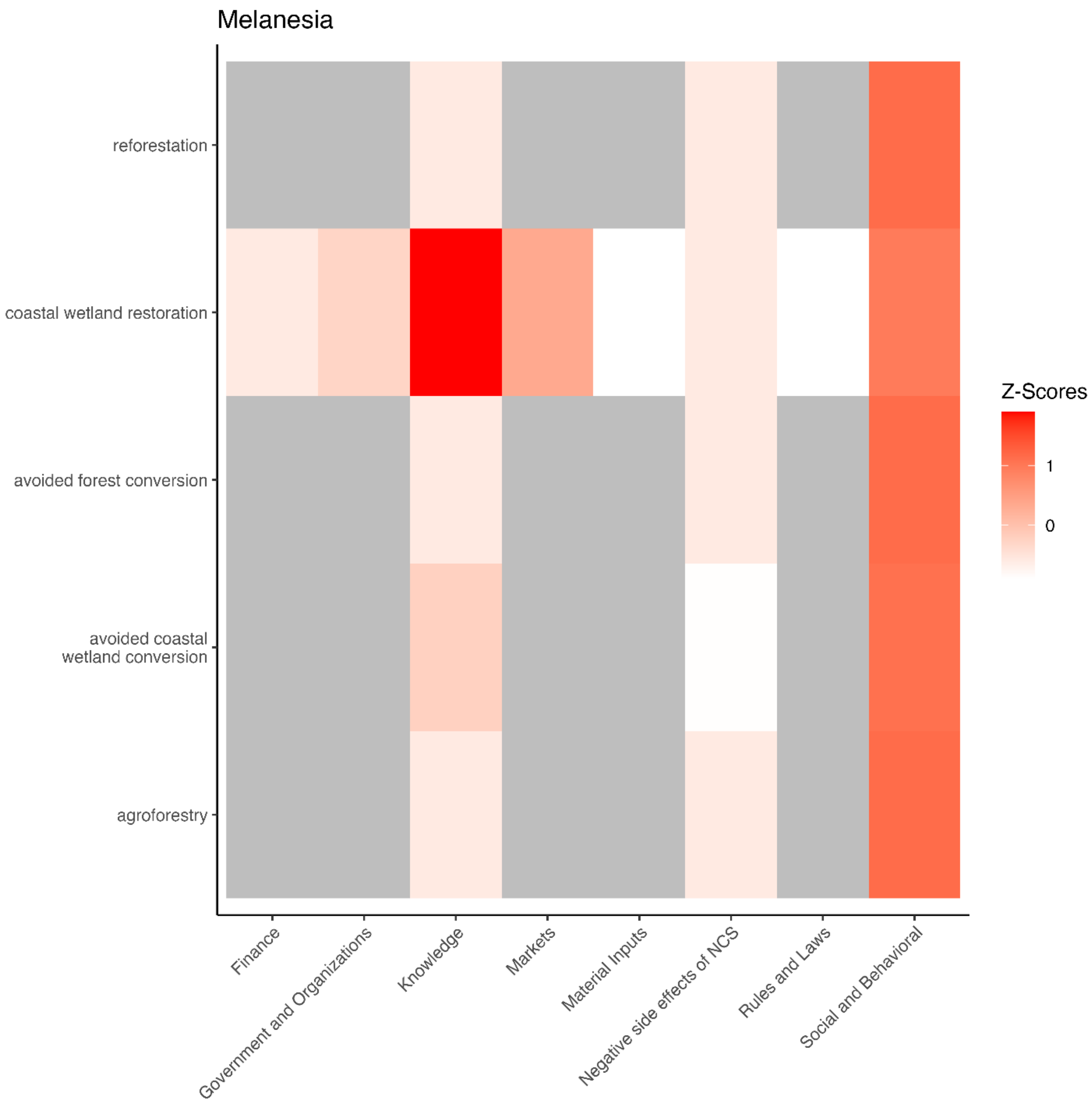

Fig. S17j. Pathway-level subregional z-score heatmaps for all 20 subregions with data. Maps indicate the frequency with which a given constraint category was observed for a given pathway in a subregion compared to the mean frequency for all constraint categories for the pathway in that subregion, expressed as the number of standard deviations from the mean. Maps are color-coded at the pathway level. Grey indicates no data. Hashing indicates undefined z-score (standard deviation = 0).

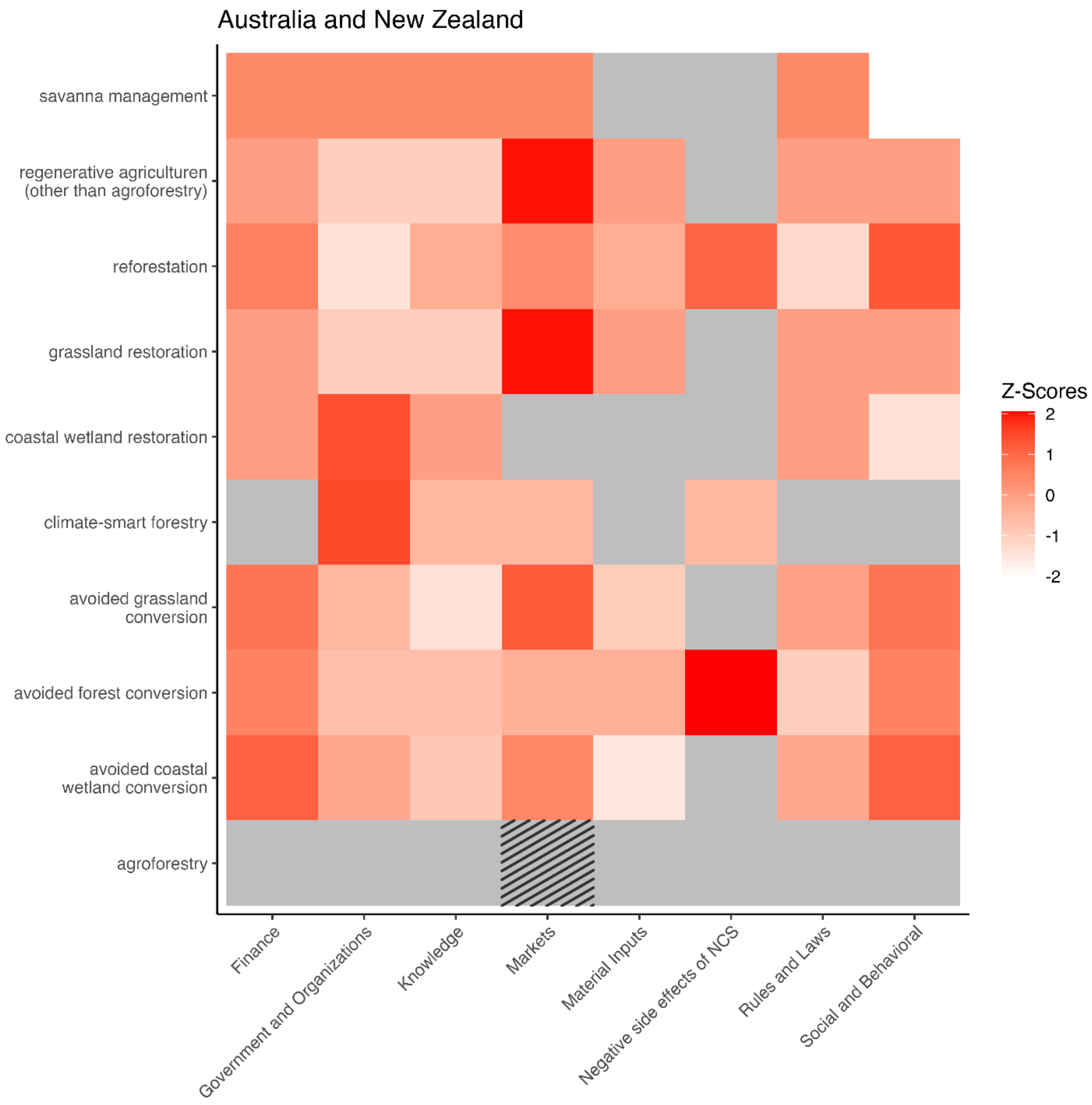

Fig. S17k. Pathway-level subregional z-score heatmaps for all 20 subregions with data. Maps indicate the frequency with which a given constraint category was observed for a given pathway in a subregion compared to the mean frequency for all constraint categories for the pathway in that subregion, expressed as the number of standard deviations from the mean. Maps are color-coded at the pathway level. Grey indicates no data. Hashing indicates undefined z-score (standard deviation = 0).

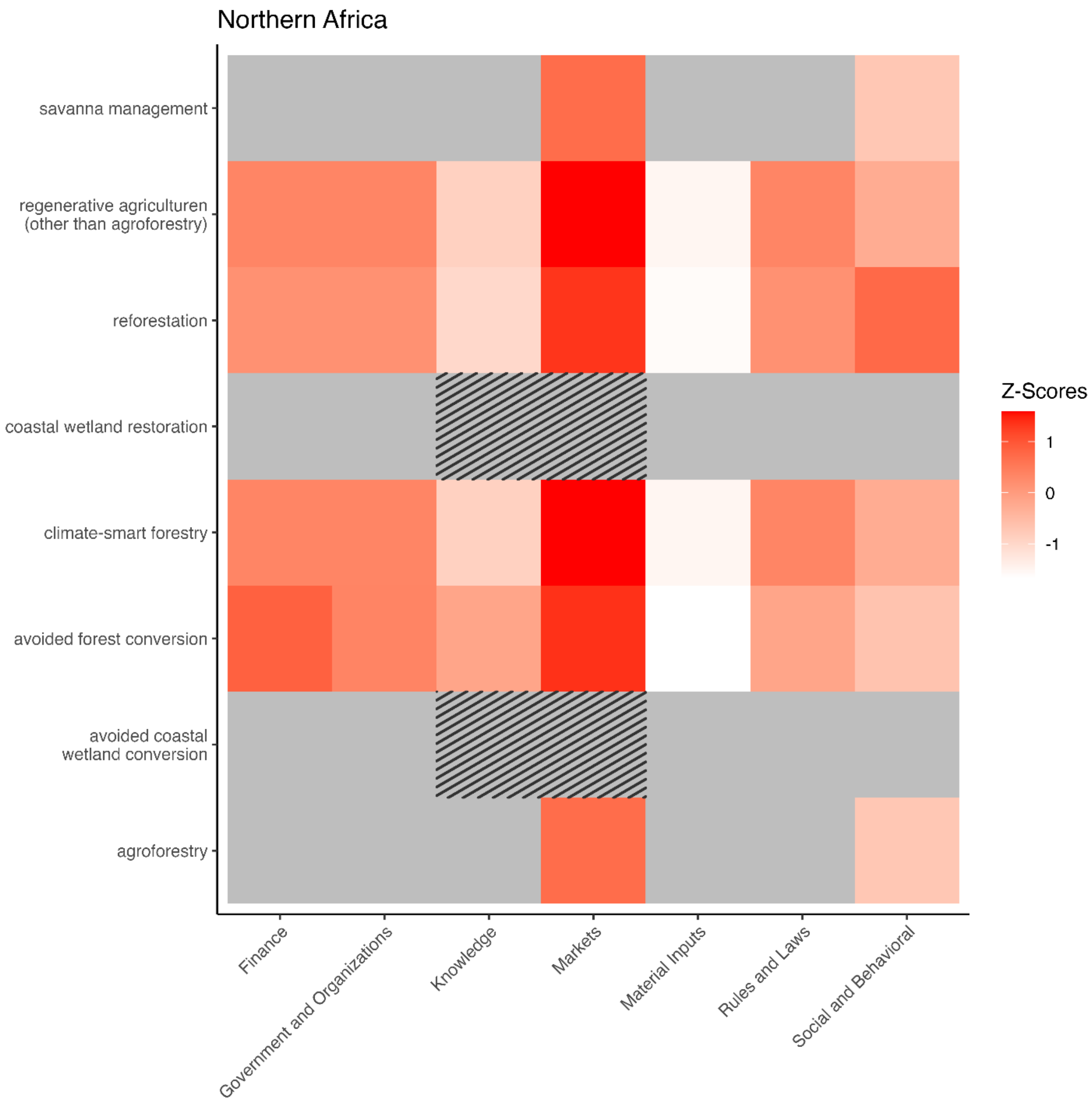

Fig. S17l. Pathway-level subregional z-score heatmaps for all 20 subregions with data. Maps indicate the frequency with which a given constraint category was observed for a given pathway in a subregion compared to the mean frequency for all constraint categories for the pathway in that subregion, expressed as the number of standard deviations from the mean. Maps are color-coded at the pathway level. Grey indicates no data. Hashing indicates undefined z-score (standard deviation = 0).

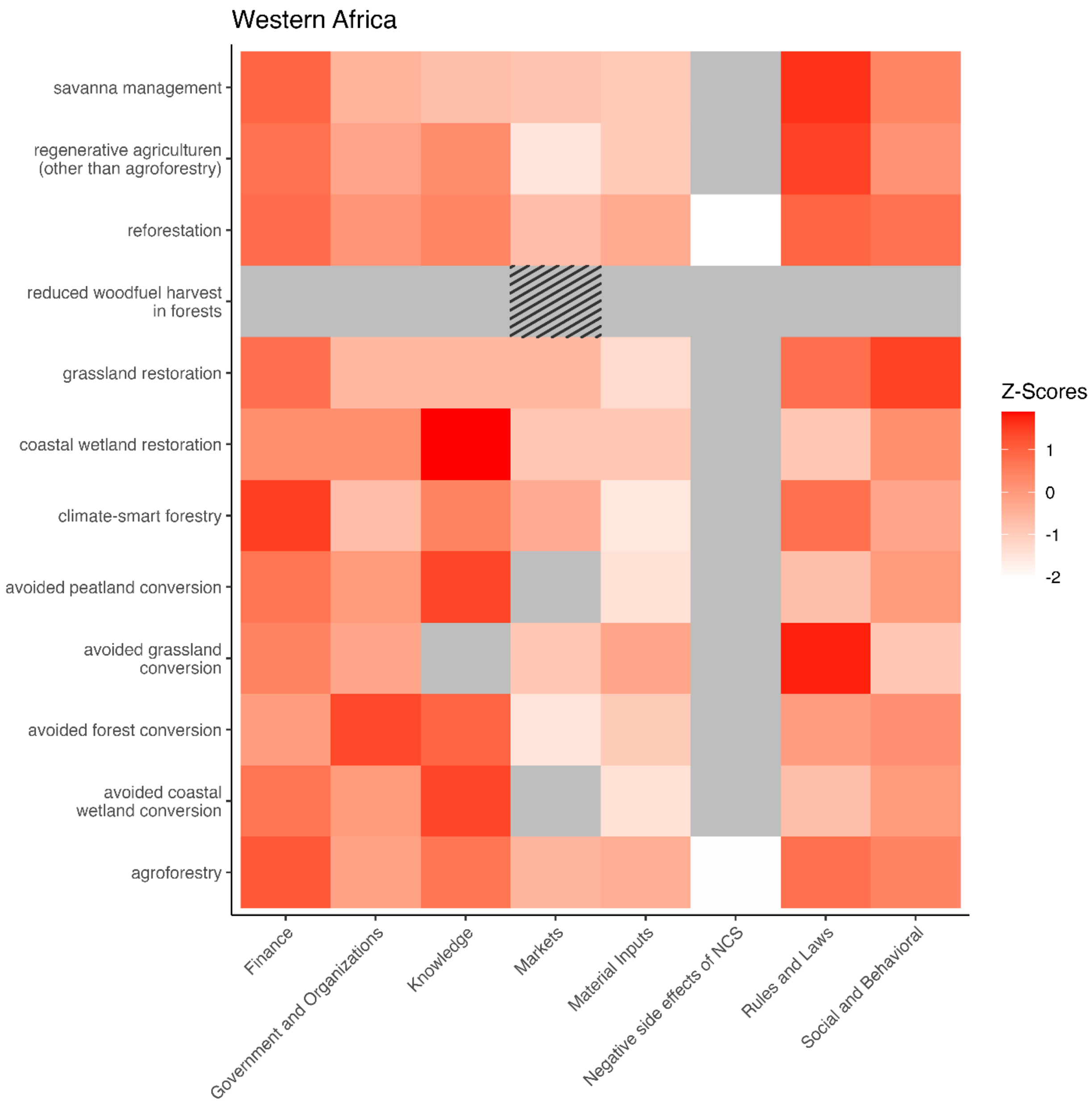

Fig. S17m. Pathway-level subregional z-score heatmaps for all 20 subregions with data. Maps indicate the frequency with which a given constraint category was observed for a given pathway in a subregion compared to the mean frequency for all constraint categories for the pathway in that subregion, expressed as the number of standard deviations from the mean. Maps are color-coded at the pathway level. Grey indicates no data. Hashing indicates undefined z-score (standard deviation = 0).

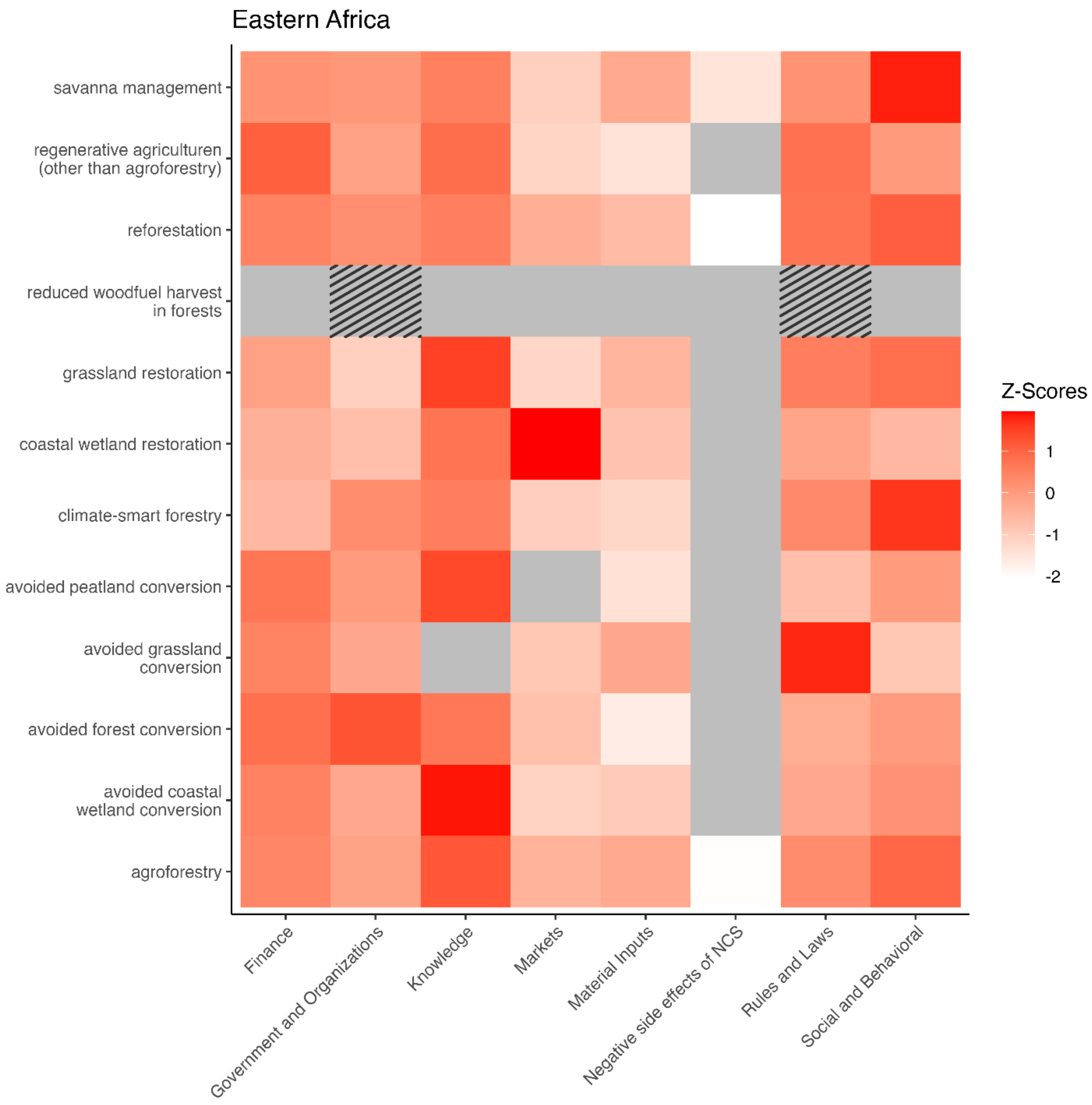

Fig. S17n. Pathway-level subregional z-score heatmaps for all 20 subregions with data. Maps indicate the frequency with which a given constraint category was observed for a given pathway in a subregion compared to the mean frequency for all constraint categories for the pathway in that subregion, expressed as the number of standard deviations from the mean. Maps are color-coded at the pathway level. Grey indicates no data. Hashing indicates undefined z-score (standard deviation = 0).

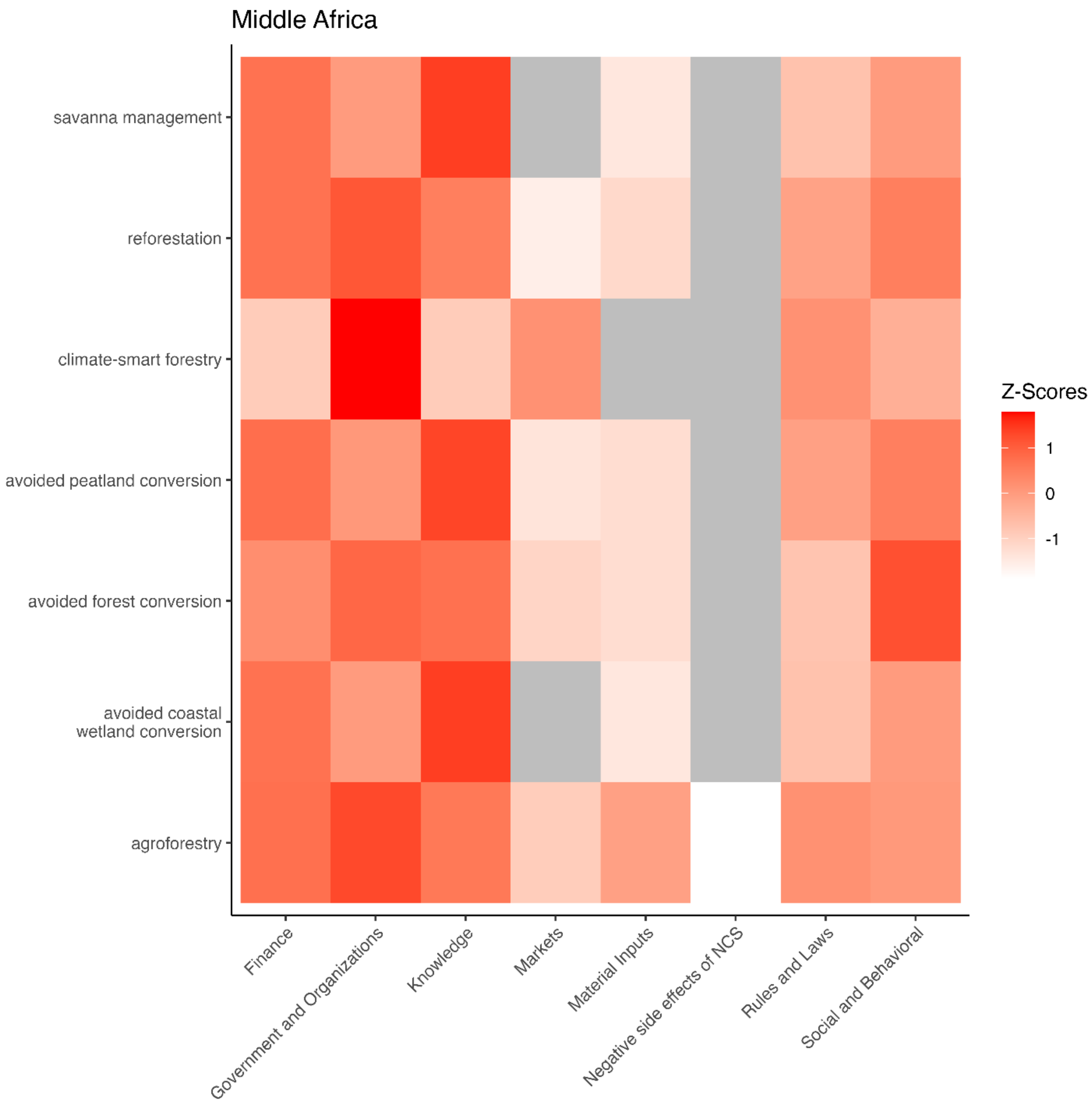

Fig. S17o. Pathway-level subregional z-score heatmaps for all 20 subregions with data. Maps indicate the frequency with which a given constraint category was observed for a given pathway in a subregion compared to the mean frequency for all constraint categories for the pathway in that subregion, expressed as the number of standard deviations from the mean. Maps are color-coded at the pathway level. Grey indicates no data. Hashing indicates undefined z-score (standard deviation = 0).

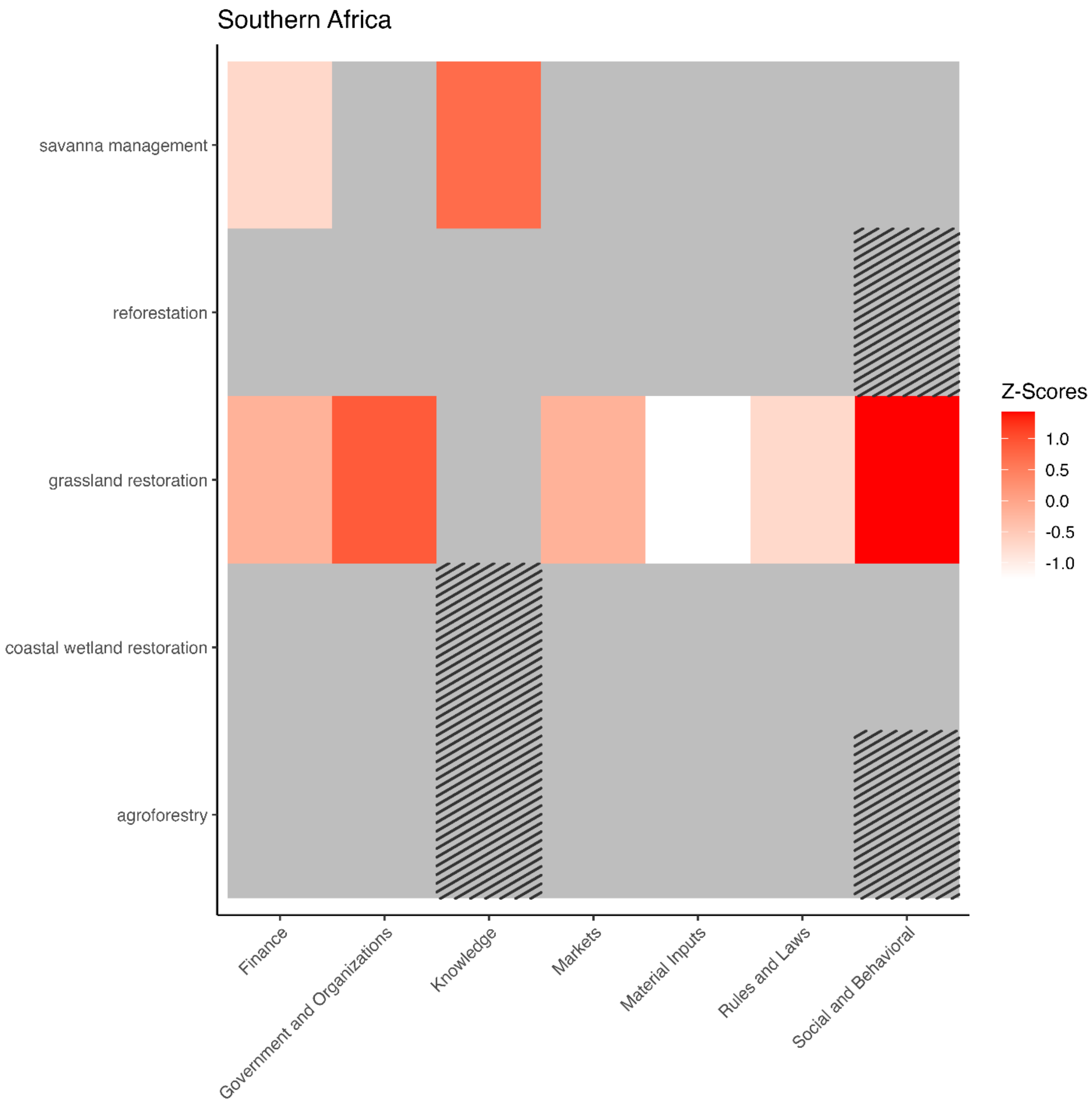

Fig. S17p. Pathway-level subregional z-score heatmaps for all 20 subregions with data. Maps indicate the frequency with which a given constraint category was observed for a given pathway in a subregion compared to the mean frequency for all constraint categories for the pathway in that subregion, expressed as the number of standard deviations from the mean. Maps are color-coded at the pathway level. Grey indicates no data. Hashing indicates undefined z-score (standard deviation = 0).

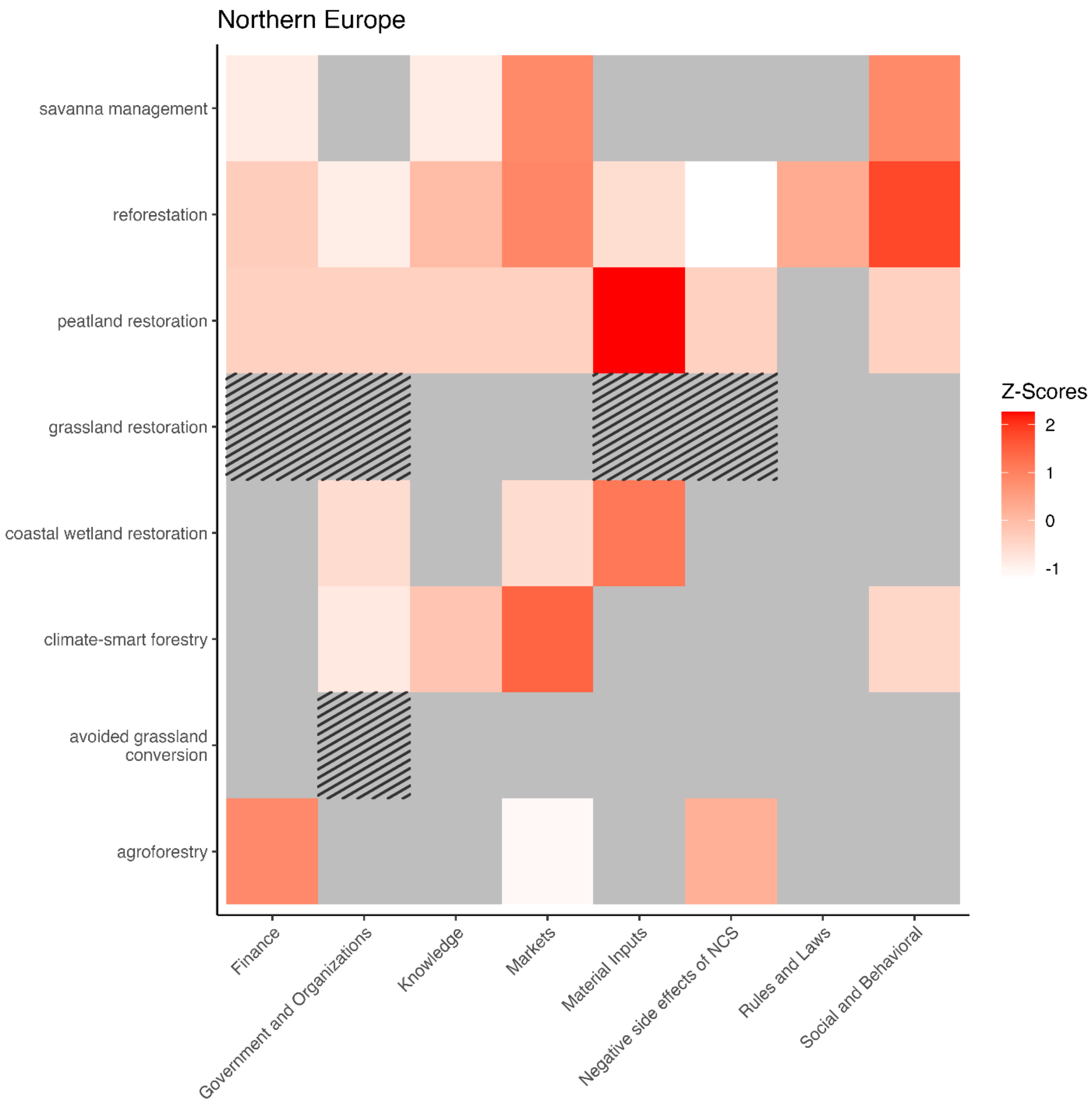

Fig. S17q. Pathway-level subregional z-score heatmaps for all 20 subregions with data. Maps indicate the frequency with which a given constraint category was observed for a given pathway in a subregion compared to the mean frequency for all constraint categories for the pathway in that subregion, expressed as the number of standard deviations from the mean. Maps are color-coded at the pathway level. Grey indicates no data. Hashing indicates undefined z-score (standard deviation = 0).

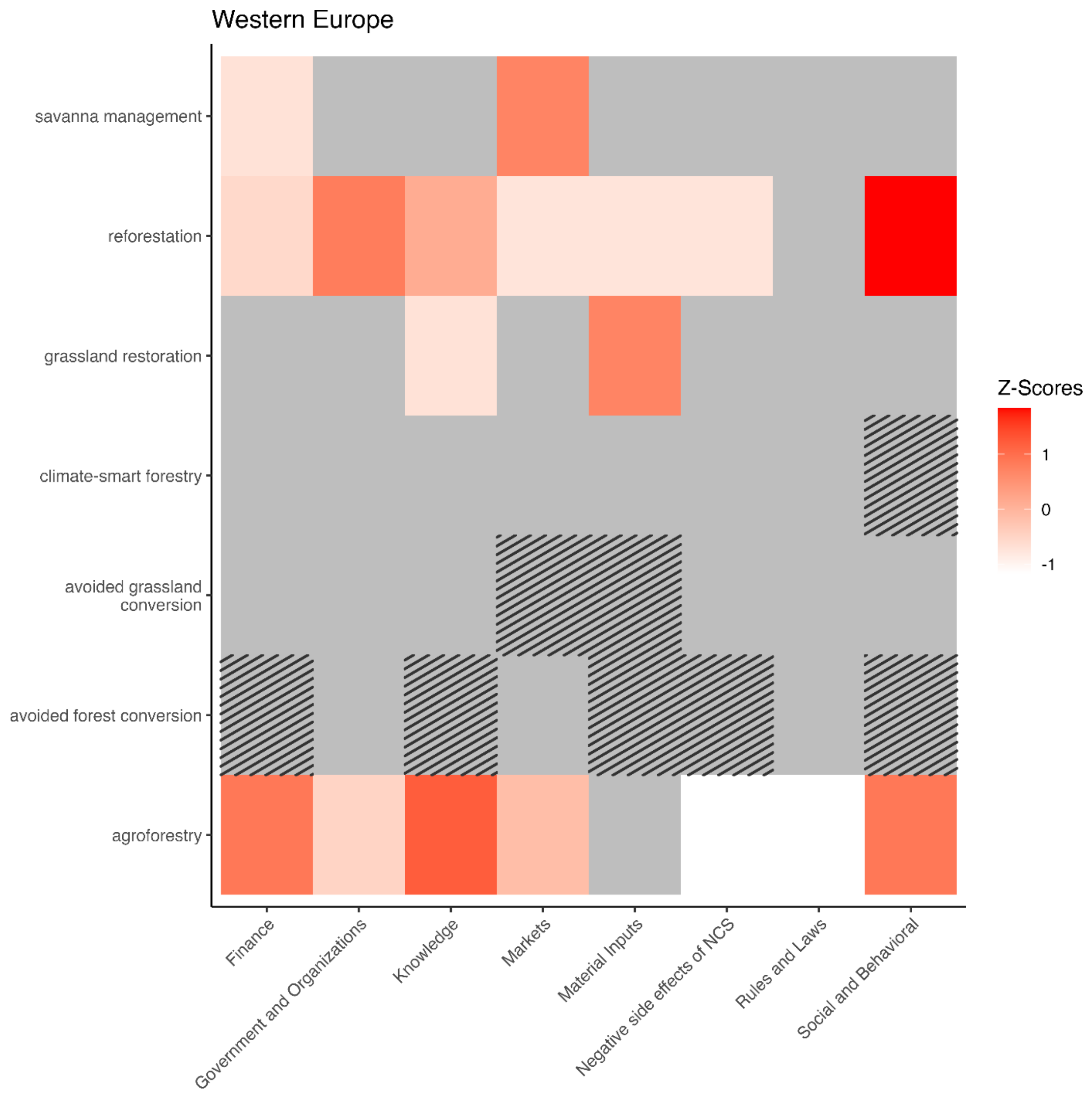

Fig. S17r. Pathway-level subregional z-score heatmaps for all 20 subregions with data. Maps indicate the frequency with which a given constraint category was observed for a given pathway in a subregion compared to the mean frequency for all constraint categories for the pathway in that subregion, expressed as the number of standard deviations from the mean. Maps are color-coded at the pathway level. Grey indicates no data. Hashing indicates undefined z-score (standard deviation = 0).

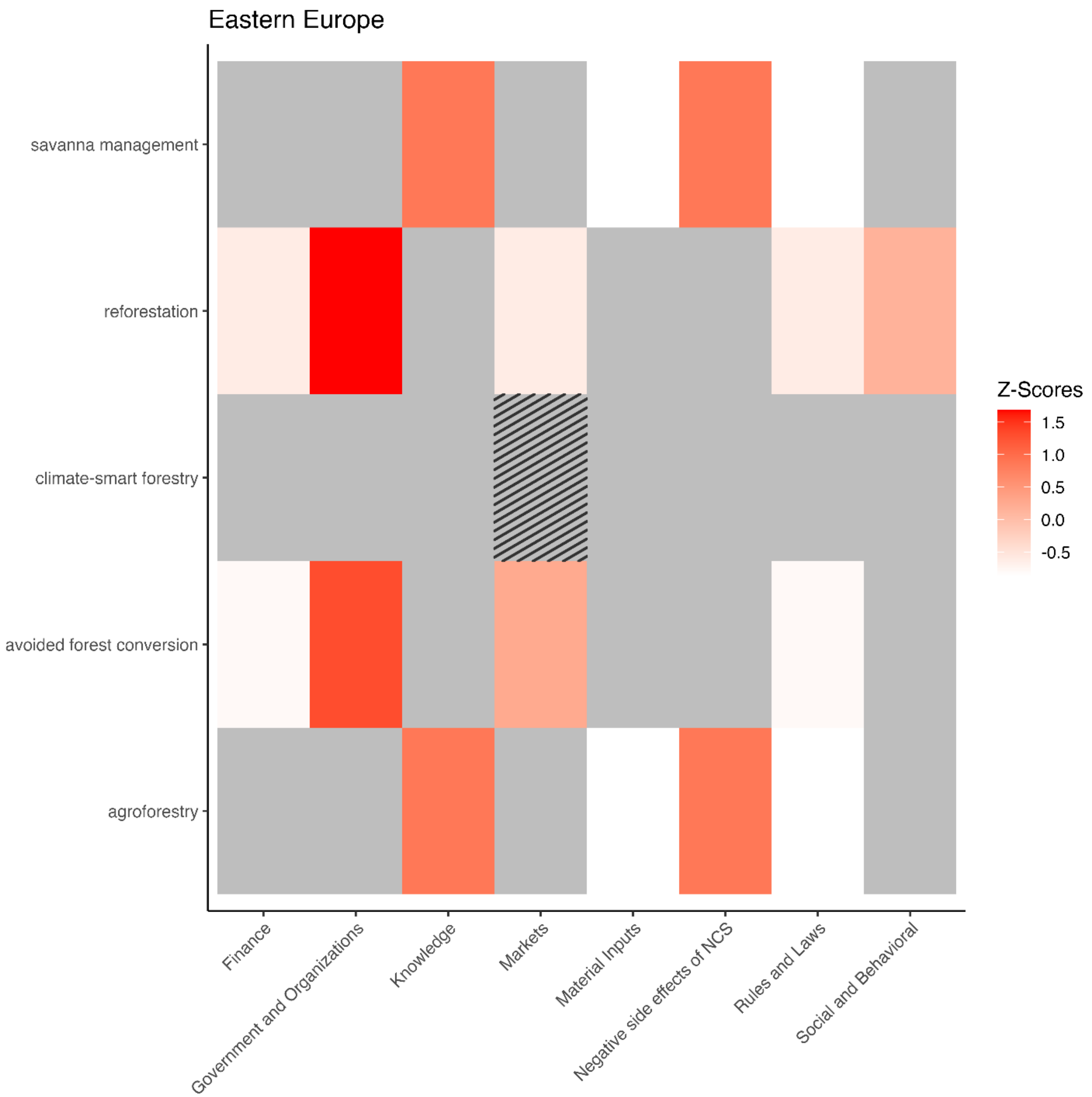

Fig. S17s. Pathway-level subregional z-score heatmaps for all 20 subregions with data. Maps indicate the frequency with which a given constraint category was observed for a given pathway in a subregion compared to the mean frequency for all constraint categories for the pathway in that subregion, expressed as the number of standard deviations from the mean. Maps are color-coded at the pathway level. Grey indicates no data. Hashing indicates undefined z-score (standard deviation = 0).

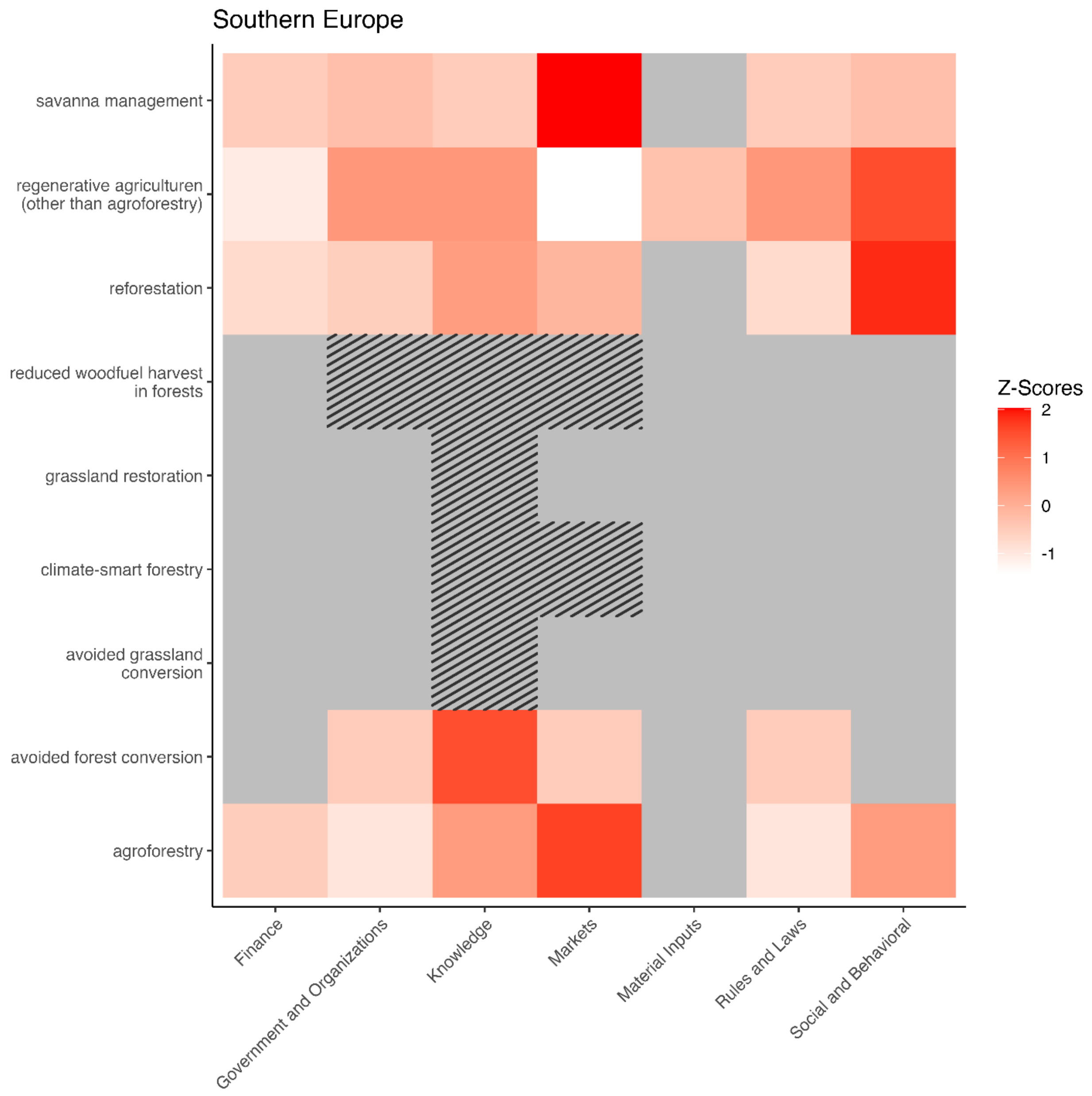

Fig. S17t. Pathway-level subregional z-score heatmaps for all 20 subregions with data. Maps indicate the frequency with which a given constraint category was observed for a given pathway in a subregion compared to the mean frequency for all constraint categories for the pathway in that subregion, expressed as the number of standard deviations from the mean. Maps are color-coded at the pathway level. Grey indicates no data. Hashing indicates undefined z-score (standard deviation = 0).

NCS Pathway	Availability of technical advice for land managers	Aversion to trying new land uses	Burdensome reporting requirements	Concerns over negative equity impacts of NCS	Difficulty identifying, engaging, or coordinating with relevant actors	Financial or other incentives for non-NCS	Greater profitability of alternative land uses	Information about how to design or begin the NCS	Information about how to manage the NCS	Information about market access or prices	Information about on-site benefits of NCS	Information about yields, inputs, or profits	Insecure or uncertain NCS benefit sharing	Insecure or uncertain rights to manage or sell property	Insecure, uncertain, or lack of rights to use natural resources	Labor (external or own) for the NCS	Lack of dispute resolution	Lack of opportunity to participate in or influence the implementation of NCS due to gender, race, ethnicity, or other dimensions of identity
Savanna management	26	5	7	7	18	22	32	47	26	14	15	18	13	35	21	26	20	27
Regen. agric. (excl. agroforestry)	34	30	23	32	31	54	29	40	38	52	31	31	29	40	37	37	13	25
Reforestation	98	67	34	135	84	113	129	122	127	102	89	65	55	112	85	99	61	60
Reduced woodfuel harvest in forests	1	0	0	0	1	0	0	0	0	0	0	0	0	1	0	0	0	0
Peatland restoration	7	3	4	5	4	4	6	**9**	7	6	7	6	1	4	4	2	4	7
Grassland restoration	12	17	11	18	16	24	18	20	24	**25**	17	19	11	17	16	22	7	15
Coastal wetland restoration	12	22	4	3	3	6	**28**	**66**	9	14	14	10	6	3	3	5	4	2
Climate-smart forestry	23	22	14	26	23	29	22	26	**44**	27	19	22	15	21	18	23	9	18
Avoided peatland conversion	14	5	6	4	13	13	5	**17**	**15**	6	6	12	12	6	5	10	3	5
Avoided grassland conversion	4	4	1	3	4	11	**17**	3	1	2	3	1	2	6	6	4	0	2
Avoided forest conversion	52	26	13	29	58	54	**84**	42	44	48	28	29	32	57	35	39	24	40
Avoided coastal wetland conversion	**20**	9	5	5	12	14	19	**36**	17	6	10	13	13	4	5	11	3	3
Agroforestry	110	33	24	33	45	65	67	111	87	63	68	53	36	87	56	72	55	59

NCS Pathway	Lack of policy coordination or implementation capacity	Land manager access to credit for NCS	Land manager access to other funding for NCS	Land manager insurance for NCS assets or outputs	Land manager literacy, numeracy, or technological capabilities	Limited social learning or exchange networks for NCS	Local preferences for non-NCS land uses	Markets for NCS outputs produced by land managers	Markets for carbon sequestered by the NCS	Markets for ecosystem services or biodiversity provided by the NCS	NCS-related corruption	Negative side effects of NCS	Planting stock or other materials for the NCS	Politically influential interests favoring non-NCS	Prices for NCS outputs produced by land managers	Prices for carbon sequestered by the NCS	Prices for ecosystem services or biodiversity provided by the NCS	Project access to credit for NCS	Project access to other funding for NCS	Regulatory barriers to production, transport, or sale of NCS outputs	Skepticism or disinterest in NCS or lack of trust in NCS promoters	Social norms favoring non-NCS land uses	Suitable land for the NCS	Uncertain, or lack of, enforcement of environmental laws	Unclear laws and policies related to NCS outputs/markets	Violent conflict or perceived threat of violence	Water for the NCS	Weak monitoring or enforcement of NCS agreements
Savanna management	**107**	23	**48**	9	18	39	15	14	2	34	2	9	13	20	2	1	11	15	37	21	**48**	13	13	**53**	15	0	15	15
Regen. agric. (excl. agroforestry)	**65**	**56**	**58**	36	39	42	42	41	23	36	9	0	44	32	0	21	43	32	53	42	24	32	27	51	**56**	13	42	50
Reforestation	**282**	110	123	59	95	114	119	90	59	65	22	38	114	94	4	61	75	67	149	104	**205**	61	58	**175**	99	42	83	116
Reduced woodfuel harvest in forests	0	0	1	0	0	0	**2**	0	0	1	0	0	0	0	1	0	0	0	0	1	0	0	0	**2**	0	0	0	0
Peatland restoration	**9**	1	4	1	2	5	5	7	5	2	1	3	3	5	0	4	3	2	4	5	**8**	4	3	**8**	5	2	1	2
Grassland restoration	**34**	14	18	6	19	17	20	23	14	17	0	2	18	15	0	12	19	18	**25**	22	12	16	18	23	**27**	5	17	19
Coastal wetland restoration	**28**	5	11	2	20	10	16	10	14	17	0	6	10	6	0	17	20	5	**29**	6	22	6	11	10	14	1	4	4
Climate-smart forestry	**45**	24	**32**	21	30	24	23	26	21	19	11	2	19	18	2	27	21	21	30	32	16	21	18	27	26	18	18	27
Avoided peatland conversion	12	11	**15**	4	13	**15**	4	6	5	6	3	0	3	14	0	4	5	13	13	6	8	13	3	**15**	6	0	0	11
Avoided grassland conversion	**15**	7	10	6	2	6	4	2	4	9	0	0	7	10	0	11	6	4	6	6	4	4	7	4	**12**	0	6	4
Avoided forest conversion	**127**	48	67	15	49	48	71	38	42	35	14	28	34	54	10	57	43	30	61	41	51	32	23	**114**	42	14	21	78
Avoided coastal wetland conversion	14	11	17	1	19	13	17	5	8	9	2	5	8	13	0	9	11	12	**20**	4	13	14	9	13	8	0	2	12
Agroforestry	**251**	96	**127**	39	67	102	59	78	31	66	12	48	90	35	16	29	43	38	110	62	**127**	37	41	**138**	53	25	54	56

Fig. S18. Frequency counts of individual constraint observations by NCS pathway. Color-coded at the pathway level, with darker shading indicating higher counts. Thick borders and boldface indicate the most frequently observed constraint for each pathway; thin borders and boldface, the second-most frequently observed constraint for each pathway; boldface without borders indicates third-most frequently observed constraint for each pathway.

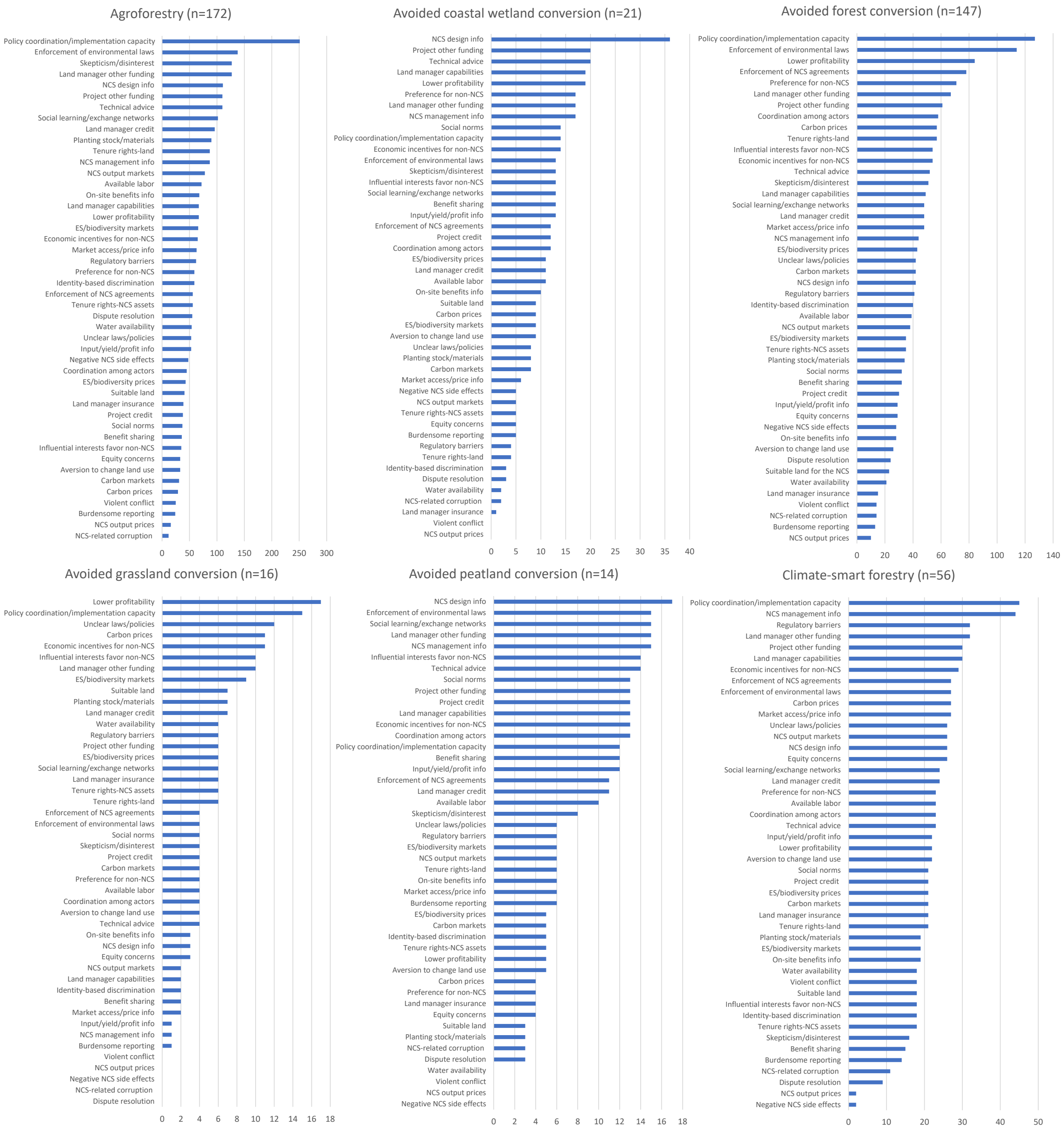

Fig. S19a. Frequency counts of individual constraints by pathway. Combined number (n) of papers and surveys for each pathway shown in parentheses after pathway names. Note different x-axis scales.

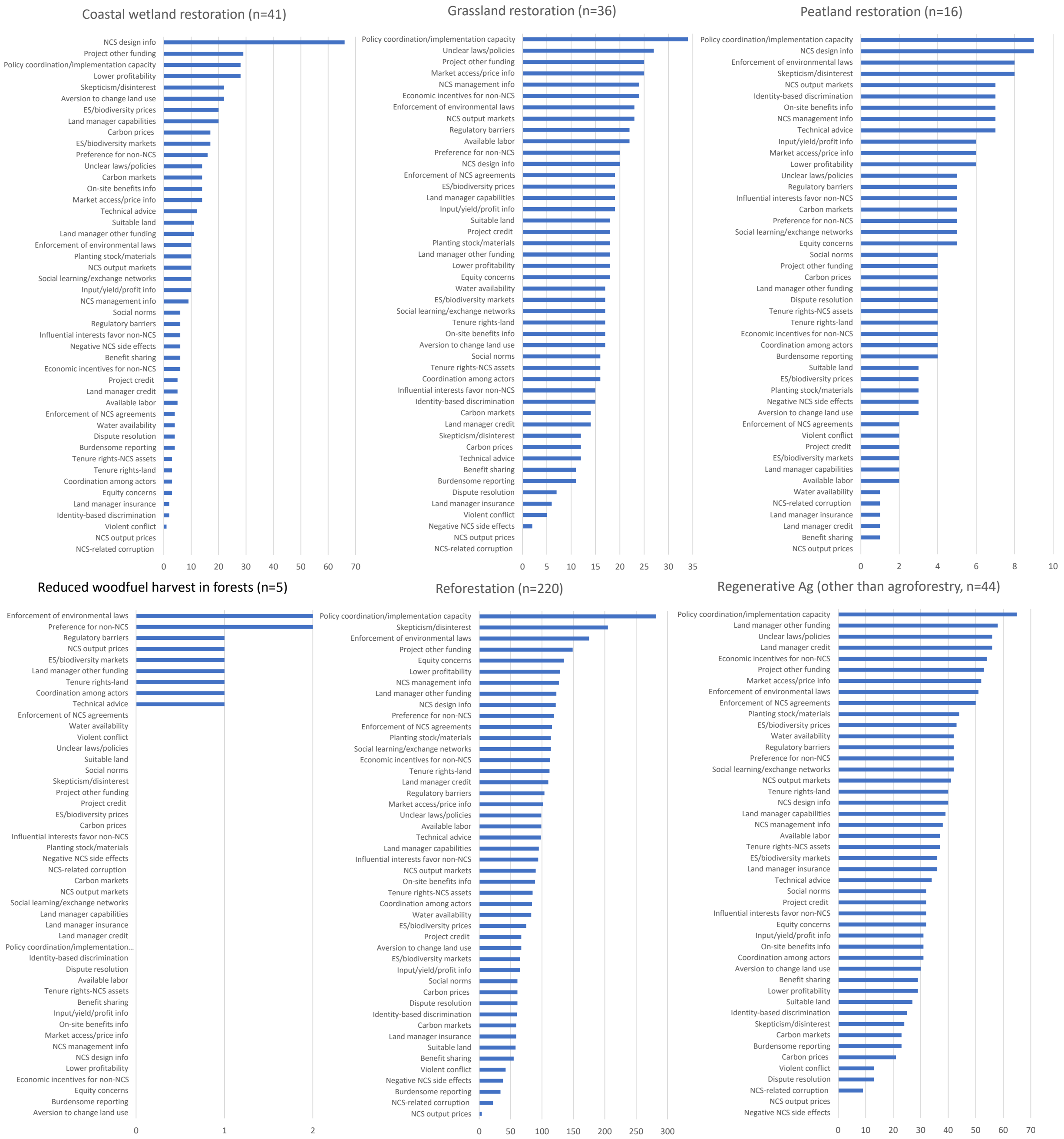

Fig. S19b. Frequency counts of individual constraints by pathway. Combined number (n) of papers and surveys for each pathway shown in parentheses after pathway names. Note different x-axis scales.

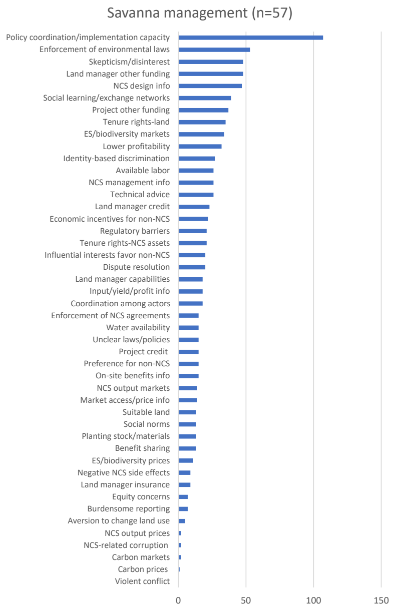

Fig. S19c. Frequency counts of individual constraints by pathway. Combined number (n) of papers and surveys for each pathway shown in parentheses after pathway names. Note different x-axis scales.

	Availability of technical advice for land managers	Aversion to trying new land uses	Burdensome reporting requirements	Concerns over negative equity impacts of NCS	Difficulty identifying, engaging, or coordinating with relevant actors	Financial or other incentives for non-NCS	Greater profitability of alternative land uses	Information about how to design or begin the NCS	Information about how to manage the NCS	Information about market access or prices	Information about on-site benefits of NCS	Information about yields, inputs, or profits	Insecure or uncertain NCS benefit sharing	Insecure or uncertain rights to manage or sell property	Insecure, uncertain, or lack of rights to use natural resources	Labor (external or own) for the NCS	Lack of dispute resolution	Lack of opportunity to participate in or influence the implementation of NCS due to gender, race, ethnicity, or other dimensions of identity	Lack of policy coordination or implementation capacity	Land manager access to credit for NCS	Land manager access to other funding for NCS	Land manager insurance for NCS assets or outputs	Land manager literacy, numeracy, or technological capabilities
Northern America	34	14	3	9	31	9	31	35	20	21	27	11	17	6	7	28	2	10	49	9	35	7	12
Central America	42	26	11	36	28	30	35	80	44	33	27	18	16	35	9	28	55	29	161	25	34	22	42
South America	49	39	21	25	40	67	111	108	71	57	57	51	25	107	49	29	72	14	264	50	53	2	21
Caribbean	12	10	4	8	11	11	6	16	12	11	15	11	6	6	10	7	6	7	11	1	16	7	12
Western Asia	0	0	0	4	0	0	8	8	6	0	6	0	0	0	0	0	0	6	6	0	0	0	0
Central Asia	1	0	0	4	0	0	0	0	0	0	0	0	0	0	0	0	0	0	2	0	0	0	0
Eastern Asia	15	24	6	8	3	10	38	36	22	9	41	7	3	5	4	12	7	2	43	9	17	8	17
Southern Asia	16	3	2	9	12	11	8	7	19	8	5	5	13	9	8	4	0	15	39	4	17	3	13
South-Eastern Asia	33	11	8	15	14	11	60	37	28	11	21	11	11	10	12	31	12	19	67	26	41	6	17
Australia and New Zealand	8	3	3	7	6	7	9	3	0	3	1	4	0	3	3	8	3	0	16	3	10	0	6
Melanesia	1	0	0	0	0	1	1	3	1	1	16	1	0	0	0	1	1	0	1	1	1	0	1
Northern Africa	1	0	4	4	4	0	10	4	1	4	0	4	4	4	0	0	0	4	5	5	5	0	0
Western Africa	58	49	45	63	50	84	70	80	69	75	34	58	54	90	93	65	9	41	116	121	114	93	73
Eastern Africa	90	56	33	79	85	137	32	95	115	127	44	74	56	109	89	99	30	112	159	129	140	49	138
Central Africa	23	1	5	11	22	23	5	19	19	1	6	19	19	6	5	24	5	3	22	23	24	1	19
Southern Africa	0	1	1	2	1	0	0	1	0	0	2	0	0	0	0	1	1	1	2	0	2	0	1
Northern Europe	1	0	0	3	0	4	16	2	5	0	1	1	0	1	1	6	0	0	7	0	11	0	0
Western Europe	10	5	0	7	3	0	5	0	2	0	3	1	0	0	0	2	0	0	8	0	6	0	0
Eastern Europe	4	0	0	1	0	4	2	0	0	4	0	0	0	0	0	4	0	0	5	0	2	0	0
Southern Europe	15	1	0	5	2	0	9	5	5	0	1	3	1	2	1	1	0	0	6	0	3	1	1

	Limited social learning or exchange networks for NCS	Local preferences for non-NCS land uses	Markets for NCS outputs produced by land managers	Markets for carbon sequestered by the NCS	Markets for ecosystem services or biodiversity provided by the NCS	NCS-related corruption	Negative side effects of NCS	Planting stock or other materials for the NCS	Politically influential interests favoring non-NCS	Prices for NCS outputs produced by land managers	Prices for carbon sequestered by the NCS	Prices for ecosystem services or biodiversity provided by the NCS	Project access to credit for NCS	Project access to other funding for NCS	Regulatory barriers to production, transport, or sale of NCS outputs	Skepticism or disinterest in NCS or lack of trust in NCS promoters	Social norms favoring non-NCS land uses	Suitable land for the NCS	Uncertain, or lack of, enforcement of environmental laws	Unclear laws and policies related to NCS outputs/markets	Violent conflict or perceived threat of violence	Water for the NCS	Weak monitoring or enforcement of NCS agreements
Northern America	21	14	17	8	27	0	7	17	10	0	20	20	7	22	7	35	20	10	10	24	2	2	3
Central America	54	40	28	18	38	2	0	24	22	3	21	24	11	47	33	73	16	26	93	34	1	21	32
South America	87	71	65	72	39	10	4	60	67	8	46	45	21	107	51	154	36	46	219	56	15	27	88
Caribbean	8	6	7	7	6	0	0	6	4	0	6	6	6	11	12	10	6	11	12	12	1	10	7
Western Asia	0	0	6	0	10	0	0	0	0	0	0	0	0	0	0	0	0	0	0	0	0	0	0
Central Asia	0	0	0	0	0	0	2	0	0	0	0	0	0	0	0	2	0	1	0	0	0	0	0
Eastern Asia	11	15	4	12	11	1	1	14	24	0	10	21	4	31	3	85	6	13	7	11	0	0	7
Southern Asia	14	22	14	6	6	13	24	13	1	3	22	10	6	8	22	13	1	4	5	3	0	4	9
South-Eastern Asia	20	19	20	10	10	10	14	25	18	16	30	14	11	28	19	36	14	4	23	18	4	0	13
Australia and New Zealand	0	11	0	3	11	0	26	0	6	0	12	10	7	19	5	3	8	11	0	7	0	0	3
Melanesia	0	31	1	1	1	0	15	1	0	0	1	1	0	1	0	0	0	0	1	1	1	0	1
Northern Africa	4	0	10	4	14	0	0	4	0	1	4	4	4	0	4	0	0	0	4	4	4	0	4
Western Africa	80	64	48	18	52	7	8	77	66	3	12	66	55	98	107	42	58	58	97	96	25	94	86
Eastern Africa	111	90	113	60	60	31	20	111	71	0	60	74	106	130	74	44	67	41	129	90	58	99	114
Central Africa	23	1	5	4	5	2	1	5	19	1	7	0	19	23	5	3	19	4	26	5	9	5	23
Southern Africa	0	1	1	0	1	0	0	0	1	0	0	1	0	1	1	2	1	0	1	1	0	0	1
Northern Europe	0	6	0	5	0	0	8	3	0	0	1	0	0	2	0	7	0	0	0	0	0	0	1
Western Europe	1	1	0	0	4	0	3	2	5	0	0	0	0	5	1	11	0	1	0	0	0	0	0
Eastern Europe	0	1	0	0	0	0	8	0	2	0	0	2	0	0	2	0	0	0	0	0	0	0	0
Southern Europe	1	4	1	0	21	0	0	1	0	0	1	2	0	4	6	18	1	1	6	1	0	1	2

Fig. S20. Frequency counts of individual constraint observations by UN sub-region. Color-coded at the subregion level, with darker shading indicating higher counts. Thick borders and boldface indicate the most frequently observed constraint for each subregion; thin borders and

boldface, the second-most frequently observed constraint for each subregion; boldface without borders indicates third-most frequently observed constraint for each subregion.

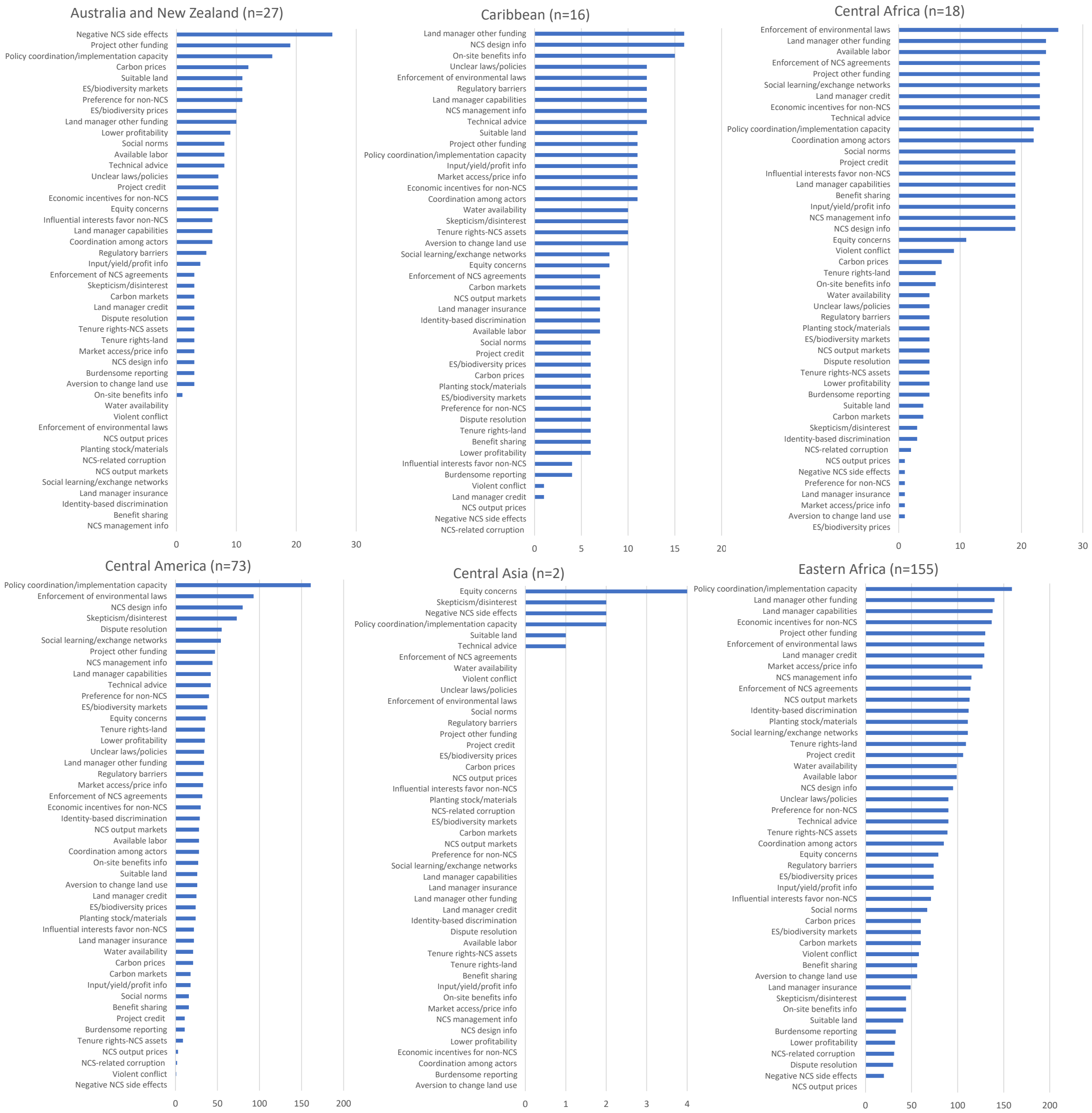

Fig. S21a. Frequency counts of individual constraints by UN subregion. Note different x-axis scales.

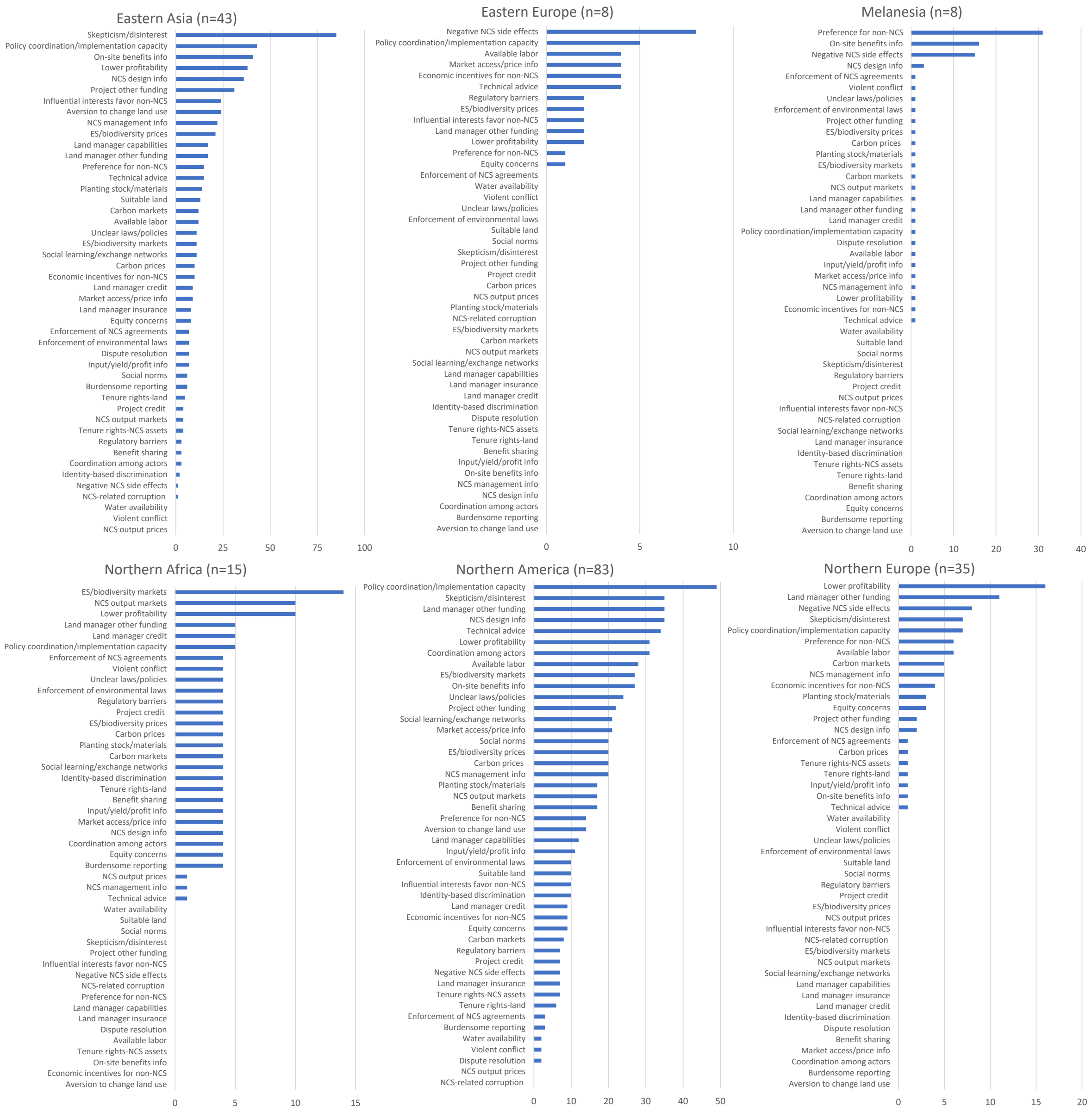

Fig. S21b. Frequency counts of individual constraints by UN subregion. Note different x-axis scales.

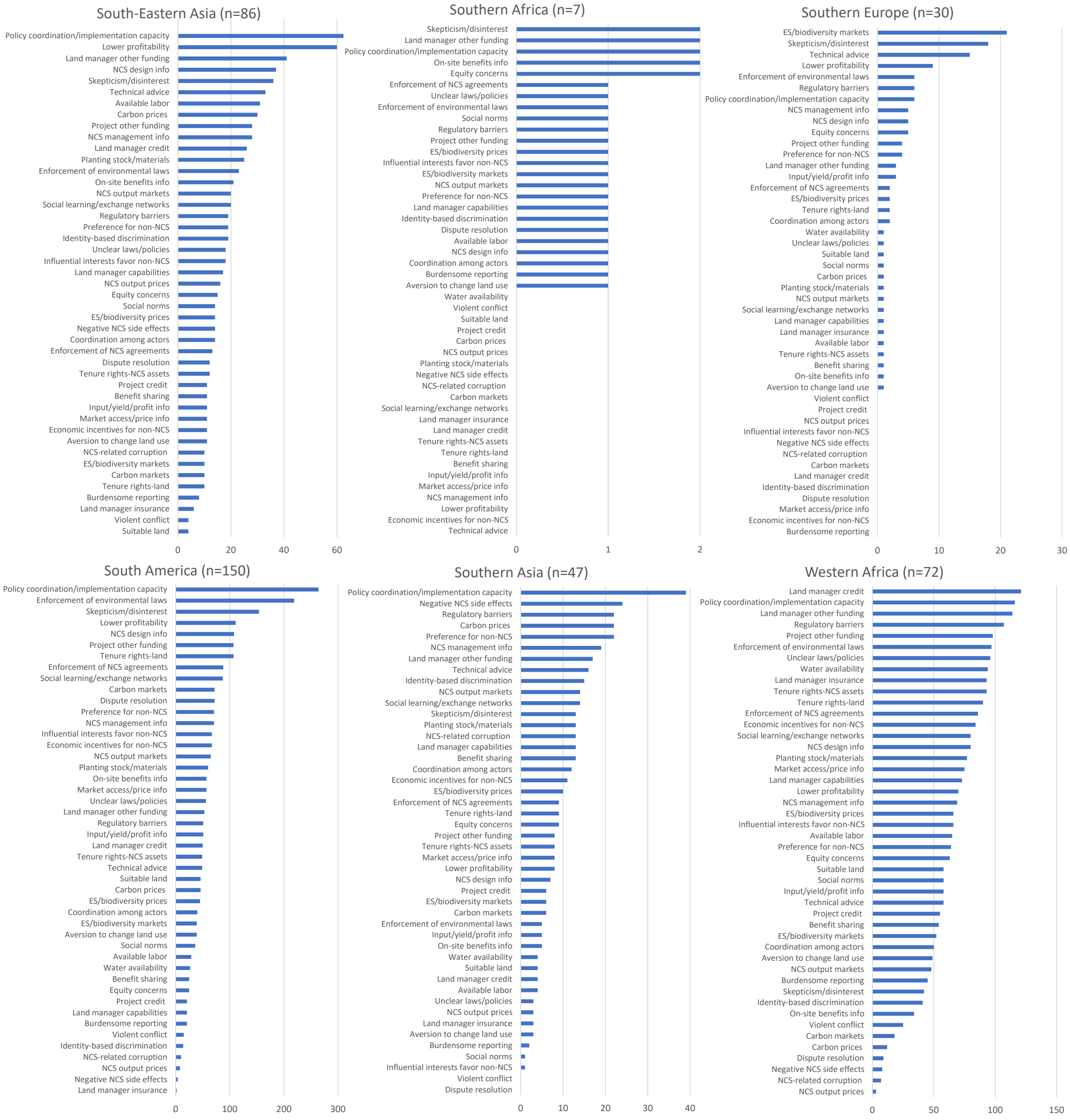

Fig. S21c. Frequency counts of individual constraints by UN subregion. Note different x-axis scales.

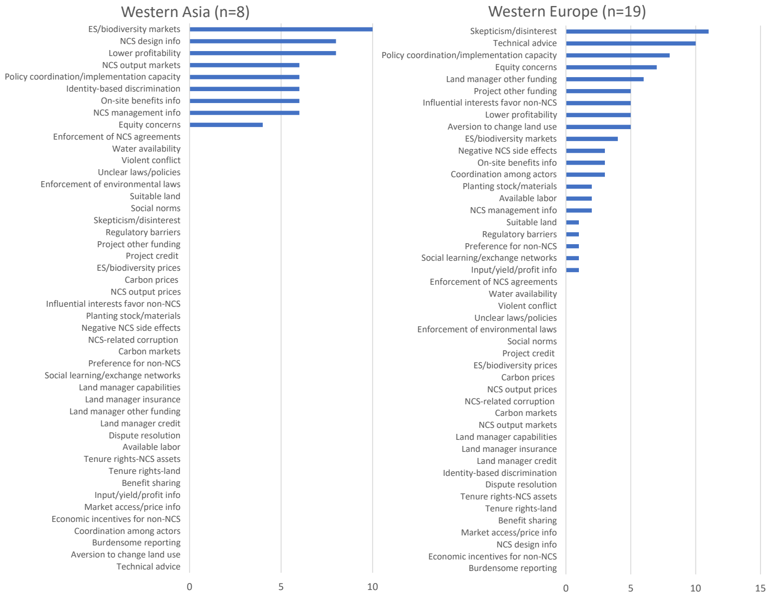

Fig. S21d. Frequency counts of individual constraints by UN subregion. Note different x-axis scales.

Category	Constraint name	Abbreviated name	Description
Material Inputs	Planting stock or other materials for the NCS	Planting stock/ materials	Limited or uncertain availability or quality, or high or uncertain cost of planting and related inputs
	Labor (external or own) for the NCS	Available labor	Limited or uncertain availability or quality, or high or uncertain cost of labor (for land managers or NCS project). Includes lack of sufficiently skilled labor.
	Suitable land for the NCS	Suitable land	Limited or uncertain availability or quality, or high or uncertain cost of land. Includes instances where the NCS in question cannot be implemented due to biophysical constraints (e.g., due to size of farm machinery; steep slopes; lack of accessibility; water-logging; lack of soil depth or quality)
	Water for the NCS	Water availability	Limited or uncertain availability or quality, or high or uncertain cost of water for establishment or maintenance of the NCS; includes insufficient precipitation, lack of water for irrigation, and lack of irrigation infrastructure
Finance	Land manager access to credit for NCS	Land manager credit	Limited or uncertain availability, or too high or uncertain cost of credit for land managers for the NCS
	Land manager access to other funding for NCS	Land manager other funding	Limited or uncertain availability, or too high or uncertain cost of other (non-credit) funding for land managers for the NCS
	Land manager insurance for NCS assets or outputs	Land manager insurance	Limited or uncertain availability, or too high or uncertain cost of NCS insurance for land managers
	Project access to credit for NCS	Project credit	Limited or uncertain availability, or too high or uncertain cost of credit for entities that implement or support NCS; includes institutional entities such as communities and private entities that implement or facilitate NCS
	Project access to other funding for NCS	Project other funding	Limited or uncertain availability, or too high or uncertain cost of (non-credit) funding entities that implement or support NCS; includes institutional entities such as communities or government agencies, and private entities, that implement or facilitate NCS
	Burdensome reporting requirements	Burdensome reporting	Limited or uncertain capacity to manage demanding donor/creditor relationships or reporting requirements, or high or uncertain cost of such reporting
Markets	Markets for NCS outputs produced by land managers	NCS output markets	Limited or uncertain availability of output (e.g., food or wood) markets, or high or uncertain costs associated with accessing output markets (due to lack of transport infrastructure or services, or high transaction costs in general)
	Markets for carbon sequestered by the NCS	Carbon markets	Limited or uncertain availability of carbon markets, or high or uncertain costs associated with accessing carbon markets (due to lack of needed intermediate service providers or high transaction costs in general)
	Markets for ecosystem services or biodiversity provided by the NCS	ES/biodiversity markets	Limited or uncertain availability of relevant ecosystem services (e.g., water quality or flow regulation) or biodiversity markets, or high or uncertain costs associated with accessing such markets
	Prices for NCS outputs produced by land managers	NCS output prices	Low or uncertain prices of NCS outputs (e.g., food or wood)
	Prices for carbon sequestered by the NCS	Carbon prices	Low or uncertain prices of carbon
	Prices for ecosystem services or biodiversity provided by the NCS	ES/biodiversity prices	Low or uncertain prices of ecosystem services (e.g., water quality or flow regulation) or biodiversity
	Greater profitability of alternative land uses	Lower profitability	(Uncertainty about whether) Alternative land use generates higher net profits than NCS, or alternative land use has preferred cash flow structure (e.g., where delay in NCS income is considered to be too long)

Negative side effects of NCS	Negative side effects (resource use, human health, property damage, wildlife conflict)	Negative NCS side effects	Reductions in yields of existing crops, both subsistence or commercial (regardless of whether overall profitability of NCS is higher); negative impacts on human health; property damage; increased complexity or difficulty of land management; negative biodiversity impacts; conflict with neighbors; conflict with wildlife attracted by the NCS
Knowledge	Land manager literacy, numeracy, or technological capabilities	Land manager capabilities	Land manager reading, calculation, or technological abilities constrain NCS adoption (e.g., inability to read NCS educational or instructional materials; lack of ability to apply on-farm yield or profit calculators); or high or uncertain cost associated with improving land managers' literacy, numeracy or technological capabilities
	Information about how to design or begin the NCS	NCS design info	Limited or uncertain availability of NCS design or installation information for land managers, NCS project staff or government employees; or limited or uncertain quality of this information, or high or uncertain cost associated with accessing this information
	Information about how to manage the NCS	NCS management info	Limited or uncertain availability of NCS operations or management information for land managers, NCS project staff or government employees; or limited or uncertain quality of this information, or high or uncertain cost associated with accessing this information
	Availability of technical advice for land managers	Technical advice	Limited or uncertain availability or quality, or high or uncertain cost of NCS technical advice or extension services for land managers
	Information about yields, inputs, or profits	Input/yield/ profit info	Limited or uncertain availability or quality of information about NCS yields, inputs, or profits. Includes both absence of evidence, and lack of access to evidence.
	Information about market access or prices	Market access/ price info	Limited or uncertain availability of information about NCS market access or prices (for NCS outputs, carbon, ecosystem services or biodiversity)
	Information about on-site benefits of NCS	On-site benefits info	Limited availability or high uncertainty, or limited or uncertain quality of information about NCS performance (e.g., restoration success and associated climate mitigation; yield) and "co-benefits" (e.g., improved soil fertility or moisture, temperature mitigation for people or livestock, crop yield, or income diversification)
Social and Behavioral	Local preferences for non-NCS land uses	Preference for non-NCS	Preferences of land managers or local residents for non-NCS land uses. Preferences of govt officials or other influential parties are captured in separate constraint "Politically influential interests favoring non-NCS."
	Aversion to trying new land uses	Aversion to change land use	Generic dislike of any change in current land uses
	Skepticism or disinterest in NCS or lack of trust in NCS promoters	Skepticism/ disinterest	Land managers or local community or leaders are skeptical of or disinterested in NCS, or mistrust NCS promoters. Most cases involved disinterest rather than mistrust. Includes cases where NCS are hindered by concerns over lack of transparency of NCS implementation or planning
	Social norms favoring non-NCS land uses	Social norms	Cultural, religious, or other norms lead land managers to prefer non-NCS land uses (e.g., view of non-crop uses as 'unproductive' or otherwise less acceptable). Includes community pressure preventing land managers who are otherwise interested in NCS from adopting NCS.
	Concerns over negative equity impacts of NCS	Equity concerns	Land managers', or local community's or leaders' perception of actual or potential undesirable equity outcomes of NCS that inhibit NCS adoption or continuation
	Lack of opportunity to participate in or influence the implementation of NCS due to gender, race, ethnicity, or other dimensions of identity	Identity-based discrimination	Identity-based discrimination that prevents certain racial, ethnic, religious, gender, or other groups from adopting NCS or from shaping the implementation of NCS
	Limited social learning or exchange networks for NCS	Social learning/ exchange networks	Lack of, or lack of access to, formal or informal networks or programs that facilitate mutual learning or sharing of experiences about NCS among implementers, communities or government officials

	Difficulty identifying, engaging, or coordinating with relevant actors	Coordination among actors	Challenges in identifying, engaging, or coordinating with actors whose support or lack of opposition is required to make NCS implementation feasible in a given case, due to the large number, wide geographic dispersion, or conflicting attitudes of actors.
	Lack of dispute resolution	Dispute resolution	Absence of formal or informal mechanisms for resolving conflicts among individuals or groups that negatively impacts the establishment or maintenance of the NCS
Rules and Laws	Insecure or uncertain rights to manage or sell property	Tenure rights-land	Lack of de jure, de facto, or perceived tenure rights – specifically, the right to manage or sell the land
	Insecure, uncertain, or lack of rights to use natural resources	Tenure rights-NCS assets	Lack of de jure, de facto, or perceived tenure rights – specifically, the right to use, harvest or sell specific components of the land (e.g., trees)
	Regulatory barriers to production, transport, or sale of NCS outputs	Regulatory barriers	Permits, licenses, or other administrative conditions that must be met for legal adoption of NCS or for production, harvest, transport or sale of NCS outputs (e.g., transport permits for wood)
	Insecure or uncertain NCS benefit sharing	Benefit sharing	Lack of, or doubts about, desirable distribution of benefits from NCS among relevant parties (e.g., land managers, other community member, funders, technical service providers, local authorities, or government representatives or agencies)
	NCS-related corruption	NCS-related corruption	Illegal rent-seeking by government officials or private actors that affects NCS management or effectiveness but that may not be particular to the NCS (e.g., corruption among government forest or transportation department officials)
	Unclear laws and policies related to NCS outputs/markets	Unclear laws/ policies	Uncertainty about laws, policies or regulations that govern or may pertain to the adoption of NCS or the sale of NCS outputs (e.g., carbon, wood, food crops)
	Financial or other incentives for non-NCS	Economic incentives for non-NCS	Subsidies, reduced taxes, preferential access to credit or insurance, or other government incentives for non-NCS land uses that are not provided for NCS
Government and organizations	Lack of policy coordination or implementation capacity	Policy coordination, implementation capacity	Uncoordinated, conflicting, or duplicative (and thus compliance burden-raising) policies (among economic sectors, different administrative units, different organizations, or combinations thereof) that negatively affect NCS adoption and deployment; or lack of capacity (technology, human resources) to implement NCS-relevant policies
	Uncertain, or lack of, enforcement of environmental laws	Enforcement of environmental laws	Complete or partial lack, or unpredictability, of enforcement of environmental laws that affect the NCS
	Weak monitoring or enforcement of NCS agreements	Enforcement of NCS agreements	Absent or insufficient compliance monitoring of formal or informal agreements underlying NCS implementation (e.g., in payments for environmental services [PES] programs for restoration, conservation, or changes in land management)
	Violent conflict or perceived threat of violence	Violent conflict	Presence or fear of civil strife or military conflict, or perception of lack of government's ability to guarantee individuals' physical safety
	Politically influential interests favoring non-NCS	Influential interests favor non-NCS	Individuals or small groups (private or government) with the ability to meaningfully affect policies favor non-NCS land uses. Includes political prioritization of competing goals and lack of urgency for NCS implementation.

Table S1. Descriptions of NCS constraints.

This paper	IPCC [2]	Schulte et al. [3]	Roe et al. [1]	Karki et al. [4]
NCS implementation constraint categories and constraints	*AFOLU barriers and opportunities*	*Categories of NCS enabling factors*	*NCS feasibility dimensions and indicators*	*Barriers and sub-barriers to LMT deployment*
Markets	Socio-economic	Economic	Economic	Economic
• Markets for NCS outputs produced by land managers (1)<br>• Markets for carbon sequestered by the NCS (2)<br>• Markets for ecosystem services or biodiversity provided by the NCS (3)<br>• Prices for NCS outputs produced by land managers (4)<br>• Prices for carbon sequestered by the NCS (5)<br>• Prices for ecosystem services or biodiversity provided by the NCS (6)<br>• Greater profitability of alternative land uses (7)	• Design and coverage of financing mechanisms (2,3,9)<br>• Scale and accessibility of financing (4,5,6,8,9,11,12)<br>• Risk aversion and uncertainty coupled with significant upfront investments and time lags (18,*22*,*7*)<br>• Impacts on poverty and food security (36)<br>• Cultural values and social acceptance (*21*,*23*,24)	• Delivery of benefits<br>• Accessibility (*1*)<br>• Labor availability (projects, programs, or gov. ministries) (37,43)<br>• Market competitiveness (7)	• GDP per capita (PPP)<br>• Forest rents ($/ha)<br>• Agricultural value added ($/ha)<br>• Ease of doing business<br>• Ease of obtaining a bank loan (8,11)	• *Costs* (unable to afford specialized machinery; large initial investment, expensive to deploy at a scale where there is large potential) (9,11)<br>• *Income* (potential for income decline due to trade-offs; transitional period with higher production costs and lower income; lack of incentives) (2,7)<br>• *Value* (difficulty of monetizing non-market benefits/ES) (2,3,4)
Finance		Financial		
• Land manager access to credit for NCS (8)<br>• Land manager access to other funding for NCS (9)<br>• Land manager insurance for NCS assets or outputs (10)<br>• Project access to credit for NCS (11)<br>• Project access to other funding for NCS (12)<br>• Burdensome reporting requirements (13)		• Performance-based finance (2,3)<br>• Secure funding (9,12)<br>• Donor finance (9,12)<br>• Financial services (10)		
Knowledge	Technological	Technical	Technological	Technological
• Land manager literacy, numeracy, or technological capabilities (14)<br>• Information about how to design or begin the NCS (15)<br>• Information about how to manage the NCS (16)<br>• Availability of technical advice for land managers (17)<br>• Information about yields, inputs, or profits (18)<br>• Information about market access or prices (19)<br>• Information about on-site benefits of NCS (20)	• Monitoring, reporting, and verification needs (13,17)	• Technical assistance (17)<br>• Monitoring, reporting, verification (13)<br>• Land-use planning (15)<br>• Compatible practices (15,16)<br>• Research (15,16,18,20)	• Access to information and communications (15,16,18,19,20)<br>• Market access and infrastructure (1,2)<br>• Agricultural total factor productivity	• *Complexity* (difficult to adopt due to the complexity of LMTs for farmers/land managers; requiring high management skills) (14,15,16)<br>• *Resources* (difficulty in access to specialised machinery; unavailability of transportation infrastructure; Lack of access to inputs; Limited/no access to credit; Limited extension facilities) (8,11,17,42)<br>• *Development* (lack of development of efficient monitoring, reporting and verification; large uncertainties about the benefits of an LMT; limited scientific understanding of land suitability; technological readiness) (14,15,18,20)
Social - Behavioral		Social	Socio-cultural	Socio-cultural

• Local preferences for non-NCS land uses (21)<br>• Aversion to trying new land uses (22)<br>• Skepticism or disinterest in NCS or lack of trust in NCS promoters (23)<br>• Social norms favoring non-NCS land uses (24)<br>• Concerns over negative equity impacts of NCS (25)<br>• Lack of opportunity to participate in or influence the implementation of NCS due to gender, race, ethnicity, or other dimensions of identity (26)<br>• Limited social learning or exchange networks for NCS (27)<br>• Difficulty identifying, engaging, or coordinating with relevant actors (28)<br>• Lack of dispute resolution (29)		• IPLC engagement (*26*)<br>• Acceptance (local) (21,23,24)<br>• Stakeholder consultation (*28*)<br>• Recognition of traditional values<br>• Free, prior, informed consent (FPIC)	• Personal rights (political rights, freedom of expression, freedom of religion, access to justice, property rights for women)<br>• Nutrition and basic medical care	• *Norms and values* (traditional beliefs, cultural affiliation to a traditional production system, cultural norm) (21,24)<br>• *Knowledge and perception* (lack of knowledge, limited awareness of value and benefits, low social acceptance, social pressure against LMTs, perceived threats from LMTs) (18,20,23,46)<br>• *Behavior* (difficulty in long-term decision for transition, lack of trust, habit of relying on conventional practice) (23,22)<br>**Ethical**<br>• *Conflicts* (risks of land grabbing; Issue of equitable benefit sharing; Issue of social conflicts) (25,29,30,33)<br>• *Trade-offs* (land availability and competition with other land uses; possible increase in food prices and compromise food security; Negative effect on the environment) (45,46)<br>• *Fairness* (limited access of women and minority groups to resources and land; no consideration of the rights of Indigenous People and local communities) (26)
**Rules and laws**	**Institutional**	**Institutional**	**Institutional**	**Institutional**
• Insecure or uncertain rights to manage or sell property (30)<br>• Insecure, uncertain, or lack of rights to use natural resources (31)<br>• Regulatory barriers to production, transport, or sale of NCS outputs (32)<br>• Insecure or uncertain NCS benefit sharing (33)<br>• NCS-related corruption (34)<br>• Unclear laws and policies related to NCS outputs or markets (35)<br>• Financial or other incentives for non-NCS (36)	• Transparent and accountable governance (34,38,39)<br>• Clear land tenure and land-use rights (30,31)<br>• Institutional capacity (37)	• Institutional frameworks (35)<br>• Land/customary tights (30,31)<br>• Realistic requirements (*13*)<br>• Enforcement (38,39)<br>• Regulatory support (32,35)<br>• Clear carbon rights (33)	• Voice and accountability (26)<br>• Political stability and absence of violence (40)<br>• Government effectiveness (37,38,39)<br>• Regulatory quality (*35*)<br>• Rule of law (34,38)<br>• Control of corruption (34)<br>• Tenure insecurity (30,31)	• *Policy* (lack of policy support mechanism to set explicit incentives; lack of policy implementation; disinterest of policymakers) (37,41)<br>• *Governance* (lack of cross-sectoral responsibility-sharing; top-down approach; coordination between stakeholders; lack of proper monitoring) (38,37)<br>• *Regulation* (counter-productive public policies and legislation; lack of standards and protocols to measure carbon sequestration) (32,35,36)
**Government and organizations**		**Political**		
• Lack of policy coordination or implementation capacity (37)<br>• Uncertain, or lack of, enforcement of environmental laws (38)<br>• Weak monitoring or enforcement of NCS agreements (39)<br>• Violent conflict or perceived threat of violence (40)		• Collaboration/coordination (37,28)<br>• Acceptance (leadership) (*41*)<br>• Political integrity (34)<br>• Transparency<br>• Accountability		

• Politically influential interests favoring non-NCS (41)			
Material Inputs	Ecological	Biophysical	Geophysical
• Planting stock or other materials for the NCS (42)<br>• Labor (external or own) for the NCS (43)<br>• Water for the NCS (44)<br>• Suitable land for the NCS (45)	• Land and water availability (44,45)<br>• Specific soil conditions, water availability, GHG emission potential, natural variability and resilience (18,20)<br>• Adaptation benefits and biodiversity conservation	• Land conditions (45)<br>• Climatic conditions<br>• Species diversity<br>• Carbon stock potential	• Total land-based technical mitigation potential / total land area (tCO_2/ha)
			Environmental-ecological
			• Environmental performance index (EPI)
Negative side effects (46)			

Table S2. Comparison of constraint categories and constraints used in this paper with those used in other recent studies. Italicized numbers indicate potential or partial match with the respective constraint used in this paper. AFOLU – Agriculture, forestry and other land uses. ES – ecosystem services. GHG – greenhouse gas. LMT – land-based mitigation technology. NCS – natural climate solution.

Region	Number of times countries in region are represented in papers	Number of survey projects in region	Number of countries in region covered in:		Number of times countries in region are studied in papers or survey	Number of countries in region covered in literature or survey	Share of all countries in region covered in papers or survey
			Papers	Survey			
Africa	116	63	41	22	179	44	81%
Americas	184	59	25	15	243	26	79%
Asia	154	25	37	5	179	37	77%
Europe	79	2	26	2	81	26	59%
Australia-New Zealand-Melanesia	16	5	4	2	21	4	57%

Table S3. Country coverage in literature review and survey.

Survey Questionnaire

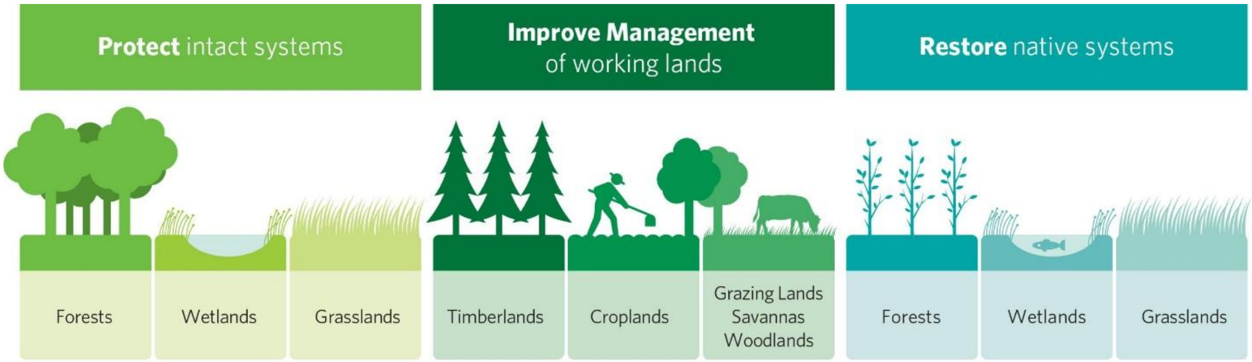

Note: Graphic cropped and text modified from graphical abstract in Griscom et al. (2019), We need both natural and energy solutions to stabilize our climate, Global Change Biol. 25: 1889–1890, https://doi.org/10.1111/gcb.14612, published under a CC BY 4.0 Attribution 4.0 International license (https://creativecommons.org/licenses/by/4.0/).

Global survey of constraints, solutions, and enabling factors for scaling of projects that implement Natural Climate Solutions

Note: Hold your cursor over text that looks like **this** *for extra information, like definitions or explanations. [Note: the pop-up explanations in the original online survey have been converted to footnotes in this Supplementary Information.]*

Welcome to the first global survey of projects that implement Natural Climate Solutions. Natural Climate Solutions (NCS) are conservation, restoration, or improved management activities in terrestrial or aquatic systems that reduce greenhouse gas emissions or sequester carbon dioxide. They include common practices such as integration of trees in pasture or croplands; soil conservation or restoration measures (for example, cover crops, reduced tillage, or improved fallow); improved crop fertilizer or water management; improved fire management; improved livestock density, rotation, and manure management; and restoration or avoided conversion of forests, grasslands or wetlands.

This survey seeks to learn from any project that implements NCS activities, whether exclusively or as part of a broader portfolio of activities, and regardless of whether the project seeks to achieve climate change mitigation or adaptation, livelihood or human well-being improvements, biodiversity conservation, or some other objective.

Why we need this survey

The biophysical potential for NCS to make large contributions to climate change mitigation is well-documented. Yet, **projects**[1] that implement NCS often face a variety of constraints, and

[1] An NCS "project" here is defined as 1) the direct implementation of one or several NCS by an entity or group of entities on a discrete site or sites (for example, a peatland rewetting and agroforestry project carried out by a local government with the assistance of a research entity); or 2) the provision, by others, of services that support the direct implementation of specific NCS practices across diverse sites by a discrete set of land owners or managers (for example, the provision of technical extension services that support the adoption of smallholder agroforestry in a particular geography).

these constraints remain poorly understood. Without understanding the constraints NCS projects face, it is uncertain where and how much NCS potential is limited by implementation challenges; project planning is more uncertain; and it is more difficult to consider where and how NCS may be scaled.

To address this information gap, we (the signatories below) have launched a multi-institutional, global-scale effort to engage projects that deploy NCS, with the goal of collecting information on the constraints they face when implementing NCS, identifying possible solutions to those constraints, and better understanding key factors that inhibit the scaling of NCS projects. **This survey is a central part of that effort.**

We are contacting you because of your deep knowledge of projects that implement NCS activities.

Your participation in this survey is critical to help better understand what is needed to enable faster progress on mitigating climate change and achieving the many other desirable outcomes that NCS deliver.

This survey has three parts and should take approximately 45 minutes to complete.

What this survey will do for your project(s) and for efforts to limit climate change and further other important outcomes that NCS can deliver

We believe that participation in this survey will allow you to critically reflect on project implementation, through a systematic and comprehensive self-assessment of the constraints your project may face, and of the potential solutions for addressing those constraints.

In addition, the insights gained from this survey will be used to:

- Help projects globally communicate the key constraints they are facing, via a mapping portal;
- Inform private and public decision-makers about these constraints and about key actions needed to mitigate these constraints in a geographically explicit manner;
- Inform private and public decision-makers about key enabling conditions for scaling of NCS that currently are not met in specific geographies;
- Inform other (current or future) NCS projects about potential constraints and key actions to mitigate these constraints;
- Identify important solutions for projects with similar constraints

In the first quarter of 2023, we will share the initial results of this survey, including country and regional analyses, with you and all other survey participants. The results also will be published in an open-access journal article and on the new NCS Global Opportunity Mapper web portal to be launched in late 2023, with all project identifying information removed to ensure confidentiality.

All of the information you provide will be kept confidential. Only summary information with aggregated responses will be reported publicly. Your participation in this survey is voluntary and

you may withdraw at any time. There is minimal risk of your individual responses being revealed due to the measures we are taking to secure your information. The Lead Investigators for this project are Timm Kroeger and J.T. Erbaugh at The Nature Conservancy. You may reach them at tkroeger@tnc.org and james.erbaugh@tnc.org, respectively.

Thank you again for your support of this important effort! We greatly appreciate your expertise and contribution.
Please complete this survey by 31 December, 2023.

Sincerely,

The Nature Conservancy
African Forest Forum
Catholic Relief Services
Center for International Forestry Research / World Agroforestry Center
Conservation International
EcoAgriculture Partners
Eden Reforestation Projects
Foundation for Ecological Security
Heifer International
International Centre for Integrated Mountain Development
One Acre Fund
Regreening Africa
Wildlife Conservation Society
World Resources Institute
World Wildlife Fund

Page Break

After reading the description of this research, are you interested in participating in this survey?

◯ I am willing to participate

◯ I am not willing to participate

Page Break

Thank you for agreeing to participate.

The questions that follow ask for information about you, your organizational affiliation, and the **project** [2] for which you are entering constraints.

If you think there are additional people involved in your project with specific expertise on particular constraints your project faces, or with different perspectives on constraints, we would encourage each of them to complete the survey or, ideally, you and them to jointly complete a single survey.

You may share the survey with others who share expertise on the constraints your project faces by sending them this link:

https://tncva.qualtrics.com/jfe/form/SV_dbbsSQm8iSMPQtU

Name/s of respondent/s (First, Last):
Note: This question is optional. If more than one person complete this survey together, please separate all names with semicolons.

____

[2] An NCS "project" here is defined as 1) the direct implementation of one or several NCS by an entity or group of entities on a discrete site or sites (for example, a peatland rewetting and agroforestry project carried out by a local government with the assistance of a research entity); or 2) the provision, by others, of services that support the direct implementation of specific NCS practices across diverse sites by a discrete set of land owners or managers (for example, the provision of technical extension services that support the adoption of smallholder agroforestry in a particular geography).

Gender identity of respondent/s
Note: Please enter the number of respondents taking this survey that identify as the following genders

○ Women ____

○ Men ____

○ Non-binary ____

Nationality of respondent/s
Note: For groups of respondents with different nationalities, please separate each nationality with a semicolon.

____

Organizational affiliation of respondent/s
Note: If individuals from more than one organization complete this survey, or if an individual has multiple organizational affiliations, please separate each organization with a semicolon.

____

Position title of respondent/s
Note: If more than one person complete this survey together, please separate each position with a semicolon.

____

Page Break

Name of the **project** [3] for which you are entering constraints

Note 1: If you are working on multiple separate NCS projects, please complete this survey for one specific project. You may complete separate surveys for additional projects. If the activities carried out remain the same or similar but are carried out by successive projects, it is sufficient to complete one survey for all these projects. In this case, in the relevant fields below, please enter the different project names, the start date of the initial project, and, if applicable, the end date of the last project.

Note 2: Projects can face very different challenges when they implement activities in different administrative units (for example, countries, states or provinces). If your project operates across national boundaries, we strongly suggest completing a separate survey for each national context. In addition, if your project faces very different challenges in different areas within a single country, it might be useful to fill out separate surveys for these areas. However, we leave the geographic precision with which you want to report constraints for your project to your best judgment.

____

The year this project began (or will begin) to implement climate change mitigating activities
Note: If the start year is uncertain, please enter "uncertain."

____

The year this project ended or will end
Note: If the project does not have an end date, please enter "none."

____

What is the current total area of your project (in hectares, square kilometers, acres, or square miles)? If your project has ended, please enter the total area from the last year of its operation. If you provide services that support the direct implementation of NCS activities by others, enter the approximate total size of the area on which your services support NCS implementation.

[3] An NCS "project" here is defined as 1) the direct implementation of one or several NCS by an entity or group of entities on a discrete site or sites (for example, a peatland rewetting and agroforestry project carried out by a local government with the assistance of a research entity); or 2) the provision, by others, of services that support the direct implementation of specific NCS practices across diverse sites by a discrete set of land owners or managers (for example, the provision of technical extension services that support the adoption of smallholder agroforestry in a particular geography).

Note: If you do not measure your project in area, please leave this question blank

	Area Measurement	Unit			
		hectares	square kilometers	acres	square miles
Project Size		○	○	○	○

If you do not measure your project in terms of area, but there is another measurement for your project, please enter the measurement and units.

	Size Measurement	Unit
Project Size		

Page Break

Is this project pursuing carbon credits?

- ○ Yes
- ○ Not currently, but interest in exploring the possibility in the future
- ○ No
- ○ Unsure

Which of the following carbon markets or credit registries does the project target?

- ○ CAR (Climate Action Reserve)
- ○ VERRA (Verified Carbon Standard)
- ○ CDM (Clean Development Mechanism)
- ○ ACR (American Carbon Registry)
- ○ ICR (International Carbon Registry)
- ○ EU ETS (European Union Emissions Trading Scheme)
- ○ Plan Vivo
- ○ Other (please specify) ____
- ○ Unsure

Page Break

Country in which the **project** [4] is located
Note: If the project takes place in multiple countries, please list each country, separating them

[4] An NCS "project" here is defined as 1) the direct implementation of one or several NCS by an entity or group of entities on a discrete site or sites (for example, a peatland rewetting and agroforestry project carried out by a local government with the assistance of a research entity); or 2) the provision, by others, of services that support the direct implementation of specific NCS practices across diverse sites by a discrete set of land owners or managers (for example, the provision of technical extension services that support the adoption of smallholder agroforestry in a particular geography).

with a comma (",").

____

If you entered more than one country as the project location, please enter the country for which you are completing this survey.

____

Indicate where this project is implemented (by moving the marker)
Note: If the project takes place in multiple locations, please use the marker to identify the primary implementation location.

[Map widget]

____

Are there any other details about your project location that are important to know?
Note: Additional details you might include are the names of different states/provinces in which the project operates. Please only list the locations (states/provinces) in the country for which you are completing this survey.

____

____

____

____

____

Who owns the lands or waters on which this project operates (select all that apply):

- ☐ Government
- ☐ Private individuals
- ☐ Private companies
- ☐ Non-governmental organizations (NGOs)
- ☐ **Indigenous communities**[5]
- ☐ Other local communities
- ☐ Other (please specify)

____

[5] Indigenous Peoples are also referred to as Native Peoples, Aboriginal Peoples, First Peoples, or by locally-specific terms.

Who manages (*that is, tends, harvests or otherwise uses natural resources from)* the lands or waters on which this project operates (select all that apply):

- ☐ Government
- ☐ Private individuals
- ☐ Private companies
- ☐ Non-governmental organizations (NGOs)
- ☐ **Indigenous communities** [6]
- ☐ Other local communities
- ☐ Other (please specify)

____

Page Break

This NCS **project** implements or directly supports the implementation of *(select all that apply):*

Note: If you or your organization do not directly implement NCS on your own lands but rather provide services that directly support the implementation of NCS by others (for example, provision of technical extension services or of supplies or inputs for NCS), please select from the list below the NCS whose implementation your services support.

[6] Indigenous Peoples are also referred to as Native Peoples, Aboriginal Peoples, First Peoples, or by locally-specific terms.

- ☐ Agroforestry [7]
- ☐ Regenerative agriculture (other than Agroforestry) [8]
- ☐ Avoided forest conversion [9]
- ☐ Restoration of natural forests [10]
- ☐ Reforestation with commercial plantations [11]
- ☐ Climate-smart forestry [12]
- ☐ Avoided coastal wetland conversion [13]
- ☐ Coastal wetland restoration [14]
- ☐ Avoided grassland conversion [15]
- ☐ Grassland restoration [16]
- ☐ Avoided peatland conversion [17]
- ☐ Peatland restoration [18]
- ☐ Savanna management [19]
- ☐ Other (please specify):

____

[7] Integration of trees in crop or pasturelands, including tree intercropping, orchards, woodlots, and short-rotation energy tree crops.
[8] Activities that often are implemented to achieve soil or water conservation, or improved soil health or water quality: Biochar, changes in the type, timing, quantity, or targeting of fertilizer application, cover cropping, improved fallow or interplanting of legumes, establishment of riparian buffers, improved rice water management, reduced tillage, improved livestock density or rotation, legumes in pastures, grazing land fire management, or improved manure management.
[9] Avoided forest loss from commodity agriculture, shifting cultivation, or urban or infrastructure expansion.
[10] Active planting, assisted regrowth (for example: fencing, fertilization, or suppression of competition from non-tree or invasive species), or passive/natural recovery.

Activities related to agroforestry performed through this project:

- ☐ Trees in croplands (alley cropping, windbreaks) or trees in pasturelands
- ☐ Establishing orchards (fruit or nut trees)
- ☐ Establishing woodlots (treed area managed as a source of fuel, posts and other wood products)
- ☐ Establishing short-rotation tree crops for biomass energy
- ☐ Other (please specify)

____

Activities related to avoided forest conversion performed through this project:

- ○ Avoided conversion from agriculture
- ○ Avoided conversion from shifting cultivation
- ○ Avoided conversion from urban or infrastructure expansion
- ○ Other (please specify) ____

---

[11] Active planting of single species (monoculture) or multiple species (polyculture) plantations.
[12] Extended rotation lengths, reduced impact logging for climate mitigation (RIL-C), forestry set-asides (ceased logging), or enhanced growth.
[13] Saltmarsh, mangrove, or seagrass conservation.
[14] Re-establishment of saltmarshes, mangroves, or seagrass.
[15] Avoided loss of grasslands from commodity agriculture, shifting conservation, or urban or infrastructure expansion.
[16] Natural regeneration of grasslands or assisted planting of grasslands.
[17] Avoided peatland drainage or avoided peatland burning.
[18] Canal blocking or other forms of peatland re-wetting.
[19] Improved fire management and reduced wood harvest (including in prairies, woodlands, woody grasslands, shrublands, steppes, pampas, cerrado, tundra and other terrestrial habitats with tree cover of <25%).

NatReforSubPath Activities related to restoration of natural forests performed through this project:

- ☐ Active planting
- ☐ Assisted regrowth (for example: fencing, fertilization, or suppression of competition from non-tree or invasive species)
- ☐ Passive/natural recovery
- ☐ Other (please specify) ______________________________

Activities related to reforestation with commercial plantations through this project:

- ☐ Assisted planting of single tree species (monocultures)
- ☐ Assisted planting of multiple tree species (polycultures)
- ☐ Other (please specify) ______________________________

Activities related to climate-smart forestry performed through this project:

- ☐ Extended rotations
- ☐ Reduced impact logging for climate mitigation (RIL-C)
- ☐ Forestry set-asides (stop logging)
- ☐ Enhanced growth
- ☐ Other (please specify) ______________________________

Activities related to avoided coastal wetland conversion performed through this project:

- ☐ Avoided Saltmarsh conversion
- ☐ Avoided Mangrove conversion
- ☐ Avoided Seagrass conversion
- ☐ Other (please specify)

____

Activities related to coastal wetland restoration performed through this project:

- ☐ Saltmarsh restoration
- ☐ Mangrove restoration
- ☐ Seagrass restoration
- ☐ Other (please specify)

____

Activities related to avoided grassland conversion performed through this project:

- ☐ Avoided conversion from commodity agriculture
- ☐ Avoided conversion from shifting conservation
- ☐ Avoided conversion from urban or infrastructure expansion
- ☐ Other (please specify)

____

Activities related to grassland restoration performed through this project:

- ☐ Natural regeneration
- ☐ Assisted planting
- ☐ Other (please specify)

____

Activities related to avoided peatland conversion performed through this project:

- ☐ Avoided peatland drainage
- ☐ Avoided peatland burning
- ☐ Other (please specify)

____

Activities related to peatland restoration performed through this project:

- ☐ Canal blocking
- ☐ Other forms of peatland re-wetting
- ☐ Other (please specify)

____

Activities related to regenerative agriculture (other than agroforestry) performed through this project:

(Note: these are activities that often are implemented to achieve soil or water conservation, improved soil health, or improved water quality.)

- ☐ Application of biochar
- ☐ Changes in fertilizer application (type, rate, timing, location)
- ☐ Cover cropping
- ☐ Improved fallow or intercropping with legumes
- ☐ Establishment of riparian buffers
- ☐ Improved rice water management
- ☐ Reduced tillage
- ☐ Improved livestock density or rotation
- ☐ Planting legumes in pasture
- ☐ Improved manure management
- ☐ Improved fire management (grasslands, croplands)

Activities related to **savanna** management performed through this project:

- ☐ Fire management (for example: early dry season prescribed burning)
- ☐ Reduced wood harvest
- ☐ Other (please specify)

____

End of Block: Introduction

Start of Block: Module 1 Constraints Selection

The first part of this survey asks about constraints to implementing NCS projects. 'Constraints' refer to factors that limit the implementation of NCS or that make NCS implementation challenging.

When answering questions about constraints, please think about the barriers your project team (including any partners that help implement the project) faces that impact your ability to meet project objectives. These include barriers that make it challenging for individual land managers to implement NCS.

We suggest first reading through the full list of constraints, and then selecting all constraints that make it challenging for your team to achieve the full project objectives.

If you don’t see a constraint that is important to the implementation of your project, you may add that constraint in the "Other Constraint" text fields at the bottom of the constraints list.

Please complete this survey specifically for the project you identified above.

Page Break

Please indicate if your project faces constraints related to the following:

	Yes (Applies to my project)	No (Does not apply to my project)	Unsure
Planting stock or other materials for the NCS	◯	◯	◯
Labor (external or own) for the NCS	◯	◯	◯
Suitable land for the NCS	◯	◯	◯
Water for the NCS	◯	◯	◯
Land manager/operator [20] access to credit for NCS	◯	◯	◯
**Land manager/operator** access to other funding for NCS	◯	◯	◯
**Land manager/operator** insurance for NCS assets or outputs	◯	◯	◯
Project access to credit for NCS	◯	◯	◯
Project access to other funding for NCS	◯	◯	◯
Donor/Creditor relationships or reporting	◯	◯	◯
Markets for NCS outputs (for example, food or wood) produced by land managers/operators	◯	◯	◯
Markets for carbon sequestered by the NCS	◯	◯	◯
Markets [21] for ecosystem services (for example, water quality or flow regulation) or biodiversity provided by the NCS	◯	◯	◯
Prices for NCS outputs (for example, food or wood) produced by land managers/operators	◯	◯	◯

[20] Any actors or organizations that manage land, including private individuals, community groups, government organizations, or corporations.

[21] Including any type of payment for ecosystem services

Prices for carbon sequestered by the NCS	○	○	○
Prices for ecosystem services (for example, water quality or flow regulation) or biodiversity provided by the NCS	○	○	○
Greater profitability of alternative land uses (please specify alternative use)	○	○	○
Land manager/operator literacy, numeracy, or technological capabilities	○	○	○
Information about how to design or begin the NCS	○	○	○
Information about how to manage the NCS (for example, maintenance activities, monitoring activities, or other inputs and their timing)	○	○	○
Availability of technical advice for land managers/operators (for example, extension services)	○	○	○
Information about yields, inputs, or profits	○	○	○
Information about market access or prices	○	○	○
Information about on-site benefits of NCS (for example, soil fertility, shade for livestock, or income diversification)	○	○	○
Preferences for non-NCS land uses	○	○	○
Aversion to trying new land uses	○	○	○
Skepticism/disinterest toward NCS or lack of trust in NCS promoters	○	○	○
Social norms favoring non-NCS land uses	○	○	○

Concerns over negative equity impacts of NCS	○	○	○
Lack of opportunity to participate in or influence the implementation of NCS due to gender, race, ethnicity, or other dimensions of identity	○	○	○
Limited social learning or exchange networks for NCS	○	○	○
Difficulty identifying, engaging, or coordinating with relevant actors	○	○	○
Insecure or uncertain rights to manage or sell property	○	○	○
Insecure, uncertain, or lack of rights to use natural resources	○	○	○
Regulatory barriers to production, transport, or sale of NCS outputs (for example, need for permits or licensing)	○	○	○
Insecure or uncertain NCS benefit sharing	○	○	○
NCS-related corruption	○	○	○
Unclear laws and policies related to NCS outputs/markets	○	○	○
Lack of policy coordination (for example, between sectors or between different administrative units)	○	○	○
Uncertain enforcement of environmental laws	○	○	○
Weak monitoring and enforcement of NCS agreements	○	○	○
Violent conflict or perceived threat of violence	○	○	○

Lack of dispute resolution mechanisms	◯	◯	◯
Financial or other incentives for non-NCS (for example, subsidies, reduced taxes, access to credit or insurance)	◯	◯	◯
Politically influential interests favoring non-NCS	◯	◯	◯
Other Constraint 1 (please specify)	◯	◯	◯
Other Constraint 2 (please specify)	◯	◯	◯
Other Constraint 3 (please specify)	◯	◯	◯

Page Break

Please rank up to five of the most important constraints you have identified, with one (1) as the most important constraint, two (2) as the second-most important constraint, and so on. Five (5) will be the fifth-most important constraint.

For all constraints listed below that are not ranked one through five, please leave blank.

End of Block: Module 1 Constraints Selection

Start of Block: Module 2: Additional information on top constraints

Next, we ask more specific questions about the constraints you identified as most important.

For constraints related to "planting stock and other materials," indicate if the following constrain the implementation of your project.

	Yes (Applies to my project)	No (Does not apply to my project)	Unsure
**Limited or uncertain availability** of planting stock and other materials	○	○	○
**Limited or uncertain quality** of planting stock and other materials	○	○	○
**High or uncertain costs** of planting stock or other materials	○	○	○

Provide any additional information that might help us understand constraints related to "planting stock and other materials" for this project.

____

For constraints related to "labor (external or own) for NCS pathway," indicate if the following constrain the implementation of your project.

	Yes (Applies to my project)	No (Does not apply to my project)	Unsure
**Limited or uncertain availability** of labor (external or own)	○	○	○
**Limited or uncertain quality** of labor (external or own)	○	○	○
**High or uncertain costs** of labor (external or own)	○	○	○

Provide any additional information that might help us understand constraints related to "labor (external or own) for NCS pathway" for this project.

____

Page Break

For constraints related to "suitable land for NCS pathway," indicate if the following constrain the implementation of your project.

	Yes (Applies to my project)	No (Does not apply to my project)	Unsure
**Limited or uncertain availability** of land	○	○	○
**Limited or uncertain quality** of land	○	○	○
**High or uncertain cost** of land	○	○	○

Provide any additional information that might help us understand constraints related to "suitable land for NCS pathway" for this project.

____

Page Break

For constraints related to "water for NCS pathway," indicate if the following constrain the implementation of your project.

	Yes (Applies to my project)	No (Does not apply to my project)	Unsure
**Limited or uncertain availability** of water	○	○	○
**Limited or uncertain quality** of water	○	○	○
**High or uncertain cost** of water	○	○	○

Provide any additional information that might help us understand constraints related to "water for the NCS pathway" for this project.

____

Page Break

For constraints related to "land manager/operator access to credit for NCS pathway," indicate if the following constrain the implementation of your project.

	Yes (Applies to my project)	No (Does not apply to my project)	Unsure
**Limited or uncertain availability** of credit for land operators	○	○	○
**High or uncertain cost** of credit for land operators	○	○	○

Provide any additional information that might help us understand constraints related to "land manager/operator access to credit for NCS pathway" for this project.

____

Page Break

For constraints related to "land manager/operator access to other funding for NCS," indicate if the following constrain the implementation of your project.

	Yes (Applies to my project)	No (Does not apply to my project)	Unsure
**Limited or uncertain availability** of other funding for land operators	○	○	○
**High or uncertain cost** of other funding for land operators	○	○	○

Provide any additional information that might help us understand constraints related to "land manager/operator access to other funding for NCS" for this project.

____

Page Break

For constraints related to "land manager/operator insurance for NCS assets or outputs," indicate if the following constrain the implementation of your project.

	Yes (Applies to my project)	No (Does not apply to my project)	Unsure
**Limited or uncertain availability** of insurance for land operators	○	○	○
**High or uncertain cost** of insurance for land operators	○	○	○

Provide any additional information that might help us understand constraints related to "land manager/operator insurance for NCS assets or outputs" for this project.

____

Page Break

For constraints related to "project access to credit for NCS," indicate if the following constrain the implementation of your project.

	Yes (Applies to my project)	No (Does not apply to my project)	Unsure
**Limited or uncertain availability** of credit for the project	○	○	○
**High or uncertain cost** of credit for the project	○	○	○

Provide any additional information that might help us understand constraints related to "project access to credit for NCS" for this project.

____

Page Break

For constraints related to "project access to other funding for NCS," indicate if the following constrain the implementation of your project.

	Yes (Applies to my project)	No (Does not apply to my project)	Unsure
**Limited or uncertain availability** of other funding for the project	○	○	○
**High or uncertain cost** of other funding for the project	○	○	○

Provide any additional information that might help us understand constraints related to "project access to other funding for NCS" for this project.

____

Page Break

For constraints related to "donor/creditor relationships or reporting," indicate if the following constrain the implementation of your project.

	Yes (Applies to my project)	No (Does not apply to my project)	Unsure
**Limited or uncertain capacity** to manage donor/creditor relationships or reporting	○	○	○
**High or uncertain cost** to manage donor/creditor relationships or reporting	○	○	○

Provide any additional information that might help us understand constraints related to "donor/creditor relationships or reporting" for this project.

____

Page Break

For constraints related to "markets for NCS outputs (for example, food or wood) produced by the land manager/operator," indicate if the following constrain the implementation of your project.

	Yes (Applies to my project)	No (Does not apply to my project)	Unsure
**Limited or uncertain availability** of markets for outputs (for example, food or wood)	○	○	○
**High or uncertain costs** associated with accessing markets for project outputs (for example, this may be due to transport costs, market rules, or difficulty identifying buyers)	○	○	○

Provide any additional information that might help us understand constraints related to "markets for NCS outputs (for example, food or wood) produced by the land operator" for this project.

____

Page Break

For constraints related to "markets for carbon sequestered by the NCS," indicate if the following constrain the implementation of your project.

	Yes (Applies to my project)	No (Does not apply to my project)	Unsure
**Limited or uncertain availability** of markets for carbon	○	○	○
**High or uncertain costs** associated with accessing markets for carbon (for example, this may be due to transport costs, market rules, or difficulty identifying buyers)	○	○	○

Provide any additional information that might help us understand constraints related to "markets for carbon sequestered by the NCS" for this project.

____

Page Break

For constraints related to "markets for ecosystem services (for example, water quality or flow regulation) or biodiversity provided by the NCS," indicate if the following constrain the implementation of your project.

	Yes (Applies to my project)	No (Does not apply to my project)	Unsure
**Limited or uncertain availability** of markets for ecosystem services (for example, water quality or flow regulation) or biodiversity	○	○	○
**High or uncertain costs** associated with accessing markets for ecosystem services (for example, water quality or flow regulation) or biodiversity (for example, this may be due to transport costs, market rules, or difficulty identifying buyers)	○	○	○

Provide any additional information that might help us understand constraints related to "markets for ecosystem services (for example, water quality or flow regulation) or biodiversity provided by the NCS" for this project.

____

Page Break

For constraints related to "prices for NCS outputs (for example, food or wood) produced by the land managers/operator," indicate if the following constrains the implementation of your project.

	Yes (Applies to my project)	No (Does not apply to my project)	Unsure
**Low or uncertain prices** for outputs (for example, food or wood)	○	○	○

Provide any additional information that might help us understand constraints related to "prices for NCS outputs (for example, food or wood) produced by the land managers/operator" for this project.

____

Page Break

For constraints related to "prices for carbon sequestered by the NCS," indicate if the following constrains the implementation of your project.

	Yes (Applies to my project)	No (Does not apply to my project)	Unsure
**Low or uncertain prices** for carbon	○	○	○

Provide any additional information that might help us understand constraints related to "prices for carbon sequestered by the NCS" for this project.

____

Page Break

For constraints related to "prices for ecosystem services (for example, water quality or flow regulation) or biodiversity provided by the NCS," indicate if the following constrains the implementation of your project.

	Yes (Applies to my project)	No (Does not apply to my project)	Unsure
**Low or uncertain prices** for ecosystem services (for example, water quality or flow regulation) or biodiversity	○	○	○

Provide any additional information that might help us understand constraints related to "prices for ecosystem services (for example, water quality or flow regulation) or biodiversity provided by the NCS" for this project.

____

Page Break

For constraints related to "greater profitability of alternative land uses: *[respondent's earlier text entry]*" indicate if the following constrain the implementation of your project.

	Yes (Applies to my project)	No (Does not apply to my project)	Unsure
**Alternative land use(s) are more profitable** than NCS	○	○	○
**Uncertainty about whether alternative land uses are more profitable** than NCS	○	○	○

Provide any additional information that might help us understand constraints related to "greater profitability of alternative land uses: *[respondent's earlier text entry]*" for this project.

Page Break

For constraints related to "land manager/operator literacy, numeracy or technological capabilities," indicate if the following constrain the implementation of your project.

	Yes (Applies to my project)	No (Does not apply to my project)	Unsure
**Limited or uncertain availability** of land managers/operators with sufficient literacy, numeracy, or technological capabilities	○	○	○
**High or uncertain cost** associated with improving land operators' literacy, numeracy, or technological capabilities	○	○	○

Provide any additional information that might help us understand constraints related to "land manager/operator literacy, numeracy, or technological capabilities" for this project.

____

Page Break

Who primarily experiences constraints related to "information about how to design or begin the NCS?"

- ○ [Land managers/operators] primarily face the constraint
- ○ **Project staff/managers** primarily face the constraint
- ○ **Both land owners/managers and project staff/managers** face the constraint

For constraints related to "information about how to design or begin the NCS," indicate if the following constrain the implementation of your project.

	Yes (Applies to my project)	No (Does not apply to my project)	Unsure
**Limited or uncertain availability** of design and installation knowledge	○	○	○
**Limited or uncertain quality** of design and installation knowledge	○	○	○
**High or uncertain cost** associated with design and installation knowledge	○	○	○

Provide any additional information that might help us understand constraints related to "information about how to design or begin the NCS" for this project.

____

Page Break

Who primarily experiences constraints related to "information about how to manage the NCS (for example, maintenance activities, monitoring activities, or other inputs and their timing)?"

◯ Land managers/operators primarily face the constraint

◯ **Project staff/managers** primarily face the constraint

◯ **Both land owners/managers and project staff/managers** face the constraint

For constraints related to "information about how to manage the NCS (for example, maintenance activities, monitoring activities, or other inputs and their timing)," indicate if the following constrain the implementation of your project.

	Yes (Applies to my project)	No (Does not apply to my project)	Unsure
**Limited or uncertain availability** of operations and management knowledge	◯	◯	◯
**Limited or uncertain quality** of operations and management knowledge	◯	◯	◯
**High or uncertain cost** associated with operations and management knowledge	◯	◯	◯

Provide any additional information that might help us understand constraints related to "information about how to manage the NCS (for example, maintenance activities, monitoring activities, or other inputs and their timing)" for this project.

____

For constraints related to "availability of technical advice for land managers/operators (for example, extension services)," indicate if the following constrain the implementation of your project.

	Yes (Applies to my project)	No (Does not apply to my project)	Unsure
**Limited or uncertain availability** of technical advice or extension services	◯	◯	◯
**Limited or uncertainty quality** of technical advice or extension services	◯	◯	◯
**High or uncertain cost** of technical advice or extension services	◯	◯	◯

Provide any additional information that might help us understand constraints related to "availability of technical advice for land managers/operators (for example, extension services)" for this project.

____

Page Break

Who primarily experiences constraints related to "information about yields, inputs, and profits?"

◯ Land managers/operators primarily face the constraint

◯ **Project staff/managers** primarily face the constraint

◯ **Both land owners/managers and project staff/managers** face the constraint

For constraints related to "information about yields, inputs, and profits" for the outputs the project produces, indicate if the following constrain the implementation of your project.

	Yes (Applies to my project)	No (Does not apply to my project)	Unsure
**Limited or uncertain availability** of information on yields, inputs, and profits	◯	◯	◯
**Limited or uncertain quality** of information on yields, inputs, and profits	◯	◯	◯

Provide any additional information that might help us understand constraints related to "information about yields, inputs, and profits" for this project.

____

Page Break

Who primarily experiences constraints related to "information about market access or prices?"

- ◯ Land managers/operators primarily face the constraint
- ◯ **Project staff/managers** primarily face the constraint
- ◯ **Both land owners/managers and project staff/managers** face the constraint

For constraints related to "information about market access or prices," indicate if the following constrain the implementation of your project.

	Yes (Applies to my project)	No (Does not apply to my project)	Unsure
**Limited or uncertain availability** of Information on market access or prices	◯	◯	◯
**Limited or uncertain quality** of Information on market access or prices	◯	◯	◯

Provide any additional information that might help us understand constraints related to "information about market access or prices" for this project.

	Yes (Applies to my project)	No (Does not apply to my project)	Unsure
**Limited or uncertain availability** of Information on market access or prices	◯	◯	◯
**Limited or uncertain quality** of Information on market access or prices	◯	◯	◯

Who primarily experiences constraints related to "information about on-site co-benefits of NCS (for example, soil fertility, fodder or shade for livestock)?"

○ **Land owners/managers** primarily face the constraint

○ **Project staff/managers** primarily face the constraint

○ **Both land owners/managers and project staff/managers** face the constraint

For constraints related to "information about on-site co-benefits of NCS (for example, soil fertility, shade for livestock, or income diversification)," indicate if the following constrain the implementation of your project.

	Yes (Applies to my project)	No (Does not apply to my project)	Unsure
**Limited availability** of Information about on-site co-benefits	○	○	○
**Limited or uncertain quality** of Information about on-site co-benefits	○	○	○

Provide any additional information that might help us understand constraints related to "information about on-site co-benefits of NCS (for example, soil fertility, shade for livestock, or income diversification)" for this project.

____

Page Break

For constraints related to "preferences for non-NCS land uses," indicate if the following constrain the implementation of your project.

	Yes (Applies to my project)	No (Does not apply to my project)	Unsure
**Land manager/operator** preferences for non-NCS land uses	○	○	○
**Local community or government** preferences for non-NCS land uses	○	○	○
**District/County or State/Province government** preferences for non-NCS land uses	○	○	○
**National ministry or department** preferences for non-NCS land uses	○	○	○

Provide any additional information that might help us understand constraints related to "preferences for non-NCS land uses" for this project.

____

Page Break

For constraints related to "aversion to trying new land uses," indicate if the following constrain the implementation of your project.

	Yes (Applies to my project)	No (Does not apply to my project)	Unsure
**Land manager/operator** aversion to trying new land uses	○	○	○
**Local community or government** aversion to trying new land uses	○	○	○
**District/County or State/Province government** aversion to trying new land uses	○	○	○
**National ministry or department** aversion to trying new land uses	○	○	○

Provide any additional information that might help us understand constraints related to "aversion to trying new land uses" for this project.

____

Page Break

For constraints related to "skepticism toward NCS or a lack of trust in NCS promoters," indicate if the following constrain the implementation of your project.

	Yes (Applies to my project)	No (Does not apply to my project)	Unsure
**Land manager/operator** skepticism toward NCS or lack of trust toward NCS promoters	○	○	○
**Local community or government** skepticism toward NCS or lack of trust toward NCS promoters	○	○	○
**District/County or State/Province government** skepticism toward NCS or lack of trust toward NCS promoters	○	○	○
**National ministry or department** skepticism toward NCS or lack of trust toward NCS promoters	○	○	○

Provide any additional information that might help us understand constraints related to "skepticism toward NCS or a lack of trust in NCS promoters" for this project.

____

Page Break

For constraints related to "social norms favoring non-NCS land uses," indicate if the following constrain the implementation of your project

	Yes (Applies to my project)	No (Does not apply to my project)	Unsure
**Land manager/operator** favor non-NCS due to social norms	○	○	○
**Local community or government** favor non-NCS due to social norms	○	○	○
**District/County or State/Province government** favors non-NCS due to social norms	○	○	○
**National ministry or department** favors non-NCS due to social norms	○	○	○

3.28.01 Provide any additional information that might help us understand constraints related to "social norms favoring non-NCS uses" for this project.

____

Page Break

For constraints related to "concerns over negative equity impacts of NCS," indicate if the following constrain the implementation of your project.

	Yes (Applies to my project)	No (Does not apply to my project)	Unsure
**Land manager/operator** concerns over negative equity impacts	○	○	○
**Local community or government** concerns over negative equity impacts	○	○	○
**District/County or State/Province government** concerns over negative equity impacts	○	○	○
**National ministry or department** concerns over negative equity impacts	○	○	○

Provide any additional information that might help us understand constraints related to "concerns over negative equity impacts of NCS" for this project.

____

Page Break

For constraints related to "lack of opportunity to participate in or influence the implementation of NCS due to gender, race, ethnicity, or other dimensions of identity," indicate if the following constrain the implementation of your project.

	Yes (Applies to my project)	No (Does not apply to my project)	Unsure
**Gender inequities** define the lack of opportunity to participate in or influence the implementation of NCS	○	○	○
**Racial inequities** define the lack of opportunity to participate in or influence the implementation of NCS	○	○	○
**Ethnic inequities** define the lack of opportunity to participate in or influence the implementation of NCS	○	○	○
**Other inequities** define the lack of opportunity to participate in or influence the implementation of NCS (please specify)	○	○	○

Provide any additional information that might help us understand constraints related to "lack of opportunity to participate in or influence the implementation of NCS due to gender, race, ethnicity, or other dimensions of identity" for this project.

____

For constraints related to "limited social learning or exchange networks for NCS," indicate if the following constrain the implementation of your project.

	Yes (Applies to my project)	No (Does not apply to my project)	Unsure
**Land manager/operator** limited access to social learning or exchange networks	○	○	○
**Local community or government** access to social learning or exchange networks	○	○	○
**District/County or State/Province** officials lacking access to social learning or exchange networks	○	○	○
**National ministry or department** officials lacking access to social learning or exchange networks	○	○	○

Provide any additional information that might help us understand constraints related to "limited social learning or exchange networks for NCS" for this project.

____

Page Break

For constraints related to "difficulty identifying, engaging, or coordinating with relevant actors," indicate if the following constrain the implementation of your project.

	Yes (Applies to my project)	No (Does not apply to my project)	Unsure
It is difficult to identify, engage, or coordinate with **government actors or organizations**	○	○	○
It is difficult to identify, engage, or coordinate with **Indigenous individuals or communities**	○	○	○
It is difficult to identify, engage, or coordinate with **non-indigenous individuals or communities**	○	○	○
It is difficult to identify, engage, or coordinate with **NGOs**	○	○	○
It is difficult to identify, engage, or coordinate with **other individuals or communities** (please specify)	○	○	○

Provide any additional information that might help us understand constraints related to "difficulty identifying, engaging, or coordinating with relevant actors" for this project.

____

Page Break

For constraints related to "insecure or uncertain rights to manage or sell property," indicate if the following constrain the implementation of your project.

	Yes (Applies to my project)	No (Does not apply to my project)	Unsure
Insecure or uncertain rights to manage or sell property owned/managed by **land managers/operators**	○	○	○
Insecure or uncertain rights to manage or sell property owned/managed by **local communities**	○	○	○
Insecure or uncertain rights to manage property owned by **Districts/Counties or States/Provinces**	○	○	○
Insecure or uncertain rights to manage property owned by **National ministries or departments**	○	○	○

Provide any additional information that might help us understand constraints related to "insecure or uncertain rights to manage or sell property" for this project.

____

Page Break

For constraints related to "insecure, uncertain, or lack of rights to use natural resources," indicate if the following constrain the implementation of your project.

	Yes (Applies to my project)	No (Does not apply to my project)	Unsure
Insecure, uncertain, or lack of rights to use natural resources owned/managed by **land managers/operators**	○	○	○
Insecure, uncertain, or lack of rights to use natural resources owned/managed by **local communities**	○	○	○
Insecure, uncertain, or lack of rights to use natural resources owned/managed by **Districts/Counties or States/Provinces**	○	○	○
Insecure, uncertain, or lack of rights to use natural resources owned/managed by **National ministries or departments**	○	○	○

Provide any additional information that might help us understand constraints related to "insecure, uncertain, or lack of rights to use natural resources " for this project.

____

Page Break

For constraints related to "regulatory barriers to production, transport, or sale of NCS outputs (for example, permits or licensing)," indicate if the following constrain the implementation of your project.

	Yes (Applies to my project)	No (Does not apply to my project)	Unsure
Regulatory barriers to production, transport or sale introduced by **local communities or government**	○	○	○
**District/County or State-Province**-level regulatory barriers to production, transport or sale of NCS	○	○	○
Regulatory barriers to production, transport or sale of NCS introduced by **National ministries or departments**	○	○	○

Provide any additional information that might help us understand constraints related to "regulatory barriers to production, transport, or sale of NCS outputs (for example, permits or licensing)" for this project.

____

Page Break

For constraints related to "insecure or uncertain NCS benefit sharing," indicate if the following constrain the implementation of your project.

	Yes (Applies to my project)	No (Does not apply to my project)	Unsure
Insecure or uncertain benefit sharing among **land managers/operators**	○	○	○
Insecure or uncertain benefit sharing among **local communities**	○	○	○
Insecure or uncertain benefit sharing among **Districts/Counties or States/Provinces**	○	○	○
Insecure or uncertain benefit sharing among **National ministries or departments**	○	○	○

Provide any additional information that might help us understand constraints related to "unsecure or uncertain NCS benefit sharing" for this project.

____

Page Break

For constraints related to "NCS-related corruption," indicate if the following constrain the implementation of your project.

	Yes (Applies to my project)	No (Does not apply to my project)	Unsure
NCS-related corruption among **land managers/operators**	○	○	○
NCS-related corruption among **local communities**	○	○	○
NCS-related corruption among **Districts/Counties or States/Provinces** government personnel	○	○	○
NCS-related corruption among **National ministry or department** personnel	○	○	○

Provide any additional information that might help us understand constraints related to "NCS-related corruption" for this project.

____

Page Break

For constraints related to "unclear laws and policies related to NCS outputs/markets," indicate if the following constrain the implementation of your project.

	Yes (Applies to my project)	No (Does not apply to my project)	Unsure
Unclear laws or policies introduced by **local communities or governments**	○	○	○
Unclear laws or policies introduced by introduced by **Districts/Counties or States/Provinces**	○	○	○
Unclear laws or policies introduced by introduced by **National organizations, agencies, or departments**	○	○	○

Provide any additional information that might help us understand constraints related to "unclear laws and policies related to NCS outputs/markets" for this project.

____

Page Break

For constraints related to "lack of policy coordination (for example, between sectors or between different administrative units)," indicate if the following constrain the implementation of your project.

	Yes (Applies to my project)	No (Does not apply to my project)	Unsure
Lack of policy coordination among **local communities or governments**	○	○	○
Lack of policy coordination among **districts/counties or States/Provinces**	○	○	○
Lack of policy coordination among **national organizations, agencies, or departments**	○	○	○

Provide any additional information that might help us understand constraints related to "lack of policy coordination (for example, between sectors or between different administrative units)" for this project.

____

Page Break

For constraints related to "uncertain enforcement of environmental laws," indicate if the following constraint the implementation of your project.

	Yes (Applies to my project)	No (Does not apply to my project)	Unsure
Uncertain enforcement of environmental rules by **land managers/operators**	○	○	○
Uncertain enforcement of environmental rules by **local communities or governments**	○	○	○
Uncertain enforcement of environmental rules by **Districts/Counties or States/Provinces**	○	○	○
Uncertain enforcement of environmental rules by **National organizations, agencies, or departments**	○	○	○

Provide any additional information that might help us understand constraints related to "uncertain enforcement of environmental laws" for this project.

____

Page Break

For constraints related to "weak monitoring and enforcement of NCS agreements," indicate if the following constrain the implementation of your project.

	Yes (Applies to my project)	No (Does not apply to my project)	Unsure
Weak monitoring and enforcement of NCS agreements by **land managers/operators**	○	○	○
Weak monitoring and enforcement of NCS agreements by **local communities or governments**	○	○	○
Weak monitoring and enforcement of NCS agreements by **Districts/Counties or States/Provinces**	○	○	○
Weak monitoring and enforcement of NCS agreements by **National organizations, agencies, or departments**	○	○	○

Provide any additional information that might help us understand constraints related to "weak monitoring and enforcement of NCS agreements" for this project.

____

Page Break

For constraints related to "violent conflict or the perceived threat of violence," indicate if the following constrain the implementation of your project.

	Yes (Applies to my project)	No (Does not apply to my project)	Unsure
Violent conflict or the perceived threat of violence between **non-government individuals or groups**	◯	◯	◯
Violent conflict or the perceived threat of violence conflict between **government forces**	◯	◯	◯
Violent conflict or the perceived threat of violence conflicts between **non-government individuals or groups and government forces**	◯	◯	◯

Provide any additional information that might help us understand constraints related to "violent conflict or the perceived threat of violence" for this project.

____

Page Break

For constraints related to "lack of dispute resolution mechanisms," indicate if the following constrain the implementation of your project.

	Yes (Applies to my project)	No (Does not apply to my project)	Unsure
Lack of dispute resolution mechanisms for **land managers/operators**	○	○	○
Lack of dispute resolution mechanisms for **local communities or governments**	○	○	○
Lack of dispute resolution mechanisms for **Districts/Counties or States/Provinces**	○	○	○
Lack of dispute resolution mechanisms for **National forces or organizations**	○	○	○

Provide any additional information that might help us understand constraints related to "lack of dispute resolution mechanisms," for this project.

____

Page Break

For constraints related to "financial or other incentives for non-NCS (for example, subsidies, reduced taxes, access to credit or insurance)," indicate if the following constrain the implementation of your project.

	Yes (Applies to my project)	No (Does not apply to my project)	Unsure
Financial of other incentives introduced by **land managers/operators**	○	○	○
Financial or other incentives introduced by **local communities or governments**	○	○	○
Financial or other incentives introduced by **districts/counties or states/provinces**	○	○	○
Financial or other incentives introduced by **national organizations, agencies, or departments**	○	○	○

Provide any additional information that might help us understand constraints related to "financial or other incentives for non-NCS (for example, subsidies, reduced taxes, access to credit or insurance)" for this project.

____

Page Break

For constraints related to "politically influential interests favoring non-NCS," indicate if the following constrain the implementation of your project.

	Yes (Applies to my project)	No (Does not apply to my project)	Unsure
Politically influential interests advocating against NCS with **land managers/operators**	○	○	○
Politically influential interests advocating against NCS with **local communities or governments**	○	○	○
Politically influential interests advocating against NCS with **Districts/Counties or States/Provinces**	○	○	○
Politically influential interests advocating against NCS with **National organizations, agencies, or departments**	○	○	○

Provide any additional information that might help us understand constraints related to "politically influential interests favoring non-NCS" for this project.

____

Page Break

For constraints related to "other constraint 1: *[respondent's write-in constraint 1 in constraints list]*" indicate if the following statements apply to your project.

	Yes (Applies to my project)	No (Does not apply to my project)	Unsure
Other constraint 1: *[respondent's write-in constraint 1]* affects **local communities or governments** to constrain project implementation	○	○	○
Other constraint 1: *[respondent's write-in constraint 1]* affects **Districts/Counties or States/Provinces** to constrain project implementation	○	○	○
Other constraint 1: *[respondent's write-in constraint 1]* affects **National organizations, agencies, or departments** to constrain project implementation	○	○	○
**Limited or uncertain availability** of other constraint 1: *[respondent's write-in constraint 1]*	○	○	○
**Limited or uncertain quality** of other constraint 1: *[respondent's write-in constraint 1]*	○	○	○
**High or uncertain price** of other constraint 1: *[respondent's write-in constraint 1]*	○	○	○

Provide any additional information that might help us understand constraints related to ""other constraint 1: *[respondent's write-in constraint 1]*" for this project.

____

For constraints related to "other constraint 2: *[respondent's write-in constraint 2]*" indicate if the following statements apply to your project.

	Yes (Applies to my project)	No (Does not apply to my project)	Unsure
Other constraint 2: *[respondent's write-in constraint 2]* affects **local communities or governments** to constrain project implementation	○	○	○
Other constraint 2: *[respondent's write-in constraint 2]* affects **Districts/Counties or States/Provinces** to constrain project implementation	○	○	○
Other constraint 2: *[respondent's write-in constraint 2]* affects **National organizations, agencies, or departments** to constrain project implementation	○	○	○
**Limited or uncertain availability** of other constraint 2: *[respondent's write-in constraint 2]*	○	○	○
**Limited or uncertain quality** of other constraint 2: *[respondent's write-in constraint 2]*	○	○	○
**High or uncertain price** of other constraint 2: *[respondent's write-in constraint 2]*	○	○	○

Provide any additional information that might help us understand constraints related to ""other constraint 2: *[respondent's write-in constraint 2]*" for this project.

____

Page Break

For constraints related to "other constraint 3: *[respondent's write-in constraint 3]*" indicate if the following statements apply to your project.

	Yes (Applies to my project)	No (Does not apply to my project)	Unsure
Other constraint 3: *[respondent's write-in constraint 3]* affects **local communities or governments** to constrain project implementation	○	○	○
Other constraint 3: *[respondent's write-in constraint 3]* affects **Districts/Counties or States/Provinces** to constrain project implementation	○	○	○
Other constraint 3: *[respondent's write-in constraint 3]* affects **National organizations, agencies, or departments** to constrain project implementation	○	○	○
**Limited or uncertain availability** of other constraint 3: *[respondent's write-in constraint 3]*	○	○	○
**Limited or uncertain quality** of other constraint 3: *[respondent's write-in constraint 3]*	○	○	○
**High or uncertain price** of other constraint 3: *[respondent's write-in constraint 3]*	○	○	○

Provide any additional information that might help us understand constraints related to "other constraint 3: *[respondent's write-in constraint 3]*" for this project.

____

End of Block: Module 2: Additional information on top constraints

---

Start of Block: Module 3: Solutions to Constraints

The following set of questions will ask about real or potential solutions to the constraints you identified as most relevant to your project.

Page Break

Thinking only about your project, how might the constraint related to "planting stock or other materials for NCS pathway" be overcome or at least mitigated?

Please provide your answer in one to three sentences and identify the actors (who) and the actions (what) that you believe might address this constraint.

____

Are any of these actions that you identified currently being taken, or are efforts underway to take these actions?

- ◯ Yes
- ◯ No
- ◯ Unsure
- ◯ Prefer not to answer

Do you have any information on the costs associated with this solution or these solutions?
Note: We do not ask about any cost information in this survey.

○ Yes

○ No

○ Unsure

○ Prefer not to answer

Page Break

Thinking only about your project, how might the constraint related to "labor (external or own) for NCS pathway," be overcome or at least mitigated?

Please provide your answer in one to three sentences and identify the actors (who) and the activities (what) that you believe might address this constraint.

____

Are any of these actions that you identified currently being taken, or are efforts underway to take these actions?

○ Yes

○ No

○ Unsure

○ Prefer not to answer

Do you have any information on the costs associated with this solution or these solutions?

Note: We do not ask about any cost information in this survey.

- ◯ Yes
- ◯ No
- ◯ Unsure
- ◯ Prefer not to answer

Page Break

Thinking only about your project, how might the constraint related to "suitable land for NCS pathway" be overcome or at least mitigated?

Please provide your answer in one to three sentences and identify the actors (who) and the activities (what) that you believe might address this constraint.

____

Are any of these actions that you identified currently being taken, or are efforts underway to take these actions?

- ◯ Yes
- ◯ No
- ◯ Unsure
- ◯ Prefer not to answer

Do you have any information on the costs associated with this solution or these solutions?

Note: We do not ask about any cost information in this survey.

- ○ Yes
- ○ No
- ○ Unsure
- ○ Prefer not to answer

Page Break

Thinking only about your project, how might the constraint related to "water for NCS pathway" be overcome or at least mitigated?

Please provide your answer in one to three sentences and identify the actors (who) and the activities (what) that you believe might address this constraint.

____

Are any of these actions that you identified currently being taken, or are efforts underway to take these actions?

- ○ Yes
- ○ No
- ○ Unsure
- ○ Prefer not to answer

Do you have any information on the costs associated with this solution or these solutions?

Note: We do not ask about any cost information in this survey.

○ Yes

○ No

○ Unsure

○ Prefer not to answer

Page Break

Thinking only about your project, how might the constraint related to "land manager/operator access to credit for NCS" be overcome or at least mitigated?

Please provide your answer in one to three sentences and identify the actors (who) and the activities (what) that you believe might address this constraint.

____

Are any of these actions that you identified currently being taken, or are efforts underway to take these actions?

○ Yes

○ No

○ Unsure

○ Prefer not to answer

Do you have any information on the costs associated with this solution or these solutions?

Note: We do not ask about any cost information in this survey.

- ◯ Yes
- ◯ No
- ◯ Unsure
- ◯ Prefer not to answer

Page Break

Thinking only about your project, how might the constraint related to "land manager/operator access to other funding for NCS" be overcome or at least mitigated?

Please provide your answer in one to three sentences and identify the actors (who) and the activities (what) that you believe might address this constraint.

____

Are any of these actions that you identified currently being taken, or are efforts underway to take these actions?

- Yes
- No
- Unsure
- Prefer not to answer

Do you have any information on the costs associated with this solution or these solutions?

Note: We do not ask about any cost information in this survey.

- Yes
- No
- Unsure
- Prefer not to answer

Page Break

Thinking only about your project, how might the constraint related to "land manager/operator insurance for NCS assets or outputs" be overcome or at least mitigated?

Please provide your answer in one to three sentences and identify the actors (who) and the activities (what) that you believe might address this constraint.

____

Are any of these actions that you identified currently being taken, or are efforts underway to take these actions?

◯ Yes

◯ No

◯ Unsure

◯ Prefer not to answer

Do you have any information on the costs associated with this solution or these solutions?

Note: We do not ask about any cost information in this survey.

◯ Yes

◯ No

◯ Unsure

◯ Prefer not to answer

Page Break

Thinking only about your project, how might the constraint related to "project access to credit for NCS" be overcome or at least mitigated?

Please provide your answer in one to three sentences and identify the actors (who) and the activities (what) that you believe might address this constraint.

____

Are any of these actions that you identified currently being taken, or are efforts underway to take these actions?

◯ Yes

◯ No

◯ Unsure

◯ Prefer not to answer

Do you have any information on the costs associated with this solution or these solutions?

Note: We do not ask about any cost information in this survey.

- ◯ Yes
- ◯ No
- ◯ Unsure
- ◯ Prefer not to answer

Page Break

Thinking only about your project, how might the constraint related to "project access to other funding for NCS" be overcome or at least mitigated?

Please provide your answer in one to three sentences and identify the actors (who) and the activities (what) that you believe might address this constraint.

____

Are any of these actions that you identified currently being taken, or are efforts underway to take these actions?

- ◯ Yes
- ◯ No
- ◯ Unsure
- ◯ Prefer not to answer

Do you have any information on the costs associated with this solution or these solutions?

Note: We do not ask about any cost information in this survey.

- ◯ Yes
- ◯ No
- ◯ Unsure
- ◯ Prefer not to answer

Page Break

Thinking only about your project, how might the constraint related to "donor/creditor relationships or reporting" be overcome or at least mitigated?

Please provide your answer in one to three sentences and identify the actors (who) and the activities (what) that you believe might address this constraint.

____

Are any of these actions that you identified currently being taken, or are efforts underway to take these actions?

- ◯ Yes
- ◯ No
- ◯ Unsure
- ◯ Prefer not to answer

Do you have any information on the costs associated with this solution or these solutions?

Note: We do not ask about any cost information in this survey.

○ Yes

○ No

○ Unsure

○ Prefer not to answer

Page Break

Thinking only about your project, how might the constraint related to "markets for NCS outputs (for example, food or wood) produced by the land manager/operator" be overcome or at least mitigated?

Please provide your answer in one to three sentences and identify the actors (who) and the activities (what) that you believe might address this constraint.

____

Are any of these actions that you identified currently being taken, or are efforts underway to take these actions?

○ Yes

○ No

○ Unsure

○ Prefer not to answer

Do you have any information on the costs associated with this solution or these solutions?

Note: We do not ask about any cost information in this survey.

- ◯ Yes
- ◯ No
- ◯ Unsure
- ◯ Prefer not to answer

Page Break

Thinking only about your project, how might the constraint related to "markets for carbon sequestered by the NCS" be overcome or at least mitigated?

Please provide your answer in one to three sentences and identify the actors (who) and the activities (what) that you believe might address this constraint.

____

Are any of these actions that you identified currently being taken, or are efforts underway to take these actions?

- ◯ Yes
- ◯ No
- ◯ Unsure
- ◯ Prefer not to answer

Do you have any information on the costs associated with this solution or these solutions?

Note: We do not ask about any cost information in this survey.

◯ Yes

◯ No

◯ Unsure

◯ Prefer not to answer

Page Break

Thinking only about your project, how might the constraint related to "markets for ecosystem services (for example, water quality or flow regulation) or biodiversity provided by the NCS" be overcome or at least mitigated?

Please provide your answer in one to three sentences and identify the actors (who) and the activities (what) that you believe might address this constraint.

____

Are any of these actions that you identified currently being taken, or are efforts underway to take these actions?

◯ Yes

◯ No

◯ Unsure

◯ Prefer not to answer

Do you have any information on the costs associated with this solution or these solutions?

Note: We do not ask about any cost information in this survey.

- ◯ Yes
- ◯ No
- ◯ Unsure
- ◯ Prefer not to answer

Page Break

Thinking only about your project, how might the constraint related to "prices for NCS outputs (for example, food or wood) produced by the land manager/operator" be overcome or at least mitigated?

Please provide your answer in one to three sentences and identify the actors (who) and the activities (what) that you believe might address this constraint.

____

Are any of these actions that you identified currently being taken, or are efforts underway to take these actions?

- ◯ Yes
- ◯ No
- ◯ Unsure
- ◯ Prefer not to answer

Do you have any information on the costs associated with this solution or these solutions?

Note: We do not ask about any cost information in this survey.

- ○ Yes
- ○ No
- ○ Unsure
- ○ Prefer not to answer

Page Break

Thinking only about your project, how might the constraint related to "prices for carbon sequestered by the NCS" be overcome or at least mitigated?

Please provide your answer in one to three sentences and identify the actors (who) and the activities (what) that you believe might address this constraint.

____

Are any of these actions that you identified currently being taken, or are efforts underway to take these actions?

- ○ Yes
- ○ No
- ○ Unsure
- ○ Prefer not to answer

Do you have any information on the costs associated with this solution or these solutions?

Note: We do not ask about any cost information in this survey.

○ Yes

○ No

○ Unsure

○ Prefer not to answer

Page Break

Thinking only about your project, how might the constraint related to "prices for ecosystem services (for example, water quality or flow regulation) or biodiversity provided by the NCS" be overcome or at least mitigated?

Please provide your answer in one to three sentences and identify the actors (who) and the activities (what) that you believe might address this constraint.

____

Are any of these actions that you identified currently being taken, or are efforts underway to take these actions?

○ Yes

○ No

○ Unsure

○ Prefer not to answer

Do you have any information on the costs associated with this solution or these solutions?

Note: We do not ask about any cost information in this survey.

○ Yes

○ No

○ Unsure

○ Prefer not to answer

Page Break

Thinking only about your project, how might the constraint related to "greater profitability of alternative land uses: *[respondent-identified land use]*" be overcome or at least mitigated?

Please provide your answer in one to three sentences and identify the actors (who) and the activities (what) that you believe might address this constraint.

____

Are any of these actions that you identified currently being taken, or are efforts underway to take these actions?

○ Yes

○ No

○ Unsure

○ Prefer not to answer

Do you have any information on the costs associated with this solution or these solutions?

Note: We do not ask about any cost information in this survey.

○ Yes

○ No

○ Unsure

○ Prefer not to answer

Page Break

Thinking only about your project, how might the constraint related to "land manager/operator literacy, numeracy or technological capabilities" be overcome or at least mitigated?

Please provide your answer in one to three sentences and identify the actors (who) and the activities (what) that you believe might address this constraint.

____

Are any of these actions that you identified currently being taken, or are efforts underway to take these actions?

○ Yes

○ No

○ Unsure

○ Prefer not to answer

Do you have any information on the costs associated with this solution or these solutions?

Note: We do not ask about any cost information in this survey.

- ◯ Yes
- ◯ No
- ◯ Unsure
- ◯ Prefer not to answer

Page Break

Thinking only about your project, how might the constraint related to "information about how to design or begin the NCS" be overcome or at least mitigated?

Please provide your answer in one to three sentences and identify the actors (who) and the activities (what) that you believe might address this constraint.

____

Are any of these actions that you identified currently being taken, or are efforts underway to take these actions?

- ◯ Yes
- ◯ No
- ◯ Unsure
- ◯ Prefer not to answer

Do you have any information on the costs associated with this solution or these solutions?

Note: We do not ask about any cost information in this survey.

○ Yes

○ No

○ Unsure

○ Prefer not to answer

Page Break

Thinking only about your project, how might the constraint related to "information about how to manage the NCS (for example, maintenance activities, monitoring activities, or other inputs and their timing)" be overcome or at least mitigated?

Please provide your answer in one to three sentences and identify the actors (who) and the activities (what) that you believe might address this constraint.

____

Are any of these actions that you identified currently being taken, or are efforts underway to take these actions?

○ Yes

○ No

○ Unsure

○ Prefer not to answer

Do you have any information on the costs associated with this solution or these solutions?

Note: We do not ask about any cost information in this survey.

- Yes
- No
- Unsure
- Prefer not to answer

Page Break

Thinking only about your project, how might the constraint related to "availability of technical advice for land managers/operators (for example, extension services)" be overcome or at least mitigated?

Please provide your answer in one to three sentences and identify the actors (who) and the activities (what) that you believe might address this constraint.

____

Are any of these actions that you identified currently being taken, or are efforts underway to take these actions?

- Yes
- No
- Unsure
- Prefer not to answer

Do you have any information on the costs associated with this solution or these solutions?

Note: We do not ask about any cost information in this survey.

- ○ Yes
- ○ No
- ○ Unsure
- ○ Prefer not to answer

Page Break

Thinking only about your project, how might the constraint related to "information about yields, inputs, and profits" be overcome or at least mitigated?

Please provide your answer in one to three sentences and identify the actors (who) and the activities (what) that you believe might address this constraint.

____

Are any of these actions that you identified currently being taken, or are efforts underway to take these actions?

- ○ Yes
- ○ No
- ○ Unsure
- ○ Prefer not to answer

Do you have any information on the costs associated with this solution or these solutions?

Note: We do not ask about any cost information in this survey.

- ○ Yes
- ○ No
- ○ Unsure
- ○ Prefer not to answer

Page Break

Thinking only about your project, how might constraints related to "information about market access or prices" be overcome or at least mitigated?

Please provide your answer in one to three sentences and identify the actors (who) and the activities (what) that you believe might address this constraint.

____

Are any of these actions that you identified currently being taken, or are efforts underway to take these actions?

- ○ Yes
- ○ No
- ○ Unsure
- ○ Prefer not to answer

Do you have any information on the costs associated with this solution or these solutions?

Note: We do not ask about any cost information in this survey.

○ Yes

○ No

○ Unsure

○ Prefer not to answer

Page Break

Thinking only about your project, how might the constraint related to "information about on-site co-benefits of NCS (for example, soil fertility, shade for livestock, or income diversification)" be overcome or at least mitigated?

Please provide your answer in one to three sentences and identify the actors (who) and the activities (what) that you believe might address this constraint.

____

Are any of these actions that you identified currently being taken, or are efforts underway to take these actions?

○ Yes

○ No

○ Unsure

○ Prefer not to answer

Do you have any information on the costs associated with this solution or these solutions?

Note: We do not ask about any cost information in this survey.

◯ Yes

◯ No

◯ Unsure

◯ Prefer not to answer

Page Break

Thinking only about your project, how might the constraint related to "preferences for non-NCS land uses," be overcome or at least mitigated?

Please provide your answer in one to three sentences and identify the actors (who) and the activities (what) that you believe might address this constraint.

____

Are any of these actions that you identified currently being taken, or are efforts underway to take these actions?

◯ Yes

◯ No

◯ Unsure

◯ Prefer not to answer

Do you have any information on the costs associated with this solution or these solutions?

Note: We do not ask about any cost information in this survey.

◯ Yes

◯ No

◯ Unsure

◯ Prefer not to answer

Page Break

Thinking only about your project, how might the constraint related to "aversion to trying new land uses" be overcome or at least mitigated?

Please provide your answer in one to three sentences and identify the actors (who) and the activities (what) that you believe might address this constraint.

____

Page Break

Are any of these actions that you identified currently being taken, or are efforts underway to take these actions?

◯ Yes

◯ No

◯ Unsure

◯ Prefer not to answer

Do you have any information on the costs associated with this solution or these solutions?

Note: We do not ask about any cost information in this survey.

- ○ Yes
- ○ No
- ○ Unsure
- ○ Prefer not to answer

Page Break

Thinking only about your project, how might the constraint related to "skepticism toward NCS or a lack of trust in NCS promoters" be overcome or at least mitigated?

Please provide your answer in one to three sentences and identify the actors (who) and the activities (what) that you believe might address this constraint.

____

Are any of these actions that you identified currently being taken, or are efforts underway to take these actions?

- ○ Yes
- ○ No
- ○ Unsure
- ○ Prefer not to answer

Do you have any information on the costs associated with this solution or these solutions?

Note: We do not ask about any cost information in this survey.

○ Yes

○ No

○ Unsure

○ Prefer not to answer

Page Break

Thinking only about your project, how the constraint related to "social norms favoring non-NCS land uses" be overcome or at least mitigated?

Please provide your answer in one to three sentences and identify the actors (who) and the activities (what) that you believe might address this constraint.

____

Are any of these actions that you identified currently being taken, or are efforts underway to take these actions?

○ Yes

○ No

○ Unsure

○ Prefer not to answer

Do you have any information on the costs associated with this solution or these solutions?

Note: We do not ask about any cost information in this survey.

- ◯ Yes
- ◯ No
- ◯ Unsure
- ◯ Prefer not to answer

Page Break

Thinking only about your project, how might the constraint related to "concerns over negative equity impacts of NCS" be overcome or at least mitigated?

Please provide your answer in one to three sentences and identify the actors (who) and the activities (what) that you believe might address this constraint.

____

Are any of these actions that you identified currently being taken, or are efforts underway to take these actions?

- ◯ Yes
- ◯ No
- ◯ Unsure
- ◯ Prefer not to answer

Do you have any information on the costs associated with this solution or these solutions?

Note: We do not ask about any cost information in this survey.

○ Yes

○ No

○ Unsure

○ Prefer not to answer

Page Break

Thinking only about your project, how might the constraint related to "lack of opportunity to participate in or influence the implementation of NCS due to gender, race, ethnicity, or other dimensions of identity" be overcome or at least mitigated?

Please provide your answer in one to three sentences and identify the actors (who) and the activities (what) that you believe might address this constraint.

____

Are any of these actions that you identified currently being taken, or are efforts underway to take these actions?

○ Yes

○ No

○ Unsure

○ Prefer not to answer

Do you have any information on the costs associated with this solution or these solutions?

Note: We do not ask about any cost information in this survey.

- ○ Yes
- ○ No
- ○ Unsure
- ○ Prefer not to answer

Page Break

Thinking only about your project, how the constraint related to "limited social learning or exchange networks for NCS" be overcome or at least mitigated?

Please provide your answer in one to three sentences and identify the actors (who) and the activities (what) that you believe might address this constraint.

____

Are any of these actions that you identified currently being taken, or are efforts underway to take these actions?

- ○ Yes
- ○ No
- ○ Unsure
- ○ Prefer not to answer

Do you have any information on the costs associated with this solution or these solutions?

Note: We do not ask about any cost information in this survey.

- ○ Yes
- ○ No
- ○ Unsure
- ○ Prefer not to answer

Page Break

Thinking only about your project, how the constraint related to "difficulty identifying, engaging, or coordinating with relevant actors" be overcome or at least mitigated?

Please provide your answer in one to three sentences and identify the actors (who) and the activities (what) that you believe might address this constraint.

____

Are any of these actions that you identified currently being taken, or are efforts underway to take these actions?

- ○ Yes
- ○ No
- ○ Unsure
- ○ Prefer not to answer

Do you have any information on the costs associated with this solution or these solutions?

Note: We do not ask about any cost information in this survey.

- ◯ Yes
- ◯ No
- ◯ Unsure
- ◯ Prefer not to answer

Page Break

Thinking only about your project, how might the constraint related to "insecure or uncertain rights to manage or sell property" be overcome or at least mitigated?

Please provide your answer in one to three sentences and identify the actors (who) and the activities (what) that you believe might address this constraint.

____

Are any of these actions that you identified currently being taken, or are efforts underway to take these actions?

- ◯ Yes
- ◯ No
- ◯ Unsure
- ◯ Prefer not to answer

Do you have any information on the costs associated with this solution or these solutions?

Note: We do not ask about any cost information in this survey.

- ○ Yes
- ○ No
- ○ Unsure
- ○ Prefer not to answer

Page Break

Thinking only about your project, how might the constraint related to "insecure, uncertain, or lack of rights to use natural resources" be overcome or at least mitigated?

Please provide your answer in one to three sentences and identify the actors (who) and the activities (what) that you believe might address this constraint.

____

Are any of these actions that you identified currently being taken, or are efforts underway to take these actions?

- ○ Yes
- ○ No
- ○ Unsure
- ○ Prefer not to answer

Do you have any information on the costs associated with this solution or these solutions?

Note: We do not ask about any cost information in this survey.

○ Yes

○ No

○ Unsure

○ Prefer not to answer

Page Break

Thinking only about your project, how might the constraint related to "regulatory barriers to production, transport, or sale of NCS outputs (for example, permits or licensing)" be overcome or at least mitigated?

Please provide you answer in one to three sentences and identify the actors (who) and the activities (what) that you believe might address this constraint.

____

Are any of these actions that you identified currently being taken, or are efforts underway to take these actions?

○ Yes

○ No

○ Unsure

○ Prefer not to answer

Do you have any information on the costs associated with this solution or these solutions?

Note: We do not ask about any cost information in this survey.

- ○ Yes
- ○ No
- ○ Unsure
- ○ Prefer not to answer

Page Break

Thinking only about your project, how might the constraint related to "unsecure or uncertain NCS benefit sharing" be overcome or at least mitigated?

Please provide you answer in one to three sentences and identify the actors (who) and the activities (what) that you believe might address this constraint.

____

Are any of these actions that you identified currently being taken, or are efforts underway to take these actions?

- ○ Yes
- ○ No
- ○ Unsure
- ○ Prefer not to answer

Do you have any information on the costs associated with this solution or these solutions?

Note: We do not ask about any cost information in this survey.

- ◯ Yes
- ◯ No
- ◯ Unsure
- ◯ Prefer not to answer

Page Break

Thinking only about your project, how might the constraint related to "NCS-related corruption" be overcome or at least mitigated?

Please provide your answer in one to three sentences and identify the actors (who) and the activities (what) that you believe might address this constraint.

____

Are any of these actions that you identified currently being taken, or are efforts underway to take these actions?

- ◯ Yes
- ◯ No
- ◯ Unsure
- ◯ Prefer not to answer

Do you have any information on the costs associated with this solution or these solutions?

Note: We do not ask about any cost information in this survey.

- ○ Yes
- ○ No
- ○ Unsure
- ○ Prefer not to answer

Page Break

Thinking only about your project, how might the constraint related to "unclear laws and policies related to NCS outputs/markets" be overcome or at least mitigated?

Please provide your answer in one to three sentences and identify the actors (who) and the activities (what) that you believe might address this constraint.

____

Are any of these actions that you identified currently being taken, or are efforts underway to take these actions?

- ○ Yes
- ○ No
- ○ Unsure
- ○ Prefer not to answer

Do you have any information on the costs associated with this solution or these solutions?

Note: We do not ask about any cost information in this survey.

- ◯ Yes
- ◯ No
- ◯ Unsure
- ◯ Prefer not to answer

Page Break

Thinking only about your project, how might the constraint related to "lack of policy coordination (for example, between sectors or between different administrative units)" be overcome or at least mitigated?

Please provide your answer in one to three sentences and identify the actors (who) and the activities (what) that you believe might address this constraint.

____

Are any of these actions that you identified currently being taken, or are efforts underway to take these actions?

- ◯ Yes
- ◯ No
- ◯ Unsure
- ◯ Prefer not to answer

Do you have any information on the costs associated with this solution or these solutions?

Note: We do not ask about any cost information in this survey.

- ○ Yes
- ○ No
- ○ Unsure
- ○ Prefer not to answer

Page Break

Thinking only about your project, how might the constraint related to "uncertain enforcement of environmental laws" be overcome or at least mitigated?

Please provide your answer in one to three sentences and identify the actors (who) and the activities (what) that you believe might address this constraint.

____

Are any of these actions that you identified currently being taken, or are efforts underway to take these actions?

- ○ Yes
- ○ No
- ○ Unsure
- ○ Prefer not to answer

Do you have any information on the costs associated with this solution or these solutions?

Note: We do not ask about any cost information in this survey.

- ◯ Yes
- ◯ No
- ◯ Unsure
- ◯ Prefer not to answer

Page Break

Thinking only about your project, how might the constraint related to "weak monitoring and enforcement of NCS agreements" be overcome or at least mitigated?

Please provide your answer in one to three sentences and identify the actors (who) and the activities (what) that you believe might address this constraint.

____

Are any of these actions that you identified currently being taken, or are efforts underway to take these actions?

- ◯ Yes
- ◯ No
- ◯ Unsure
- ◯ Prefer not to answer

Do you have any information on the costs associated with this solution or these solutions?

Note: We do not ask about any cost information in this survey.

◯ Yes

◯ No

◯ Unsure

◯ Prefer not to answer

Page Break

Thinking only about your project, how might the constraint related to "violent conflict or the perceived threat of violence" be overcome or at least mitigated?

Please provide your answer in one to three sentences and identify the actors (who) and the activities (what) that you believe might address this constraint.

____

Are any of these actions that you identified currently being taken, or are efforts underway to take these actions?

◯ Yes

◯ No

◯ Unsure

◯ Prefer not to answer

Do you have any information on the costs associated with this solution or these solutions?

Note: We do not ask about any cost information in this survey.

- ◯ Yes
- ◯ No
- ◯ Unsure
- ◯ Prefer not to answer

Page Break

Thinking only about your project, how might the constraint related to "lack of dispute resolution mechanisms" be overcome or at least mitigated?

Please provide your answer in one to three sentences and identify the actors (who) and the activities (what) that you believe might address this constraint.

____

Are any of these actions that you identified currently being taken, or are efforts underway to take these actions?

- ◯ Yes
- ◯ No
- ◯ Unsure
- ◯ Prefer not to answer

Do you have any information on the costs associated with this solution or these solutions?

Note: We do not ask about any cost information in this survey.

○ Yes

○ No

○ Unsure

○ Prefer not to answer

Page Break

Thinking only about your project, how might the constraint related to "financial or other incentives for non-NCS (for example, subsidies, reduced taxes, access to credit or insurance)" be overcome or at least mitigated?

Please provide your answer in one to three sentences and identify the actors (who) and the activities (what) that you believe might address this constraint.

____

Are any of these actions that you identified currently being taken, or are efforts underway to take these actions?

○ Yes

○ No

○ Unsure

○ Prefer not to answer

Do you have any information on the costs associated with this solution or these solutions?

Note: We do not ask about any cost information in this survey.

- ○ Yes
- ○ No
- ○ Unsure
- ○ Prefer not to answer

Page Break

Thinking only about your project, how might the constraint related to "politically influential interests favoring non-NCS" be overcome or at least mitigated?

Please provide your answer in one to three sentences and identify the actors (who) and the activities (what) that you believe might address this constraint.

____

Are any of these actions that you identified currently being taken, or are efforts underway to take these actions?

- ○ Yes
- ○ No
- ○ Unsure
- ○ Prefer not to answer

Do you have any information on the costs associated with this solution or these solutions?

Note: We do not ask about any cost information in this survey.

- ◯ Yes
- ◯ No
- ◯ Unsure
- ◯ Prefer not to answer

Page Break

Thinking only about your project, how might the constraint related to "other constraint 1: *[respondent's write-in constraint 1]*" be overcome or at least mitigated?

Please provide your answer in one to three sentences and identify the actors (who) and the activities (what) that you believe might address this constraint.

____

Are any of these actions that you identified currently being taken, or are efforts underway to take these actions?

- ◯ Yes
- ◯ No
- ◯ Unsure
- ◯ Prefer not to answer

Do you have any information on the costs associated with this solution or these solutions?

Note: We do not ask about any cost information in this survey.

◯ Yes

◯ No

◯ Unsure

◯ Prefer not to answer

Page Break

Thinking only about your project, how might the constraint related to "other constraint 2: *[respondent's write-in constraint 2]*" be overcome or at least mitigated?

Please provide your answer in one to three sentences and identify the actors (who) and the activities (what) that you believe might address this constraint.

____

Are any of these actions that you identified currently being taken, or are efforts underway to take these actions?

◯ Yes

◯ No

◯ Unsure

◯ Prefer not to answer

Do you have any information on the costs associated with this solution or these solutions?

Note: We do not ask about any cost information in this survey.

- ◯ Yes
- ◯ No
- ◯ Unsure
- ◯ Prefer not to answer

Page Break

Thinking only about your project, how might the constraint related to "other constraint 3: *[respondent's write-in constraint 3]*" be overcome or at least mitigated?

Please provide your answer in one to three sentences and identify the actors (who) and the activities (what) that you believe might address this constraint.

____

Are any of these actions that you identified currently being taken, or are efforts underway to take these actions?

- ◯ Yes
- ◯ No
- ◯ Unsure
- ◯ Prefer not to answer

Do you have any information on the costs associated with this solution or these solutions?

Note: We do not ask about any cost information in this survey.

◯ Yes

◯ No

◯ Unsure

◯ Prefer not to answer

Page Break

End of Block: Module 3: Solutions to Constraints

Start of Block: Module 4: Enabling Conditions

By how much do you think your project could increase in size in the next ten (10) years if key enabling conditions were fulfilled?

◯ None

◯ Double in size (2x)

◯ Triple in size (3x)

◯ Quadruple in size (4x)

◯ Quintuple in size or more (≥5x)

◯ Unsure/prefer not to answer

Please identify up to ten conditions that are most important for enabling your project to ***[respondent's scale choice above]*****, but that currently would prevent project scale-up.**

An increase in project size may refer to implementing protection, restoration, or improved management across a greater area, or it may refer to working with more communities or individual land managers/operators. ***The conditions you select below may already be fulfilled for your project at its current size, but by selecting them you indicate that they are not fulfilled for increasing your project's current size***.

Leave all other conditions unselected.

	Conditions of highest importance for increasing current project size	Conditions not of highest importance for increasing current project size
Sufficient quantity and quality of, or affordable cost of, planting stock and other materials for NCS	○	○
Sufficient quantity and quality of, or affordable cost of, labor (external or own) for NCS	○	○
Sufficient quantity and quality of, or affordable cost of, land for NCS	○	○
Sufficient quantity and quality of, or affordable cost of, water for NCS	○	○
Availability of, or acceptable cost of, credit for land managers/operators to participate in NCS	○	○
Availability of, or acceptable cost of, other funding for land managers/operators to participate in NCS	○	○
Availability of, or acceptable cost of, insurance for land managers/operators to implement NCS	○	○
Availability of, or acceptable cost of, credit for NCS projects	○	○
Availability of, or acceptable cost of, other funding for NCS projects	○	○
Strong donor/creditor relationships or reporting	○	○
Availability of markets for NCS outputs (for example, food or wood, etc.) produced by the land manager/operator	○	○
Availability of markets for carbon sequestered by the NCS	○	○
Availability of markets for ecosystem services (for example, water quality or flow regulation) or biodiversity provided by the NCS	○	○

Reasonable prices for NCS outputs (for example, food or wood) produced by the land manager/operator	○	○
Reasonable prices for carbon sequestered by the NCS	○	○
Reasonable prices for ecosystem services (for example, water quality or flow regulation) or biodiversity provided by the NCS	○	○
Profitability of NCS comparable to that of alternative land uses (please specify)	○	○
High levels of land manager/operator literacy, numeracy, or technological capabilities	○	○
Availability of, or reasonable cost of, information about how to design or begin the NCS	○	○
Availability of, or reasonable cost of, information about how to manage the NCS (for example, maintenance activities or other inputs and their timing)	○	○
Availability of, or reasonable cost of, technical advice to land operators (for example, extension services)	○	○
Availability of, or reasonable cost of, information about yields, inputs and profits	○	○
Availability of, or reasonable cost of, information about market access or prices	○	○
Availability of, or reasonable cost of, information about on-site benefits of NCS (for example, soil fertility, shade for livestock, or income diversification)	○	○
Absence of preferences for non-NCS land uses among key decisionmakers	○	○

No aversion to trying new land uses among key decisionmakers	○	○
No skepticism toward NCS or lack of trust in NCS promoters	○	○
Absence of social norms that favor non-NCS land uses	○	○
Ways to address negative equity impacts of NCS	○	○
Opportunity to participate in, or influence the implementation of NCS for people of all genders, races, ethnicities, or other dimensions of identity	○	○
Available social learning or exchange networks for NCS	○	○
Ability to identify, engage, or coordinate with relevant actors	○	○
Secure or certain rights to manage or sell property	○	○
Secure or certain rights to use natural resources	○	○
Absence of, or low regulatory barriers to production, transport or sale of NCS outputs (for example, permits or licensing requirements)	○	○
Secure or certain NCS benefit sharing	○	○
Absence of NCS-related corruption	○	○
Clear laws and policies related to NCS outputs/markets	○	○
Effective policy coordination (for example, between sectors or between different administrative units)	○	○
Reliable enforcement of environmental laws	○	○

Reliable monitoring and enforcement of NCS agreements	○	○
Absence of violent conflict or the perceived threat of violence	○	○
Availability of dispute resolution mechanisms	○	○
Absence of financial or other incentives for non-NCS (for example, subsidies, reduced taxes, access to credit or insurance)	○	○
Absence of politically influential interests favoring non-NCS	○	○
Other enabling conditions (please specify)	○	○
Other enabling conditions (please specify)	○	○
Other enabling conditions (please specify)	○	○

End of Block: Module 4: Enabling Conditions

Start of Block: Module 5

These are the final two questions for this survey.

If you have anything you would like to add, please use the space below.

____

If you would like to receive information about the results of this survey and related research, please enter your email below.

____

End of Block: Module

Supplementary Text References

1. Roe, S. *et al.* Land-based measures to mitigate climate change: Potential and feasibility by country. *Global Change Biology* **27**, 6025–6058 (2021).
2. Agriculture, Forestry and Other Land Uses (AFOLU). Contribution of Working Group III to the Sixth Assessment Report of the Intergovernmental Panel on Climate Change. in *Climate Change 2022 - Mitigation of Climate Change.* (ed. Intergovernmental Panel On Climate Change (IPCC)) 747–860 (Cambridge University Press, 2023). doi:10.1017/9781009157926.009.
3. Schulte, I., Eggers, J., Nielsen, J. Ø. & Fuss, S. What influences the implementation of natural climate solutions? A systematic map and review of the evidence. *Environ. Res. Lett.* **17**, 013002 (2022).
4. Karki, L. *et al.* Potentials and barriers to land-based mitigation technologies and practices (LMTs)—a review. *Environ. Res. Lett.* **18**, 093003 (2023).
5. Intergovernmental Panel on Climate Change. *Climate Change 2014: Mitigation of Climate Change: Working Group III Contribution to the IPCC Fifth Assessment Report*. (Cambridge University Press, 2015). doi:10.1017/CBO9781107415416.
6. Brumberg, H. *et al.* Global analysis of constraints to natural climate solution implementation. *PNAS Nexus* **4**, pgaf173 (2025).
7. R Core Team. R: A Language and Environment for Statistical Computing. R Foundation for Statistical Computing (2023).
8. Appleton, M. R. *et al.* Protected area personnel and ranger numbers are insufficient to deliver global expectations. *Nat Sustain* **5**, 1100–1110 (2022).
