# Supplementary material for: Global literature review and survey of implementation constraints on natural climate solutions
Source: Nat Commun. 2026 Mar 14;17:4580. doi: 10.1038/s41467-026-70482-4 (PMC13195100; doi:10.1038/s41467-026-70482-4)
Supplement: Supplementary file 2 — Reporting Summary [file 41467_2026_70482_MOESM2_ESM.pdf]

nature portfolio

Corresponding author(s): Timm Kroeger

Last updated by author(s): Dec 9, 2025

Reporting Summary

Nature Portfolio wishes to improve the reproducibility of the work that we publish. This form provides structure for consistency and transparency in reporting. For further information on Nature Portfolio policies, see our Editorial Policies and the Editorial Policy Checklist.

Statistics

For all statistical analyses, confirm that the following items are present in the figure legend, table legend, main text, or Methods section.

n/a	Confirmed	
☐	☒	The exact sample size (*n*) for each experimental group/condition, given as a discrete number and unit of measurement
☐	☒	A statement on whether measurements were taken from distinct samples or whether the same sample was measured repeatedly
☒	☐	The statistical test(s) used AND whether they are one- or two-sided<br>*Only common tests should be described solely by name; describe more complex techniques in the Methods section.*
☒	☐	A description of all covariates tested
☒	☐	A description of any assumptions or corrections, such as tests of normality and adjustment for multiple comparisons
☐	☒	A full description of the statistical parameters including central tendency (e.g. means) or other basic estimates (e.g. regression coefficient) AND variation (e.g. standard deviation) or associated estimates of uncertainty (e.g. confidence intervals)
☒	☐	For null hypothesis testing, the test statistic (e.g. *F*, *t*, *r*) with confidence intervals, effect sizes, degrees of freedom and *P* value noted<br>*Give P values as exact values whenever suitable.*
☒	☐	For Bayesian analysis, information on the choice of priors and Markov chain Monte Carlo settings
☒	☐	For hierarchical and complex designs, identification of the appropriate level for tests and full reporting of outcomes
☒	☐	Estimates of effect sizes (e.g. Cohen's *d*, Pearson's *r*), indicating how they were calculated

Our web collection on statistics for biologists contains articles on many of the points above.

Software and code

Policy information about availability of computer code

Data collection	Qualtrix CoreXM (online survey software);
Data analysis	R (2023); R tidyverse, dplyr, and ggplot2 packages; Excel

For manuscripts utilizing custom algorithms or software that are central to the research but not yet described in published literature, software must be made available to editors and reviewers. We strongly encourage code deposition in a community repository (e.g. GitHub). See the Nature Portfolio guidelines for submitting code & software for further information.

Data

Policy information about availability of data

All manuscripts must include a data availability statement. This statement should provide the following information, where applicable:

- Accession codes, unique identifiers, or web links for publicly available datasets
- A description of any restrictions on data availability
- For clinical datasets or third party data, please ensure that the statement adheres to our policy

Data availability: The geo-referenced database generated in this study has been deposited in the Harvard Dataverse Repository under accession code https://doi.org/10.7910/DVN/EDZHBF. Analysis code: The analysis code and the code used to produce the figures are available in the Harvard Dataverse Repository at https://doi.org/10.7910/DVN/EDZHBF.

Research involving human participants, their data, or biological material

Policy information about studies with human participants or human data. See also policy information about sex, gender (identity/presentation), and sexual orientation and race, ethnicity and racism.

Reporting on sex and gender	We do not analyze or report on sex or gender of survey participants. The survey targeted managers of projects that implement natural climate solutions, with participants self-selecting into the survey.
Reporting on race, ethnicity, or other socially relevant groupings	We do not analyze or report on race, ethnicity, or other socially relevant groupings of survey participants. The survey targeted managers of projects that implement natural climate solutions, with participants self-selecting into the survey.
Population characteristics	We do not analyze or report on population characteristics of survey participants. The survey targeted managers of projects that implement natural climate solutions, with participants self-selecting into the survey.
Recruitment	The survey was distributed by the authors' institutions to projects that implement actions that qualify as natural climate solutions, and was promoted in several editions of the Restor (www.restor.eco) and Nature4Climate (https://nature4climate.org) online newsletters.. Participants self-selected into the survey.
Ethics oversight	The survey was approved under The Nature Conservancy's Human Subjects Research review policy, managed by the Chief Scientist's Office. Approval was given on June 16, 2022, prior to they survey rollout. The approval form is available upon request.

Note that full information on the approval of the study protocol must also be provided in the manuscript.

Field-specific reporting

Please select the one below that is the best fit for your research. If you are not sure, read the appropriate sections before making your selection.

☐ Life sciences ☐ Behavioural & social sciences ☒ Ecological, evolutionary & environmental sciences

For a reference copy of the document with all sections, see nature.com/documents/nr-reporting-summary-flat.pdf

Ecological, evolutionary & environmental sciences study design

All studies must disclose on these points even when the disclosure is negative.

Study description	We conducted a systematic review of published academic papers that identify constraints on the implementation of actions that represent natural climate solutions, and implemented a global survey of projects that implement natural climate solutions -- regardless of whether climate mitigation or adaptation are stated project objectives -- that queried participants about the constraints that affect the successful implementation of their project.
Research sample	347 peer-reviewed articles reporting implementation constraints on natural climate solutions and published in 2020 or 2021, and 154 projects that currently implement natural climate solutions. The literature sample is a complete sample of the scientific papers that fit our exclusion criteria; the project sample is a convenience sample (for reasons described in the Sampling strategy) and is expected to represent the types of Natural Climate Solutions implemented by local governments, communities, or local and international not-for-profit organizations.
Sampling strategy	The literature review sample included all original research articles featured in the Web of Science or Scopus databases that identified an implementation constraint for a natural climate solution, were not meta-analyses, and were published in 2020 or 2021 (for full description, see Brumberg et al. 2025, 'Global analysis of constraints to natural climate solution implementation', PNAS Nexus 4, pgaf173). The survey sample resulted from self-selection into the study of participants who were invited to participate in the survey by the contact networks of the institutions of the authors or after learning about the survey in online newsletters published by Restor (restor.eco) and Nature4Climate (https://nature4climate.org). This sampling choice was the only available option because there is no comprehensive database of projects that implement natural climate solutions.
Data collection	The literature data collection employed separate searches in Scopus and Web of Science and was implemented by JTE and TK. Articles were pre-screened and fully coded by HB, MH, WE, andAL with intercoder reliability testing performed during pre-screening and coding (see Brumberg et al. 2025 for details). TK, HB, MH, WE and AL recoded all constraints observations reported in Brumberg et al. (2025) to the expanded constraints set used in the present study. JTE and TK developed the survey with the input of all authors, pre-tested the survey with 22 TNC field practitioners and program managers, programmed the online survey into the Qualtrix CoreXM platform, and performed extensive functionality testing prior to survey release. JTE extracted the survey responses, and ZL and JTE performed data analysis in R. TK performed additional analyses in Excel.
Timing and spatial scale	The literature data were collected on November 4, 2021. The survey data were collected during October 14, 2022 and December 31, 2023.
Data exclusions	Survey responses that did not complete the survey modules on project characteristics and constraints were excluded from the analysis.

Reproducibility	N/A
Randomization	Given the nature of the survey distribution (diffusion through the authors' institutions' networks, publication in two high-circulation online newsletters), no randomization of participants was possible.
Blinding	N/A

Did the study involve field work? ☐ Yes ☒ No

Reporting for specific materials, systems and methods

We require information from authors about some types of materials, experimental systems and methods used in many studies. Here, indicate whether each material, system or method listed is relevant to your study. If you are not sure if a list item applies to your research, read the appropriate section before selecting a response.

Materials & experimental systems

n/a	Involved in the study	
☒	☐	Antibodies
☒	☐	Eukaryotic cell lines
☒	☐	Palaeontology and archaeology
☒	☐	Animals and other organisms
☒	☐	Clinical data
☒	☐	Dual use research of concern
☒	☐	Plants

Methods

n/a	Involved in the study	
☒	☐	ChIP-seq
☒	☐	Flow cytometry
☒	☐	MRI-based neuroimaging

Plants

Seed stocks	N/A
Novel plant genotypes	N/A
Authentication	N/A
